# Supplementary figures and images for: CD47 blockade (ALX301) enhances immunoradiotherapy response in HPV negative head and neck squamous cell carcinoma
Source: PLoS One. 2026 Feb 17;21(2):e0328031. doi: 10.1371/journal.pone.0328031 (PMC12912607; doi:10.1371/journal.pone.0328031)

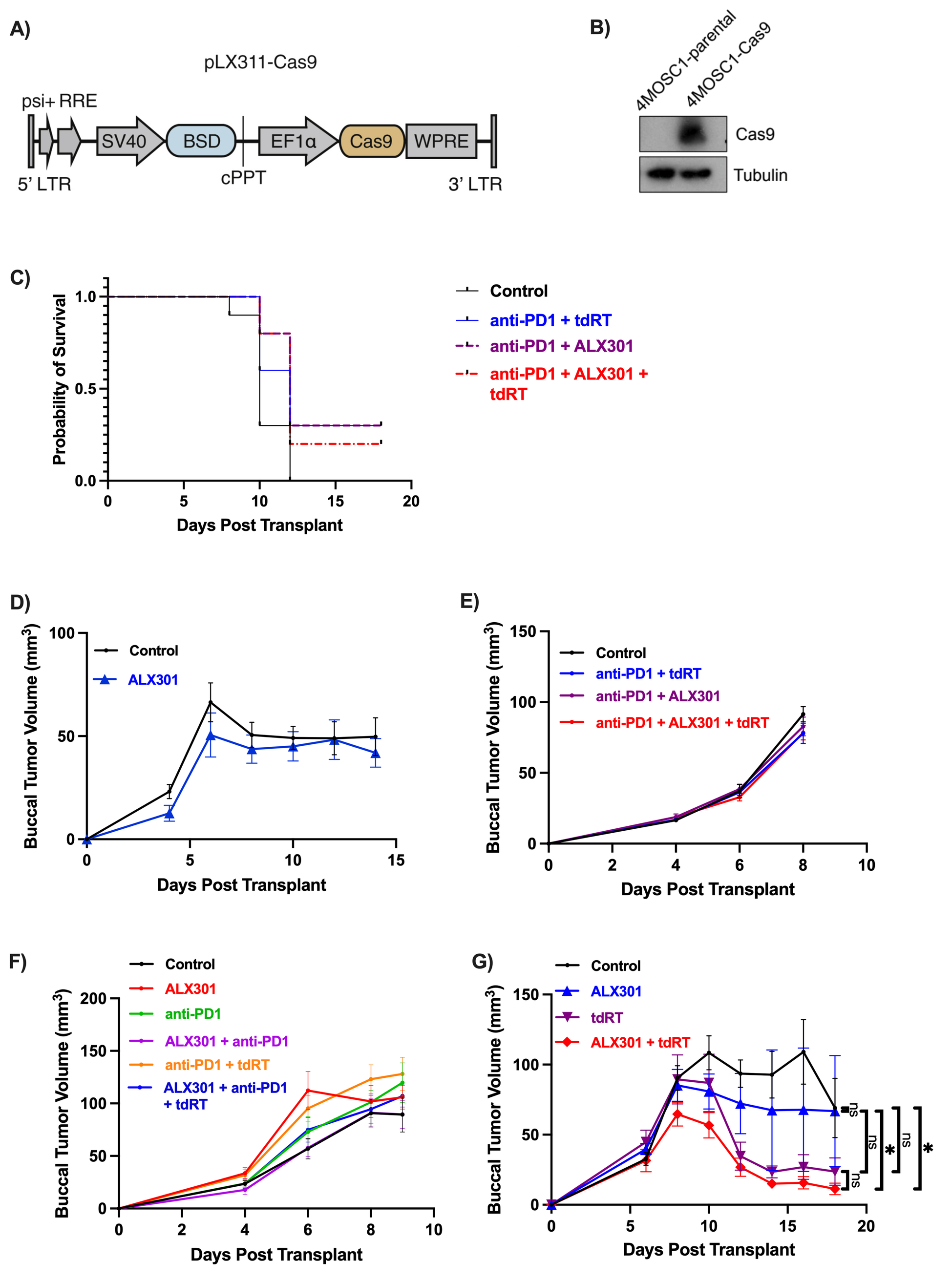

Supplement: S1 Fig — (B) Verification of Cas9 expression in 4MOSC1 using western blot. (C) Kaplan Meier curve demonstrating the survivability from the experiment depicted in Fig 2C (n = 10 for each arm, Control vs. anti-PD1 + tdRT p = 0.0505, Control vs. anti-PD1 + ALX301 p = 0.0114*, Control vs. anti-PD1 + ALX301 + tdRT p = 0.016*) where with the treatment of ALX301, there is a statistically significant increase in survivability. p values were calculated using a Log-rank test. (D) Representative tumor growth kinetics of mice with 4MOSC1 tumors treated with vehicle (control) vs. ALX301. Data are presented as mean ± SEM (n = 8 for control, n = 7 for ALX301). (E) Representative tumor growth kinetics of mice with 4MOSC2 tumors treated with vehicle (control), anti-PD1 + tdRT, anti-PD1 + ALX301, and anti-PD1 + ALX301 + tdRT. Data are presented as mean ± SEM (For control: n = 10 up to day 6, n = 9 on day 8. One mouse was omitted due to succumbing to disease. n = 10 for all other groups). (F) Representative tumor growth kinetics of mice with 4MOSC2 tumors treated with vehicle (control), ALX301, anti-PD1, ALX301 + anti-PD1, anti-PD1 + tdRT, and ALX301 + anti-PD1 + tdRT. Data are presented as mean ± SEM (n = 10 for control, n = 8 for all other groups). (G) Representative tumor growth kinetics of mice with 4MOSC2 tumors treated with vehicle (control), ALX301, tdRT, and ALX301 + tdRT (p = 0.9996 for Control vs. ALX301, p = 0.1841 for Control vs. tdRT, *p = 0.0188 for Control vs. ALX301 + tdRT, p = 0.2671 for ALX301 vs. tdRT, *p = 0.0436 for ALX301 vs. ALX301 + tdRT, p = 0.9341 for tdRT vs. ALX301 + tdRT). Data are presented as mean ± SEM; p values were calculated using ordinary two-way ANOVA with Tukey’s post-hoc (For control: n = 8 up to day 10, n = 6 on day 12, n = 5 on days 14–18. For ALX301: n = 8 up to day 10, n = 7 on day 12, n = 4 on days 14–18. For tdRT: n = 7 up to day 10, n = 5 on day 12, n = 4 on days 14–18. For ALX301 + tdRT: n = 7 up to day 18). Mice were omitted due to succumbing to [file pone.0328031.s001.tif]
