# Supplementary material for: CD47 blockade (ALX301) enhances immunoradiotherapy response in HPV negative head and neck squamous cell carcinoma
Source: PLoS One. 2026 Feb 17;21(2):e0328031. doi: 10.1371/journal.pone.0328031 (PMC12912607; doi:10.1371/journal.pone.0328031)
Supplement: S1 Raw Images — (PDF) [file pone.0328031.s002.pdf]

Raw western blot image corresponding to S1 Fig 1B

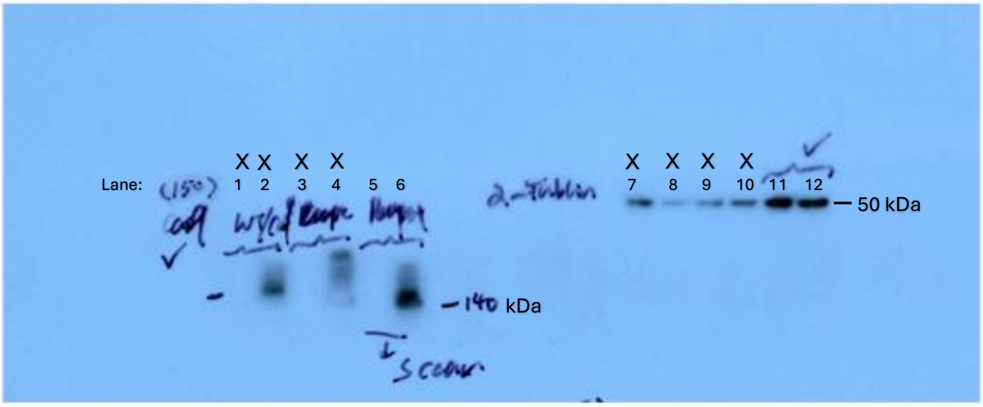

Loading Order:

Lanes 1, 3, 5, 7, 9, and 11: Wild type 4MOSC1 parental cells without Cas9 expression.

Lanes 2, 4, 6, 8, 10, and 12: 4MOSC1 cells with Cas9 expression.
