# Supplementary material for: CD47 blockade (ALX301) enhances immunoradiotherapy response in HPV negative head and neck squamous cell carcinoma
Source: PLoS One. 2026 Feb 17;21(2):e0328031. doi: 10.1371/journal.pone.0328031 (PMC12912607; doi:10.1371/journal.pone.0328031)
Supplement: S2 File — (DOCX) [file pone.0328031.s003.docx]

**Reporting of Statistical Results**

**Figure 1B**

| **Days** | **Parent** | **Parent** | **Parent** | **Parent** | **Parent** | **Parent** | **Parent** | **Parent** | **Parent** | **Parent** | **Cas9** | **Cas9** | **Cas9** | **Cas9** | **Cas9** | **Cas9** | **Cas9** | **Cas9** | **Cas9** | **Cas9** |
| --- | --- | --- | --- | --- | --- | --- | --- | --- | --- | --- | --- | --- | --- | --- | --- | --- | --- | --- | --- | --- |
| **0** | 0.0000000 | 0.0000000 | 0.0000000 | 0.0000000 | 0.0000000 | 0.0000000 | 0.0000000 | 0.0000000 | 0.0000000 | 0.0000000 | 0.0000000 | 0.0000000 | 0.0000000 | 0.0000000 | 0.0000000 | 0.0000000 | 0.0000000 | 0.0000000 | 0.0000000 | 0.0000000 |
| **4** | 15.7500000 | 19.4400000 | 21.6600000 | 13.5000000 | 13.5000000 | 21.6600000 | 18.3750000 | 27.4360000 | 13.5000000 | 18.3750000 | 19.6000000 | 23.9575000 | 15.3600000 | 13.5000000 | 13.5000000 | 15.3600000 | 28.0000000 | 18.3750000 | 18.3750000 | 20.5350000 |
| **7** | 54.0225000 | 83.7225000 | 54.0800000 | 45.5625000 | 31.3875000 | 51.2500000 | 40.5000000 | 65.0250000 | 37.5000000 | 56.2500000 | 48.0200000 | 60.0000000 | 50.0000000 | 43.7500000 | 13.5000000 | 48.3840000 | 28.0000000 | 45.5625000 | 40.5000000 | 46.0800000 |
| **9** | 108.0000000 | 105.8750000 | 90.0000000 | 39.0150000 | 32.0000000 | 58.5225000 | 60.0000000 | 86.2400000 | 43.7500000 | 72.0000000 | 40.5000000 | 72.0000000 | 21.6600000 | 63.0000000 | 13.5000000 | 56.2500000 | 49.2205000 | 60.0000000 | 68.0625000 | 72.1280000 |
| **11** | 106.2000000 | 194.4000000 | 90.0000000 | 56.8620000 | 28.0000000 | 62.7200000 | 90.7500000 | 89.3475000 | 30.3750000 | 99.0000000 | 32.0000000 | 68.0625000 | 9.3750000 | 69.6200000 | 13.5000000 | 9.0000000 | 7.8125000 | 46.0800000 | 33.6200000 | 56.2500000 |
| **13** | 91.8000000 | 126.0000000 | 58.5225000 | 30.3750000 | 29.6000000 | 72.0000000 | 87.0250000 | 90.0000000 | 42.0175000 | 88.2000000 | 4.0000000 | 28.0000000 | 0.0000000 | 50.7195000 | 0.0000000 | 4.0000000 | 4.0000000 | 40.5000000 | 56.2500000 | 62.5000000 |
| **15** | 91.8000000 | 147.0000000 | 88.2000000 | 24.0000000 | 21.4375000 | 73.8000000 | 108.0000000 | 94.8855000 | 35.4375000 | 101.4000000 | 0.0000000 | 13.5000000 | 0.0000000 | 43.7500000 | 0.0000000 | 0.0000000 | 0.0000000 | 35.4375000 | 45.5625000 | 61.2500000 |

| **Table Analyzed** | **Exp1 CD47KO_Cas9 vs Parent** |  |  |  |  |
| --- | --- | --- | --- | --- | --- |
|  |  |  |  |  |  |
| **Two-way RM ANOVA** | Matching: Stacked |  |  |  |  |
| **Assume sphericity?** | No |  |  |  |  |
| **Alpha** | 0.05 |  |  |  |  |
|  |  |  |  |  |  |
| **Source of Variation** | % of total variation | P value | P value summary | Significant? | Geisser-Greenhouse's epsilon |
| **Time x Column Factor** | 10.57 | <0.0001 | **** | Yes |  |
| **Time** | 34.41 | <0.0001 | **** | Yes | 0.3070 |
| **Column Factor** | 13.31 | 0.0051 | ** | Yes |  |
| **Subject** | 23.54 | <0.0001 | **** | Yes |  |
|  |  |  |  |  |  |
| **ANOVA table** | SS | DF | MS | F (DFn, DFd) | P value |
| **Time x Column Factor** | 18906 | 6 | 3151 | F (6, 108) = 10.48 | P<0.0001 |
| **Time** | 61532 | 6 | 10255 | F (1.842, 33.16) = 34.10 | P<0.0001 |
| **Column Factor** | 23805 | 1 | 23805 | F (1, 18) = 10.18 | P=0.0051 |
| **Subject** | 42087 | 18 | 2338 | F (18, 108) = 7.774 | P<0.0001 |
| **Residual** | 32483 | 108 | 300.8 |  |  |
|  |  |  |  |  |  |
| **Difference between column means** |  |  |  |  |  |
| **Mean of Parent** | 53.53 |  |  |  |  |
| **Mean of Cas9** | 27.45 |  |  |  |  |
| **Difference between means** | 26.08 |  |  |  |  |
| **SE of difference** | 8.173 |  |  |  |  |
| **95% CI of difference** | 8.908 to 43.25 |  |  |  |  |
|  |  |  |  |  |  |
| **Data summary** |  |  |  |  |  |
| **Number of columns (Column Factor)** | 2 |  |  |  |  |
| **Number of rows (Time)** | 7 |  |  |  |  |
| **Number of subjects (Subject)** | 20 |  |  |  |  |
| **Number of missing values** | 0 |  |  |  |  |

**Figure 1C**

| **CD47 KO** | **CD47 KO** | **CD47 KO** | **CD47 KO** | **CD47 KO** | **FMO Isotype Control (-)** | **FMO Isotype Control (-)** | **FMO Isotype Control (-)** | **sgRNA (+)** | **sgRNA (+)** | **sgRNA (+)** | **sgRNA (+)** | **sgRNA (+)** | **Parent 4MOSC1** | **Parent 4MOSC1** | **Parent 4MOSC1** | **Parent 4MOSC1** | **Parent 4MOSC1** |
| --- | --- | --- | --- | --- | --- | --- | --- | --- | --- | --- | --- | --- | --- | --- | --- | --- | --- |
| 173 | 175 | 184 | 197 | 187 | 162 | 158 | 159 | 246 | 271 | 273 | 319 | 285 | 171 | 253 | 215 | 164 | 313 |

| **Number of families** | **1** |  |  |  |  |  |  |  |
| --- | --- | --- | --- | --- | --- | --- | --- | --- |
| **Number of comparisons per family** | 3 |  |  |  |  |  |  |  |
| **Alpha** | 0.05 |  |  |  |  |  |  |  |
|  |  |  |  |  |  |  |  |  |
| **Dunnett's multiple comparisons test** | Mean Diff. | 95.00% CI of diff. | Below threshold? | Summary | Adjusted P Value | A-? |  |  |
| **CD47 KO vs. FMO Isotype Control (-)** | 23.53 | -46.51 to 93.58 | No | ns | 0.7183 | B | FMO Isotype Control (-) |  |
| **CD47 KO vs. sgRNA (+)** | -95.60 | -156.3 to -34.94 | Yes | ** | 0.0026 | C | sgRNA (+) |  |
| **CD47 KO vs. Parent 4MOSC1** | -40.00 | -100.7 to 20.66 | No | ns | 0.2412 | D | Parent 4MOSC1 |  |
|  |  |  |  |  |  |  |  |  |
| **Test details** | Mean 1 | Mean 2 | Mean Diff. | SE of diff. | n1 | n2 | q | DF |
| **CD47 KO vs. FMO Isotype Control (-)** | 183.2 | 159.7 | 23.53 | 26.52 | 5 | 3 | 0.8874 | 14 |
| **CD47 KO vs. sgRNA (+)** | 183.2 | 278.8 | -95.60 | 22.97 | 5 | 5 | 4.163 | 14 |
| **CD47 KO vs. Parent 4MOSC1** | 183.2 | 223.2 | -40.00 | 22.97 | 5 | 5 | 1.742 | 14 |

| **Number of families** | **1** |  |  |  |  |  |  |  |
| --- | --- | --- | --- | --- | --- | --- | --- | --- |
| **Number of comparisons per family** | 6 |  |  |  |  |  |  |  |
| **Alpha** | 0.05 |  |  |  |  |  |  |  |
|  |  |  |  |  |  |  |  |  |
| **Tukey's multiple comparisons test** | Mean Diff. | 95.00% CI of diff. | Below threshold? | Summary | Adjusted P Value |  |  |  |
| **CD47 KO vs. FMO Isotype Control (-)** | 23.53 | -53.54 to 100.6 | No | ns | 0.8114 | A-B |  |  |
| **CD47 KO vs. sgRNA (+)** | -95.60 | -162.3 to -28.85 | Yes | ** | 0.0047 | A-C |  |  |
| **CD47 KO vs. Parent 4MOSC1** | -40.00 | -106.7 to 26.75 | No | ns | 0.3401 | A-D |  |  |
| **FMO Isotype Control (-) vs. sgRNA (+)** | -119.1 | -196.2 to -42.06 | Yes | ** | 0.0025 | B-C |  |  |
| **FMO Isotype Control (-) vs. Parent 4MOSC1** | -63.53 | -140.6 to 13.54 | No | ns | 0.1236 | B-D |  |  |
| **sgRNA (+) vs. Parent 4MOSC1** | 55.60 | -11.15 to 122.3 | No | ns | 0.1184 | C-D |  |  |
|  |  |  |  |  |  |  |  |  |
| **Test details** | Mean 1 | Mean 2 | Mean Diff. | SE of diff. | n1 | n2 | q | DF |
| **CD47 KO vs. FMO Isotype Control (-)** | 183.2 | 159.7 | 23.53 | 26.52 | 5 | 3 | 1.255 | 14 |
| **CD47 KO vs. sgRNA (+)** | 183.2 | 278.8 | -95.60 | 22.97 | 5 | 5 | 5.887 | 14 |
| **CD47 KO vs. Parent 4MOSC1** | 183.2 | 223.2 | -40.00 | 22.97 | 5 | 5 | 2.463 | 14 |
| **FMO Isotype Control (-) vs. sgRNA (+)** | 159.7 | 278.8 | -119.1 | 26.52 | 3 | 5 | 6.353 | 14 |
| **FMO Isotype Control (-) vs. Parent 4MOSC1** | 159.7 | 223.2 | -63.53 | 26.52 | 3 | 5 | 3.388 | 14 |
| **sgRNA (+) vs. Parent 4MOSC1** | 278.8 | 223.2 | 55.60 | 22.97 | 5 | 5 | 3.424 | 14 |
|  |  |  |  |  |  |  |  |  |
| **Compact letter display** |  |  |  |  |  |  |  |  |
| **sgRNA (+)** | A |  |  |  |  |  |  |  |
| **Parent 4MOSC1** | A B |  |  |  |  |  |  |  |
| **CD47 KO** | B |  |  |  |  |  |  |  |
| **FMO Isotype Control (-)** | B |  |  |  |  |  |  |  |

| **Number of families** | **1** |  |  |  |  |  |  |  |
| --- | --- | --- | --- | --- | --- | --- | --- | --- |
| **Number of comparisons per family** | 3 |  |  |  |  |  |  |  |
| **Alpha** | 0.05 |  |  |  |  |  |  |  |
|  |  |  |  |  |  |  |  |  |
| **Tukey's multiple comparisons test** | Mean Diff. | 95.00% CI of diff. | Below threshold? | Summary | Adjusted P Value |  |  |  |
| **CD47 KO vs. FMO Isotype Control (-)** | 23.53 | -12.34 to 59.40 | No | ns | 0.2193 | A-B |  |  |
| **CD47 KO vs. sgRNA (+)** | -95.60 | -126.7 to -64.54 | Yes | **** | <0.0001 | A-C |  |  |
| **FMO Isotype Control (-) vs. sgRNA (+)** | -119.1 | -155.0 to -83.26 | Yes | **** | <0.0001 | B-C |  |  |
|  |  |  |  |  |  |  |  |  |
| **Test details** | Mean 1 | Mean 2 | Mean Diff. | SE of diff. | n1 | n2 | q | DF |
| **CD47 KO vs. FMO Isotype Control (-)** | 183.2 | 159.7 | 23.53 | 13.08 | 5 | 3 | 2.543 | 10 |
| **CD47 KO vs. sgRNA (+)** | 183.2 | 278.8 | -95.60 | 11.33 | 5 | 5 | 11.93 | 10 |
| **FMO Isotype Control (-) vs. sgRNA (+)** | 159.7 | 278.8 | -119.1 | 13.08 | 3 | 5 | 12.88 | 10 |
|  |  |  |  |  |  |  |  |  |
| **Compact letter display** |  |  |  |  |  |  |  |  |
| **sgRNA (+)** | A |  |  |  |  |  |  |  |
| **CD47 KO** | B |  |  |  |  |  |  |  |
| **FMO Isotype Control (-)** | B |  |  |  |  |  |  |  |

**Figure 1D**

| **Days Post Transplant** | **CD47 KO** | **CD47 KO** | **CD47 KO** | **CD47 KO** | **CD47 KO** | **CD47 KO** | **CD47 KO** | **CD47 KO** | **CD47 KO** | **CD47 KO** | **Parent PD-1** | **Parent PD-1** | **Parent PD-1** | **Parent PD-1** | **Parent PD-1** | **Parent PD-1** | **Parent PD-1** | | **Parent PD-1** | **Parent PD-1** | **Parent PD-1** | **CD47 KO PD1** | | **CD47 KO PD1** | **CD47 KO PD1** | **CD47 KO PD1** | **CD47 KO PD1** | **CD47 KO PD1** | | **CD47 KO PD1** | **CD47 KO PD1** | **CD47 KO PD1** | **CD47 KO PD1** | **Parent Cas9** | **Parent Cas9** | | **Parent Cas9** | **Parent Cas9** | **Parent Cas9** | **Parent Cas9** | | **Parent Cas9** | **Parent Cas9** | **Parent Cas9** | **Parent Cas9** | **Parent** | | **Parent** | **Parent** | **Parent** | | **Parent** | **Parent** | **Parent** | | **Parent** | **Parent** | **Parent** |
| --- | --- | --- | --- | --- | --- | --- | --- | --- | --- | --- | --- | --- | --- | --- | --- | --- | --- | --- | --- | --- | --- | --- | --- | --- | --- | --- | --- | --- | --- | --- | --- | --- | --- | --- | --- | --- | --- | --- | --- | --- | --- | --- | --- | --- | --- | --- | --- | --- | --- | --- | --- | --- | --- | --- | --- | --- | --- | --- |
| **0** | 0.0000000 | 0.0000000 | 0.0000000 | 0.0000000 | 0.0000000 | 0.0000000 | 0.0000000 | 0.0000000 | 0.0000000 | 0.0000000 | 0.0000000 | 0.0000000 | 0.0000000 | 0.0000000 | 0.0000000 | 0.0000000 | 0.0000000 | | 0.0000000 | 0.0000000 | 0.0000000 | 0.0000000 | | 0.0000000 | 0.0000000 | 0.0000000 | 0.0000000 | 0.0000000 | | 0.0000000 | 0.0000000 | 0.0000000 | 0.0000000 | 0.0000000 | 0.0000000 | | 0.0000000 | 0.0000000 | 0.0000000 | 0.0000000 | | 0.0000000 | 0.0000000 | 0.0000000 | 0.0000000 | 0.0000000 | | 0.0000000 | 0.0000000 | 0.0000000 | | 0.0000000 | 0.0000000 | 0.0000000 | | 0.0000000 | 0.0000000 | 0.0000000 |
| **4** | 18.3750000 | 13.5000000 | 25.2700000 | 13.5000000 | 13.5000000 | 13.5000000 | 14.4150000 | 13.5000000 | 13.5000000 | 19.4400000 | 19.4560000 | 15.8720000 | 18.3750000 | 13.5000000 | 13.5000000 | 22.3820000 | 28.8990000 | | 18.3750000 | 18.3750000 | 13.5000000 | 14.4000000 | | 18.3750000 | 15.3600000 | 18.3750000 | 15.3600000 | 18.3750000 | | 19.6000000 | 18.3750000 | 21.9040000 | 25.2700000 | 19.6000000 | 23.9575000 | | 15.3600000 | 13.5000000 | 13.5000000 | 15.3600000 | | 28.0000000 | 18.3750000 | 18.3750000 | 20.5350000 | 15.7500000 | | 19.4400000 | 21.6600000 | 13.5000000 | | 13.5000000 | 21.6600000 | 18.3750000 | | 27.4360000 | 13.5000000 | 18.3750000 |
| **7** | 21.4375000 | 29.6000000 | 21.2195000 | 13.5000000 | 14.8955000 | 25.2700000 | 14.8955000 | 18.3750000 | 28.0000000 | 24.8000000 | 58.5225000 | 35.2800000 | 45.5625000 | 49.7025000 | 44.1800000 | 68.4000000 | 75.6250000 | | 48.0200000 | 75.6250000 | 48.0200000 | 14.4150000 | | 13.5000000 | 18.3750000 | 24.0000000 | 26.0555000 | 21.4375000 | | 19.6000000 | 13.5000000 | 14.4150000 | 21.9040000 | 48.0200000 | 60.0000000 | | 50.0000000 | 43.7500000 | 13.5000000 | 48.3840000 | | 28.0000000 | 45.5625000 | 40.5000000 | 46.0800000 | 54.0225000 | | 83.7225000 | 54.0800000 | 45.5625000 | | 31.3875000 | 51.2500000 | 40.5000000 | | 65.0250000 | 37.5000000 | 56.2500000 |
| **9** | 4.0000000 | 9.3750000 | 9.0000000 | 0.0000000 | 0.0000000 | 7.8125000 | 4.0000000 | 9.1125000 | 15.8720000 | 10.9760000 | 60.5000000 | 48.0200000 | 62.4240000 | 88.2000000 | 21.6600000 | 76.2805000 | 111.6300000 | | 69.6200000 | 56.2500000 | 24.8000000 | 6.2500000 | | 9.0000000 | 4.0000000 | 12.1500000 | 7.8125000 | 4.0000000 | | 5.0000000 | 7.8125000 | 4.2000000 | 11.2500000 | 40.5000000 | 72.0000000 | | 21.6600000 | 63.0000000 | 13.5000000 | 56.2500000 | | 49.2205000 | 60.0000000 | 68.0625000 | 72.1280000 | 108.0000000 | | 105.8750000 | 90.0000000 | 39.0150000 | | 32.0000000 | 58.5225000 | 60.0000000 | | 86.2400000 | 43.7500000 | 72.0000000 |
| **11** | 0.0000000 | 0.0000000 | 0.0000000 | 0.0000000 | 0.0000000 | 0.0000000 | 0.0000000 | 0.0000000 | 4.0000000 | 4.0000000 | 50.0000000 | 25.6000000 | 73.7500000 | 58.5225000 | 4.0000000 | 89.7800000 | 108.0000000 | | 43.7500000 | 56.2500000 | 25.3125000 | 0.0000000 | | 0.0000000 | 0.0000000 | 4.0000000 | 0.0000000 | 0.0000000 | | 1.0000000 | 0.0000000 | 0.0000000 | 0.5000000 | 32.0000000 | 68.0625000 | | 9.3750000 | 69.6200000 | 13.5000000 | 9.0000000 | | 7.8125000 | 46.0800000 | 33.6200000 | 56.2500000 | 106.2000000 | | 194.4000000 | 90.0000000 | 56.8620000 | | 28.0000000 | 62.7200000 | 90.7500000 | | 89.3475000 | 30.3750000 | 99.0000000 |
| **13** | 0.0000000 | 0.0000000 | 0.0000000 | 0.0000000 | 0.0000000 | 0.0000000 | 0.0000000 | 0.0000000 | 0.0000000 | 0.0000000 | 28.0000000 | 13.5000000 | 60.5000000 | 65.0250000 | 0.0000000 | 66.6000000 | 77.1375000 | | 13.5000000 | 28.0000000 | 0.0000000 | 0.0000000 | | 0.0000000 | 0.0000000 | 4.0000000 | 0.0000000 | 0.0000000 | | 0.0000000 | 0.0000000 | 0.0000000 | 0.0000000 | 4.0000000 | 28.0000000 | | 0.0000000 | 50.7195000 | 0.0000000 | 4.0000000 | | 4.0000000 | 40.5000000 | 56.2500000 | 62.5000000 | 91.8000000 | | 126.0000000 | 58.5225000 | 30.3750000 | | 29.6000000 | 72.0000000 | 87.0250000 | | 90.0000000 | 42.0175000 | 88.2000000 |
| **15** | 0.0000000 | 0.0000000 | 0.0000000 | 0.0000000 | 0.0000000 | 0.0000000 | 0.0000000 | 0.0000000 | 0.0000000 | 0.0000000 | 13.5000000 | 7.8125000 | 34.4605000 | 32.0000000 | 0.0000000 | 45.3750000 | 75.6250000 | | 0.0000000 | 6.2500000 | 0.0000000 | 0.0000000 | | 0.0000000 | 0.0000000 | 4.0000000 | 0.0000000 | 0.0000000 | | 0.0000000 | 0.0000000 | 0.0000000 | 0.0000000 | 0.0000000 | 13.5000000 | | 0.0000000 | 43.7500000 | 0.0000000 | 0.0000000 | | 0.0000000 | 35.4375000 | 45.5625000 | 61.2500000 | 91.8000000 | | 147.0000000 | 88.2000000 | 24.0000000 | | 21.4375000 | 73.8000000 | 108.0000000 | | 94.8855000 | 35.4375000 | 101.4000000 |
| **Within each row, compare columns (simple effects within rows)** | | | | | | | | | | | | | | | | | |  | | | | |  | | | | | |  | | | | | | |  | | | | |  | | | | | |  | | | |  | | | |  | | | |
|  | | | | | | | | | | | | | | | | | |  | | | | |  | | | | | |  | | | | | | |  | | | | |  | | | | | |  | | | |  | | | |  | | | |
| **Number of families** | | | | | | | | | | | | | | | | | | 7 | | | | |  | | | | | |  | | | | | | |  | | | | |  | | | | | |  | | | |  | | | |  | | | |
| **Number of comparisons per family** | | | | | | | | | | | | | | | | | | 6 | | | | |  | | | | | |  | | | | | | |  | | | | |  | | | | | |  | | | |  | | | |  | | | |
| **Alpha** | | | | | | | | | | | | | | | | | | 0.05 | | | | |  | | | | | |  | | | | | | |  | | | | |  | | | | | |  | | | |  | | | |  | | | |
|  | | | | | | | | | | | | | | | | | |  | | | | |  | | | | | |  | | | | | | |  | | | | |  | | | | | |  | | | |  | | | |  | | | |
| **Tukey's multiple comparisons test** | | | | | | | | | | | | | | | | | | Mean Diff. | | | | | 95.00% CI of diff. | | | | | | Below threshold? | | | | | | | Summary | | | | | Adjusted P Value | | | | | |  | | | |  | | | |  | | | |
|  | | | | | | | | | | | | | | | | | |  | | | | |  | | | | | |  | | | | | | |  | | | | |  | | | | | |  | | | |  | | | |  | | | |
| **Row 1** | | | | | | | | | | | | | | | | | |  | | | | |  | | | | | |  | | | | | | |  | | | | |  | | | | | |  | | | |  | | | |  | | | |
| **CD47 KO vs. Parent PD-1** | | | | | | | | | | | | | | | | | | 0.000 | | | | | -21.19 to 21.19 | | | | | | No | | | | | | | ns | | | | | >0.9999 | | | | | |  | | | |  | | | |  | | | |
| **CD47 KO vs. CD47 KO PD1** | | | | | | | | | | | | | | | | | | 0.000 | | | | | -21.19 to 21.19 | | | | | | No | | | | | | | ns | | | | | >0.9999 | | | | | |  | | | |  | | | |  | | | |
| **CD47 KO vs. Parent** | | | | | | | | | | | | | | | | | | 0.000 | | | | | -21.19 to 21.19 | | | | | | No | | | | | | | ns | | | | | >0.9999 | | | | | |  | | | |  | | | |  | | | |
| **Parent PD-1 vs. CD47 KO PD1** | | | | | | | | | | | | | | | | | | 0.000 | | | | | -21.19 to 21.19 | | | | | | No | | | | | | | ns | | | | | >0.9999 | | | | | |  | | | |  | | | |  | | | |
| **Parent PD-1 vs. Parent** | | | | | | | | | | | | | | | | | | 0.000 | | | | | -21.19 to 21.19 | | | | | | No | | | | | | | ns | | | | | >0.9999 | | | | | |  | | | |  | | | |  | | | |
| **CD47 KO PD1 vs. Parent** | | | | | | | | | | | | | | | | | | 0.000 | | | | | -21.19 to 21.19 | | | | | | No | | | | | | | ns | | | | | >0.9999 | | | | | |  | | | |  | | | |  | | | |
|  | | | | | | | | | | | | | | | | | |  | | | | |  | | | | | |  | | | | | | |  | | | | |  | | | | | |  | | | |  | | | |  | | | |
| **Row 2** | | | | | | | | | | | | | | | | | |  | | | | |  | | | | | |  | | | | | | |  | | | | |  | | | | | |  | | | |  | | | |  | | | |
| **CD47 KO vs. Parent PD-1** | | | | | | | | | | | | | | | | | | -2.373 | | | | | -23.57 to 18.82 | | | | | | No | | | | | | | ns | | | | | 0.9915 | | | | | |  | | | |  | | | |  | | | |
| **CD47 KO vs. CD47 KO PD1** | | | | | | | | | | | | | | | | | | -2.689 | | | | | -23.88 to 18.50 | | | | | | No | | | | | | | ns | | | | | 0.9878 | | | | | |  | | | |  | | | |  | | | |
| **CD47 KO vs. Parent** | | | | | | | | | | | | | | | | | | -2.470 | | | | | -23.66 to 18.72 | | | | | | No | | | | | | | ns | | | | | 0.9905 | | | | | |  | | | |  | | | |  | | | |
| **Parent PD-1 vs. CD47 KO PD1** | | | | | | | | | | | | | | | | | | -0.3160 | | | | | -21.51 to 20.88 | | | | | | No | | | | | | | ns | | | | | >0.9999 | | | | | |  | | | |  | | | |  | | | |
| **Parent PD-1 vs. Parent** | | | | | | | | | | | | | | | | | | -0.09620 | | | | | -21.29 to 21.10 | | | | | | No | | | | | | | ns | | | | | >0.9999 | | | | | |  | | | |  | | | |  | | | |
| **CD47 KO PD1 vs. Parent** | | | | | | | | | | | | | | | | | | 0.2198 | | | | | -20.97 to 21.41 | | | | | | No | | | | | | | ns | | | | | >0.9999 | | | | | |  | | | |  | | | |  | | | |
|  | | | | | | | | | | | | | | | | | |  | | | | |  | | | | | |  | | | | | | |  | | | | |  | | | | | |  | | | |  | | | |  | | | |
| **Row 3** | | | | | | | | | | | | | | | | | |  | | | | |  | | | | | |  | | | | | | |  | | | | |  | | | | | |  | | | |  | | | |  | | | |
| **CD47 KO vs. Parent PD-1** | | | | | | | | | | | | | | | | | | -33.69 | | | | | -54.89 to -12.50 | | | | | | Yes | | | | | | | *** | | | | | 0.0003 | | | | | |  | | | |  | | | |  | | | |
| **CD47 KO vs. CD47 KO PD1** | | | | | | | | | | | | | | | | | | 2.479 | | | | | -18.71 to 23.67 | | | | | | No | | | | | | | ns | | | | | 0.9904 | | | | | |  | | | |  | | | |  | | | |
| **CD47 KO vs. Parent** | | | | | | | | | | | | | | | | | | -30.73 | | | | | -51.92 to -9.537 | | | | | | Yes | | | | | | | ** | | | | | 0.0012 | | | | | |  | | | |  | | | |  | | | |
| **Parent PD-1 vs. CD47 KO PD1** | | | | | | | | | | | | | | | | | | 36.17 | | | | | 14.98 to 57.37 | | | | | | Yes | | | | | | | **** | | | | | <0.0001 | | | | | |  | | | |  | | | |  | | | |
| **Parent PD-1 vs. Parent** | | | | | | | | | | | | | | | | | | 2.964 | | | | | -18.23 to 24.16 | | | | | | No | | | | | | | ns | | | | | 0.9838 | | | | | |  | | | |  | | | |  | | | |
| **CD47 KO PD1 vs. Parent** | | | | | | | | | | | | | | | | | | -33.21 | | | | | -54.40 to -12.02 | | | | | | Yes | | | | | | | *** | | | | | 0.0004 | | | | | |  | | | |  | | | |  | | | |
|  | | | | | | | | | | | | | | | | | |  | | | | |  | | | | | |  | | | | | | |  | | | | |  | | | | | |  | | | |  | | | |  | | | |
| **Row 4** | | | | | | | | | | | | | | | | | |  | | | | |  | | | | | |  | | | | | | |  | | | | |  | | | | | |  | | | |  | | | |  | | | |
| **CD47 KO vs. Parent PD-1** | | | | | | | | | | | | | | | | | | -54.92 | | | | | -76.12 to -33.73 | | | | | | Yes | | | | | | | **** | | | | | <0.0001 | | | | | |  | | | |  | | | |  | | | |
| **CD47 KO vs. CD47 KO PD1** | | | | | | | | | | | | | | | | | | -0.1327 | | | | | -21.33 to 21.06 | | | | | | No | | | | | | | ns | | | | | >0.9999 | | | | | |  | | | |  | | | |  | | | |
| **CD47 KO vs. Parent** | | | | | | | | | | | | | | | | | | -62.53 | | | | | -83.72 to -41.33 | | | | | | Yes | | | | | | | **** | | | | | <0.0001 | | | | | |  | | | |  | | | |  | | | |
| **Parent PD-1 vs. CD47 KO PD1** | | | | | | | | | | | | | | | | | | 54.79 | | | | | 33.60 to 75.98 | | | | | | Yes | | | | | | | **** | | | | | <0.0001 | | | | | |  | | | |  | | | |  | | | |
| **Parent PD-1 vs. Parent** | | | | | | | | | | | | | | | | | | -7.602 | | | | | -28.80 to 13.59 | | | | | | No | | | | | | | ns | | | | | 0.7901 | | | | | |  | | | |  | | | |  | | | |
| **CD47 KO PD1 vs. Parent** | | | | | | | | | | | | | | | | | | -62.39 | | | | | -83.59 to -41.20 | | | | | | Yes | | | | | | | **** | | | | | <0.0001 | | | | | |  | | | |  | | | |  | | | |
|  | | | | | | | | | | | | | | | | | |  | | | | |  | | | | | |  | | | | | | |  | | | | |  | | | | | |  | | | |  | | | |  | | | |
| **Row 5** | | | | | | | | | | | | | | | | | |  | | | | |  | | | | | |  | | | | | | |  | | | | |  | | | | | |  | | | |  | | | |  | | | |
| **CD47 KO vs. Parent PD-1** | | | | | | | | | | | | | | | | | | -52.70 | | | | | -73.89 to -31.50 | | | | | | Yes | | | | | | | **** | | | | | <0.0001 | | | | | |  | | | |  | | | |  | | | |
| **CD47 KO vs. CD47 KO PD1** | | | | | | | | | | | | | | | | | | 0.2500 | | | | | -20.94 to 21.44 | | | | | | No | | | | | | | ns | | | | | >0.9999 | | | | | |  | | | |  | | | |  | | | |
| **CD47 KO vs. Parent** | | | | | | | | | | | | | | | | | | -83.97 | | | | | -105.2 to -62.77 | | | | | | Yes | | | | | | | **** | | | | | <0.0001 | | | | | |  | | | |  | | | |  | | | |
| **Parent PD-1 vs. CD47 KO PD1** | | | | | | | | | | | | | | | | | | 52.95 | | | | | 31.75 to 74.14 | | | | | | Yes | | | | | | | **** | | | | | <0.0001 | | | | | |  | | | |  | | | |  | | | |
| **Parent PD-1 vs. Parent** | | | | | | | | | | | | | | | | | | -31.27 | | | | | -52.46 to -10.08 | | | | | | Yes | | | | | | | *** | | | | | 0.0010 | | | | | |  | | | |  | | | |  | | | |
| **CD47 KO PD1 vs. Parent** | | | | | | | | | | | | | | | | | | -84.22 | | | | | -105.4 to -63.02 | | | | | | Yes | | | | | | | **** | | | | | <0.0001 | | | | | |  | | | |  | | | |  | | | |
|  | | | | | | | | | | | | | | | | | |  | | | | |  | | | | | |  | | | | | | |  | | | | |  | | | | | |  | | | |  | | | |  | | | |
| **Row 6** | | | | | | | | | | | | | | | | | |  | | | | |  | | | | | |  | | | | | | |  | | | | |  | | | | | |  | | | |  | | | |  | | | |
| **CD47 KO vs. Parent PD-1** | | | | | | | | | | | | | | | | | | -35.23 | | | | | -56.42 to -14.03 | | | | | | Yes | | | | | | | *** | | | | | 0.0001 | | | | | |  | | | |  | | | |  | | | |
| **CD47 KO vs. CD47 KO PD1** | | | | | | | | | | | | | | | | | | -0.4000 | | | | | -21.59 to 20.79 | | | | | | No | | | | | | | ns | | | | | >0.9999 | | | | | |  | | | |  | | | |  | | | |
| **CD47 KO vs. Parent** | | | | | | | | | | | | | | | | | | -71.55 | | | | | -92.75 to -50.36 | | | | | | Yes | | | | | | | **** | | | | | <0.0001 | | | | | |  | | | |  | | | |  | | | |
| **Parent PD-1 vs. CD47 KO PD1** | | | | | | | | | | | | | | | | | | 34.83 | | | | | 13.63 to 56.02 | | | | | | Yes | | | | | | | *** | | | | | 0.0002 | | | | | |  | | | |  | | | |  | | | |
| **Parent PD-1 vs. Parent** | | | | | | | | | | | | | | | | | | -36.33 | | | | | -57.52 to -15.13 | | | | | | Yes | | | | | | | **** | | | | | <0.0001 | | | | | |  | | | |  | | | |  | | | |
| **CD47 KO PD1 vs. Parent** | | | | | | | | | | | | | | | | | | -71.15 | | | | | -92.35 to -49.96 | | | | | | Yes | | | | | | | **** | | | | | <0.0001 | | | | | |  | | | |  | | | |  | | | |
|  | | | | | | | | | | | | | | | | | |  | | | | |  | | | | | |  | | | | | | |  | | | | |  | | | | | |  | | | |  | | | |  | | | |
| **Row 7** | | | | | | | | | | | | | | | | | |  | | | | |  | | | | | |  | | | | | | |  | | | | |  | | | | | |  | | | |  | | | |  | | | |
| **CD47 KO vs. Parent PD-1** | | | | | | | | | | | | | | | | | | -21.50 | | | | | -42.70 to -0.3089 | | | | | | Yes | | | | | | | * | | | | | 0.0453 | | | | | |  | | | |  | | | |  | | | |
| **CD47 KO vs. CD47 KO PD1** | | | | | | | | | | | | | | | | | | -0.4000 | | | | | -21.59 to 20.79 | | | | | | No | | | | | | | ns | | | | | >0.9999 | | | | | |  | | | |  | | | |  | | | |
| **CD47 KO vs. Parent** | | | | | | | | | | | | | | | | | | -78.60 | | | | | -99.79 to -57.40 | | | | | | Yes | | | | | | | **** | | | | | <0.0001 | | | | | |  | | | |  | | | |  | | | |
| **Parent PD-1 vs. CD47 KO PD1** | | | | | | | | | | | | | | | | | | 21.10 | | | | | -0.09109 to 42.30 | | | | | | No | | | | | | | ns | | | | | 0.0515 | | | | | |  | | | |  | | | |  | | | |
| **Parent PD-1 vs. Parent** | | | | | | | | | | | | | | | | | | -57.09 | | | | | -78.29 to -35.90 | | | | | | Yes | | | | | | | **** | | | | | <0.0001 | | | | | |  | | | |  | | | |  | | | |
| **CD47 KO PD1 vs. Parent** | | | | | | | | | | | | | | | | | | -78.20 | | | | | -99.39 to -57.00 | | | | | | Yes | | | | | | | **** | | | | | <0.0001 | | | | | |  | | | |  | | | |  | | | |
|  | | | | | | | | | | | | | | | | | |  | | | | |  | | | | | |  | | | | | | |  | | | | |  | | | | | |  | | | |  | | | |  | | | |
|  | | | | | | | | | | | | | | | | | |  | | | | |  | | | | | |  | | | | | | |  | | | | |  | | | | | |  | | | |  | | | |  | | | |
| **Test details** | | | | | | | | | | | | | | | | | | Mean 1 | | | | | Mean 2 | | | | | | Mean Diff. | | | | | | | SE of diff. | | | | | N1 | | | | | | N2 | | | | q | | | | DF | | | |
|  | | | | | | | | | | | | | | | | | |  | | | | |  | | | | | |  | | | | | | |  | | | | |  | | | | | |  | | | |  | | | |  | | | |
| **Row 1** | | | | | | | | | | | | | | | | | |  | | | | |  | | | | | |  | | | | | | |  | | | | |  | | | | | |  | | | |  | | | |  | | | |
| **CD47 KO vs. Parent PD-1** | | | | | | | | | | | | | | | | | | 0.000 | | | | | 0.000 | | | | | | 0.000 | | | | | | | 8.195 | | | | | 10 | | | | | | 10 | | | | 0.000 | | | | 252.0 | | | |
| **CD47 KO vs. CD47 KO PD1** | | | | | | | | | | | | | | | | | | 0.000 | | | | | 0.000 | | | | | | 0.000 | | | | | | | 8.195 | | | | | 10 | | | | | | 10 | | | | 0.000 | | | | 252.0 | | | |
| **CD47 KO vs. Parent** | | | | | | | | | | | | | | | | | | 0.000 | | | | | 0.000 | | | | | | 0.000 | | | | | | | 8.195 | | | | | 10 | | | | | | 10 | | | | 0.000 | | | | 252.0 | | | |
| **Parent PD-1 vs. CD47 KO PD1** | | | | | | | | | | | | | | | | | | 0.000 | | | | | 0.000 | | | | | | 0.000 | | | | | | | 8.195 | | | | | 10 | | | | | | 10 | | | | 0.000 | | | | 252.0 | | | |
| **Parent PD-1 vs. Parent** | | | | | | | | | | | | | | | | | | 0.000 | | | | | 0.000 | | | | | | 0.000 | | | | | | | 8.195 | | | | | 10 | | | | | | 10 | | | | 0.000 | | | | 252.0 | | | |
| **CD47 KO PD1 vs. Parent** | | | | | | | | | | | | | | | | | | 0.000 | | | | | 0.000 | | | | | | 0.000 | | | | | | | 8.195 | | | | | 10 | | | | | | 10 | | | | 0.000 | | | | 252.0 | | | |
|  | | | | | | | | | | | | | | | | | |  | | | | |  | | | | | |  | | | | | | |  | | | | |  | | | | | |  | | | |  | | | |  | | | |
| **Row 2** | | | | | | | | | | | | | | | | | |  | | | | |  | | | | | |  | | | | | | |  | | | | |  | | | | | |  | | | |  | | | |  | | | |
| **CD47 KO vs. Parent PD-1** | | | | | | | | | | | | | | | | | | 15.85 | | | | | 18.22 | | | | | | -2.373 | | | | | | | 8.195 | | | | | 10 | | | | | | 10 | | | | 0.4096 | | | | 252.0 | | | |
| **CD47 KO vs. CD47 KO PD1** | | | | | | | | | | | | | | | | | | 15.85 | | | | | 18.54 | | | | | | -2.689 | | | | | | | 8.195 | | | | | 10 | | | | | | 10 | | | | 0.4641 | | | | 252.0 | | | |
| **CD47 KO vs. Parent** | | | | | | | | | | | | | | | | | | 15.85 | | | | | 18.32 | | | | | | -2.470 | | | | | | | 8.195 | | | | | 10 | | | | | | 10 | | | | 0.4262 | | | | 252.0 | | | |
| **Parent PD-1 vs. CD47 KO PD1** | | | | | | | | | | | | | | | | | | 18.22 | | | | | 18.54 | | | | | | -0.3160 | | | | | | | 8.195 | | | | | 10 | | | | | | 10 | | | | 0.05453 | | | | 252.0 | | | |
| **Parent PD-1 vs. Parent** | | | | | | | | | | | | | | | | | | 18.22 | | | | | 18.32 | | | | | | -0.09620 | | | | | | | 8.195 | | | | | 10 | | | | | | 10 | | | | 0.01660 | | | | 252.0 | | | |
| **CD47 KO PD1 vs. Parent** | | | | | | | | | | | | | | | | | | 18.54 | | | | | 18.32 | | | | | | 0.2198 | | | | | | | 8.195 | | | | | 10 | | | | | | 10 | | | | 0.03793 | | | | 252.0 | | | |
|  | | | | | | | | | | | | | | | | | |  | | | | |  | | | | | |  | | | | | | |  | | | | |  | | | | | |  | | | |  | | | |  | | | |
| **Row 3** | | | | | | | | | | | | | | | | | |  | | | | |  | | | | | |  | | | | | | |  | | | | |  | | | | | |  | | | |  | | | |  | | | |
| **CD47 KO vs. Parent PD-1** | | | | | | | | | | | | | | | | | | 21.20 | | | | | 54.89 | | | | | | -33.69 | | | | | | | 8.195 | | | | | 10 | | | | | | 10 | | | | 5.815 | | | | 252.0 | | | |
| **CD47 KO vs. CD47 KO PD1** | | | | | | | | | | | | | | | | | | 21.20 | | | | | 18.72 | | | | | | 2.479 | | | | | | | 8.195 | | | | | 10 | | | | | | 10 | | | | 0.4278 | | | | 252.0 | | | |
| **CD47 KO vs. Parent** | | | | | | | | | | | | | | | | | | 21.20 | | | | | 51.93 | | | | | | -30.73 | | | | | | | 8.195 | | | | | 10 | | | | | | 10 | | | | 5.303 | | | | 252.0 | | | |
| **Parent PD-1 vs. CD47 KO PD1** | | | | | | | | | | | | | | | | | | 54.89 | | | | | 18.72 | | | | | | 36.17 | | | | | | | 8.195 | | | | | 10 | | | | | | 10 | | | | 6.243 | | | | 252.0 | | | |
| **Parent PD-1 vs. Parent** | | | | | | | | | | | | | | | | | | 54.89 | | | | | 51.93 | | | | | | 2.964 | | | | | | | 8.195 | | | | | 10 | | | | | | 10 | | | | 0.5115 | | | | 252.0 | | | |
| **CD47 KO PD1 vs. Parent** | | | | | | | | | | | | | | | | | | 18.72 | | | | | 51.93 | | | | | | -33.21 | | | | | | | 8.195 | | | | | 10 | | | | | | 10 | | | | 5.731 | | | | 252.0 | | | |
|  | | | | | | | | | | | | | | | | | |  | | | | |  | | | | | |  | | | | | | |  | | | | |  | | | | | |  | | | |  | | | |  | | | |
| **Row 4** | | | | | | | | | | | | | | | | | |  | | | | |  | | | | | |  | | | | | | |  | | | | |  | | | | | |  | | | |  | | | |  | | | |
| **CD47 KO vs. Parent PD-1** | | | | | | | | | | | | | | | | | | 7.015 | | | | | 61.94 | | | | | | -54.92 | | | | | | | 8.195 | | | | | 10 | | | | | | 10 | | | | 9.479 | | | | 252.0 | | | |
| **CD47 KO vs. CD47 KO PD1** | | | | | | | | | | | | | | | | | | 7.015 | | | | | 7.148 | | | | | | -0.1327 | | | | | | | 8.195 | | | | | 10 | | | | | | 10 | | | | 0.02290 | | | | 252.0 | | | |
| **CD47 KO vs. Parent** | | | | | | | | | | | | | | | | | | 7.015 | | | | | 69.54 | | | | | | -62.53 | | | | | | | 8.195 | | | | | 10 | | | | | | 10 | | | | 10.79 | | | | 252.0 | | | |
| **Parent PD-1 vs. CD47 KO PD1** | | | | | | | | | | | | | | | | | | 61.94 | | | | | 7.148 | | | | | | 54.79 | | | | | | | 8.195 | | | | | 10 | | | | | | 10 | | | | 9.456 | | | | 252.0 | | | |
| **Parent PD-1 vs. Parent** | | | | | | | | | | | | | | | | | | 61.94 | | | | | 69.54 | | | | | | -7.602 | | | | | | | 8.195 | | | | | 10 | | | | | | 10 | | | | 1.312 | | | | 252.0 | | | |
| **CD47 KO PD1 vs. Parent** | | | | | | | | | | | | | | | | | | 7.148 | | | | | 69.54 | | | | | | -62.39 | | | | | | | 8.195 | | | | | 10 | | | | | | 10 | | | | 10.77 | | | | 252.0 | | | |
|  | | | | | | | | | | | | | | | | | |  | | | | |  | | | | | |  | | | | | | |  | | | | |  | | | | | |  | | | |  | | | |  | | | |
| **Row 5** | | | | | | | | | | | | | | | | | |  | | | | |  | | | | | |  | | | | | | |  | | | | |  | | | | | |  | | | |  | | | |  | | | |
| **CD47 KO vs. Parent PD-1** | | | | | | | | | | | | | | | | | | 0.8000 | | | | | 53.50 | | | | | | -52.70 | | | | | | | 8.195 | | | | | 10 | | | | | | 10 | | | | 9.094 | | | | 252.0 | | | |
| **CD47 KO vs. CD47 KO PD1** | | | | | | | | | | | | | | | | | | 0.8000 | | | | | 0.5500 | | | | | | 0.2500 | | | | | | | 8.195 | | | | | 10 | | | | | | 10 | | | | 0.04314 | | | | 252.0 | | | |
| **CD47 KO vs. Parent** | | | | | | | | | | | | | | | | | | 0.8000 | | | | | 84.77 | | | | | | -83.97 | | | | | | | 8.195 | | | | | 10 | | | | | | 10 | | | | 14.49 | | | | 252.0 | | | |
| **Parent PD-1 vs. CD47 KO PD1** | | | | | | | | | | | | | | | | | | 53.50 | | | | | 0.5500 | | | | | | 52.95 | | | | | | | 8.195 | | | | | 10 | | | | | | 10 | | | | 9.137 | | | | 252.0 | | | |
| **Parent PD-1 vs. Parent** | | | | | | | | | | | | | | | | | | 53.50 | | | | | 84.77 | | | | | | -31.27 | | | | | | | 8.195 | | | | | 10 | | | | | | 10 | | | | 5.396 | | | | 252.0 | | | |
| **CD47 KO PD1 vs. Parent** | | | | | | | | | | | | | | | | | | 0.5500 | | | | | 84.77 | | | | | | -84.22 | | | | | | | 8.195 | | | | | 10 | | | | | | 10 | | | | 14.53 | | | | 252.0 | | | |
|  | | | | | | | | | | | | | | | | | |  | | | | |  | | | | | |  | | | | | | |  | | | | |  | | | | | |  | | | |  | | | |  | | | |
| **Row 6** | | | | | | | | | | | | | | | | | |  | | | | |  | | | | | |  | | | | | | |  | | | | |  | | | | | |  | | | |  | | | |  | | | |
| **CD47 KO vs. Parent PD-1** | | | | | | | | | | | | | | | | | | 0.000 | | | | | 35.23 | | | | | | -35.23 | | | | | | | 8.195 | | | | | 10 | | | | | | 10 | | | | 6.079 | | | | 252.0 | | | |
| **CD47 KO vs. CD47 KO PD1** | | | | | | | | | | | | | | | | | | 0.000 | | | | | 0.4000 | | | | | | -0.4000 | | | | | | | 8.195 | | | | | 10 | | | | | | 10 | | | | 0.06903 | | | | 252.0 | | | |
| **CD47 KO vs. Parent** | | | | | | | | | | | | | | | | | | 0.000 | | | | | 71.55 | | | | | | -71.55 | | | | | | | 8.195 | | | | | 10 | | | | | | 10 | | | | 12.35 | | | | 252.0 | | | |
| **Parent PD-1 vs. CD47 KO PD1** | | | | | | | | | | | | | | | | | | 35.23 | | | | | 0.4000 | | | | | | 34.83 | | | | | | | 8.195 | | | | | 10 | | | | | | 10 | | | | 6.010 | | | | 252.0 | | | |
| **Parent PD-1 vs. Parent** | | | | | | | | | | | | | | | | | | 35.23 | | | | | 71.55 | | | | | | -36.33 | | | | | | | 8.195 | | | | | 10 | | | | | | 10 | | | | 6.269 | | | | 252.0 | | | |
| **CD47 KO PD1 vs. Parent** | | | | | | | | | | | | | | | | | | 0.4000 | | | | | 71.55 | | | | | | -71.15 | | | | | | | 8.195 | | | | | 10 | | | | | | 10 | | | | 12.28 | | | | 252.0 | | | |
|  | | | | | | | | | | | | | | | | | |  | | | | |  | | | | | |  | | | | | | |  | | | | |  | | | | | |  | | | |  | | | |  | | | |
| **Row 7** | | | | | | | | | | | | | | | | | |  | | | | |  | | | | | |  | | | | | | |  | | | | |  | | | | | |  | | | |  | | | |  | | | |
| **CD47 KO vs. Parent PD-1** | | | | | | | | | | | | | | | | | | 0.000 | | | | | 21.50 | | | | | | -21.50 | | | | | | | 8.195 | | | | | 10 | | | | | | 10 | | | | 3.711 | | | | 252.0 | | | |
| **CD47 KO vs. CD47 KO PD1** | | | | | | | | | | | | | | | | | | 0.000 | | | | | 0.4000 | | | | | | -0.4000 | | | | | | | 8.195 | | | | | 10 | | | | | | 10 | | | | 0.06903 | | | | 252.0 | | | |
| **CD47 KO vs. Parent** | | | | | | | | | | | | | | | | | | 0.000 | | | | | 78.60 | | | | | | -78.60 | | | | | | | 8.195 | | | | | 10 | | | | | | 10 | | | | 13.56 | | | | 252.0 | | | |
| **Parent PD-1 vs. CD47 KO PD1** | | | | | | | | | | | | | | | | | | 21.50 | | | | | 0.4000 | | | | | | 21.10 | | | | | | | 8.195 | | | | | 10 | | | | | | 10 | | | | 3.642 | | | | 252.0 | | | |
| **Parent PD-1 vs. Parent** | | | | | | | | | | | | | | | | | | 21.50 | | | | | 78.60 | | | | | | -57.09 | | | | | | | 8.195 | | | | | 10 | | | | | | 10 | | | | 9.853 | | | | 252.0 | | | |
| **CD47 KO PD1 vs. Parent** | | | | | | | | | | | | | | | | | | 0.4000 | | | | | 78.60 | | | | | | -78.20 | | | | | | | 8.195 | | | | | 10 | | | | | | 10 | | | | 13.49 | | | | 252.0 | | | |

**Figure 1E**

|  | **4MOSC1** | **4MOSC1** | **4MOSC1** | **4MOSC1** | **4MOSC1** | **4MOSC1** | **4MOSC1** | **4MOSC1** | **4MOSC1** | **4MOSC1** | **4MOSC1 + Anti PD-1** | **4MOSC1 + Anti PD-1** | **4MOSC1 + Anti PD-1** | **4MOSC1 + Anti PD-1** | **4MOSC1 + Anti PD-1** | **4MOSC1 + Anti PD-1** | **4MOSC1 + Anti PD-1** | **4MOSC1 + Anti PD-1** | **4MOSC1 + Anti PD-1** | **4MOSC1 + Anti PD-1** | **4MOSC1 Cas9KO CD47KO** | **4MOSC1 Cas9KO CD47KO** | **4MOSC1 Cas9KO CD47KO** | **4MOSC1 Cas9KO CD47KO** | **4MOSC1 Cas9KO CD47KO** | **4MOSC1 Cas9KO CD47KO** | **4MOSC1 Cas9KO CD47KO** | **4MOSC1 Cas9KO CD47KO** | **4MOSC1 Cas9KO CD47KO** | **4MOSC1 Cas9KO CD47KO** | **4MOSC1 Cas9KO CD47KO + Anti PD-1** | **4MOSC1 Cas9KO CD47KO + Anti PD-1** | **4MOSC1 Cas9KO CD47KO + Anti PD-1** | **4MOSC1 Cas9KO CD47KO + Anti PD-1** | **4MOSC1 Cas9KO CD47KO + Anti PD-1** | **4MOSC1 Cas9KO CD47KO + Anti PD-1** | **4MOSC1 Cas9KO CD47KO + Anti PD-1** | **4MOSC1 Cas9KO CD47KO + Anti PD-1** | **4MOSC1 Cas9KO CD47KO + Anti PD-1** | **4MOSC1 Cas9KO CD47KO + Anti PD-1** |
| --- | --- | --- | --- | --- | --- | --- | --- | --- | --- | --- | --- | --- | --- | --- | --- | --- | --- | --- | --- | --- | --- | --- | --- | --- | --- | --- | --- | --- | --- | --- | --- | --- | --- | --- | --- | --- | --- | --- | --- | --- |
| **0** | 0.0000 | 0.0000 | 0.0000 | 0.0000 | 0.000 | 0.0000 | 0.0000 | 0.0000 | 0.0000 | 0.0000 | 0.0000 | 0.0000 | 0.000 | 0.000 | 0.0000 | 0.0000 | 0.00 | 0.000 | 0.0000 | 0.0000 | 0.0000 | 0.0000 | 0.0000 | 0.0000 | 0.0000 | 0.0000 | 0.0000 | 0.0000 | 0.000 | 0.000 | 0.0000 | 0.0000 | 0.0000 | 0.0000 | 0.0000 | 0.0000 | 0.0000 | 0.000 | 0.0000 | 0.0000 |
| **4** | 13.5000 | 11.2500 | 15.7500 | 15.7500 | 15.750 | 15.7500 | 15.7500 | 13.5000 | 15.7500 | 15.7500 | 15.7500 | 13.5000 | 9.375 | 13.500 | 15.7500 | 7.8125 | 13.50 | 13.500 | 15.7500 | 9.3750 | 7.8125 | 9.3750 | 9.3750 | 6.0000 | 13.5000 | 13.5000 | 9.3750 | 9.3750 | 13.500 | 13.500 | 13.5000 | 9.3750 | 9.3750 | 9.3750 | 9.3750 | 13.5000 | 13.5000 | 9.375 | 9.3750 | 7.8125 |
| **6** | 24.5000 | 40.0000 | 36.0000 | 36.0000 | 32.000 | 36.0000 | 28.0000 | 32.0000 | 27.5625 | 32.0000 | 45.5625 | 32.0000 | 32.000 | 56.250 | 36.0000 | 15.7500 | 32.00 | 40.500 | 50.6250 | 32.0000 | 28.0000 | 18.3750 | 15.7500 | 21.4375 | 9.3750 | 32.0000 | 21.4375 | 21.4375 | 24.500 | 32.000 | 32.0000 | 24.5000 | 13.5000 | 21.4375 | 40.5000 | 40.5000 | 32.0000 | 24.500 | 18.0000 | 15.7500 |
| **8** | 36.0000 | 50.6250 | 50.6250 | 50.6250 | 56.250 | 45.5625 | 50.0000 | 95.0625 | 44.0000 | 95.0625 | 68.0625 | 45.5625 | 28.000 | 90.000 | 50.0000 | 24.5000 | 56.25 | 50.000 | 45.5625 | 62.5000 | 32.0000 | 32.0000 | 18.0000 | 24.5000 | 28.0000 | 40.5000 | 40.5000 | 40.5000 | 24.500 | 40.500 | 50.0000 | 15.7500 | 15.7500 | 30.3750 | 35.4375 | 50.0000 | 56.2500 | 18.000 | 24.5000 | 32.0000 |
| **10** | 50.6250 | 62.5000 | 45.5625 | 45.5625 | 75.625 | 68.7500 | 72.0000 | 122.5000 | 44.0000 | 110.2500 | 75.6250 | 36.0000 | 4.000 | 60.500 | 56.2500 | 32.0000 | 56.25 | 62.500 | 27.5625 | 75.6250 | 21.4375 | 32.0000 | 15.7500 | 15.7500 | 13.5000 | 68.0625 | 40.5000 | 56.2500 | 18.000 | 24.500 | 28.0000 | 15.7500 | 13.5000 | 9.3750 | 28.0000 | 28.0000 | 50.0000 | 9.375 | 24.5000 | 24.5000 |
| **12** | 62.5000 | 83.1875 | 32.0000 | 62.5000 | 75.625 | 75.0000 | 60.5000 | 144.0000 | 52.0000 | 225.0000 | 56.2500 | 45.5625 | 0.000 | 75.625 | 40.5000 | 15.7500 | 75.00 | 75.625 | 9.3750 | 99.0000 | 13.5000 | 21.4375 | 15.7500 | 7.8125 | 7.8125 | 50.0000 | 32.0000 | 32.0000 | 9.375 | 24.500 | 7.8125 | 7.8125 | 7.8125 | 0.0000 | 9.3750 | 7.8125 | 10.9375 | 4.000 | 10.9375 | 21.4375 |
| **14** | 68.7500 | 68.7500 | 24.5000 | 56.2500 | 99.000 | 55.6875 | 75.6250 | 158.4375 | 44.0000 | 208.0000 | 56.2500 | 28.0000 | 0.000 | 75.625 | 21.4375 | 15.7500 | 68.75 | 68.750 | 7.8125 | 147.0000 | 9.3750 | 15.7500 | 9.3750 | 9.3750 | 0.0000 | 40.5000 | 9.3750 | 24.0000 | 9.375 | 9.375 | 0.0000 | 5.0000 | 4.0000 | 0.0000 | 5.0000 | 0.0000 | 9.3750 | 0.000 | 9.3750 | 15.7500 |
| **16** | 81.2500 | 83.1875 | 15.7500 | 45.5625 | 159.250 | 126.7500 | 62.5000 | 135.0000 | 44.0000 | 240.0000 | 56.2500 | 24.5000 | 0.000 | 56.250 | 0.0000 | 15.7500 | 117.00 | 62.500 | 0.0000 | 171.5000 | 7.8125 | 9.3750 | 7.8125 | 0.0000 | 0.0000 | 40.5000 | 7.8125 | 28.0000 | 0.000 | 0.000 | 0.0000 | 0.0000 | 0.0000 | 0.0000 | 0.0000 | 0.0000 | 0.0000 | 0.000 | 0.0000 | 13.5000 |
| **18** | 98.3125 | 75.6250 | 15.7500 | 56.2500 | 183.750 | 87.5000 | 56.2500 | 98.3125 | 44.0000 | 270.9375 | 50.0000 | 18.0000 | 0.000 | 62.500 | 0.0000 | 13.5000 | 144.00 | 56.250 | 0.0000 | 210.9375 | 0.0000 | 5.0000 | 4.0000 | 0.0000 | 0.0000 | 21.4375 | 4.0000 | 20.0000 | 0.000 | 0.000 | 0.0000 | 0.0000 | 0.0000 | 0.0000 | 0.0000 | 0.0000 | 0.0000 | 0.000 | 0.0000 | 13.5000 |
| **20** | 98.3125 | 108.0000 | 0.0000 | 56.2500 | 208.250 | 144.0000 | 68.0625 | 118.8000 | 48.0000 | 283.5000 | 108.0000 | 15.7500 | 0.000 | 90.000 | 0.0000 | 18.0000 | 153.00 | 68.750 | 0.0000 | 352.0000 | 0.0000 | 4.0000 | 4.0000 | 0.0000 | 0.0000 | 28.0000 | 0.0000 | 15.3125 | 0.000 | 0.000 | 0.0000 | 0.0000 | 0.0000 | 0.0000 | 0.0000 | 0.0000 | 0.0000 | 0.000 | 0.0000 | 9.3750 |

| **Within each row, compare columns (simple effects within rows)** |  |  |  |  |  |  |  |  |
| --- | --- | --- | --- | --- | --- | --- | --- | --- |
|  |  |  |  |  |  |  |  |  |
| **Number of families** | 10 |  |  |  |  |  |  |  |
| **Number of comparisons per family** | 6 |  |  |  |  |  |  |  |
| **Alpha** | 0.05 |  |  |  |  |  |  |  |
|  |  |  |  |  |  |  |  |  |
| **Tukey's multiple comparisons test** | Mean Diff. | 95.00% CI of diff. | Below threshold? | Summary | Adjusted P Value |  |  |  |
|  |  |  |  |  |  |  |  |  |
| **Row 1** |  |  |  |  |  |  |  |  |
| **4MOSC1 vs. 4MOSC1 + Anti PD-1** | 0.000 | -40.97 to 40.97 | No | ns | >0.9999 |  |  |  |
| **4MOSC1 vs. 4MOSC1 Cas9KO CD47KO** | 0.000 | -40.97 to 40.97 | No | ns | >0.9999 |  |  |  |
| **4MOSC1 vs. Group D** | 0.000 | -40.97 to 40.97 | No | ns | >0.9999 |  |  |  |
| **4MOSC1 + Anti PD-1 vs. 4MOSC1 Cas9KO CD47KO** | 0.000 | -40.97 to 40.97 | No | ns | >0.9999 |  |  |  |
| **4MOSC1 + Anti PD-1 vs. Group D** | 0.000 | -40.97 to 40.97 | No | ns | >0.9999 |  |  |  |
| **4MOSC1 Cas9KO CD47KO vs. Group D** | 0.000 | -40.97 to 40.97 | No | ns | >0.9999 |  |  |  |
|  |  |  |  |  |  |  |  |  |
| **Row 2** |  |  |  |  |  |  |  |  |
| **4MOSC1 vs. 4MOSC1 + Anti PD-1** | 2.069 | -38.90 to 43.04 | No | ns | 0.9992 |  |  |  |
| **4MOSC1 vs. 4MOSC1 Cas9KO CD47KO** | 4.319 | -36.65 to 45.29 | No | ns | 0.9930 |  |  |  |
| **4MOSC1 vs. Group D** | 4.394 | -36.58 to 45.36 | No | ns | 0.9926 |  |  |  |
| **4MOSC1 + Anti PD-1 vs. 4MOSC1 Cas9KO CD47KO** | 2.250 | -38.72 to 43.22 | No | ns | 0.9990 |  |  |  |
| **4MOSC1 + Anti PD-1 vs. Group D** | 2.325 | -38.65 to 43.30 | No | ns | 0.9989 |  |  |  |
| **4MOSC1 Cas9KO CD47KO vs. Group D** | 0.07500 | -40.90 to 41.05 | No | ns | >0.9999 |  |  |  |
|  |  |  |  |  |  |  |  |  |
| **Row 3** |  |  |  |  |  |  |  |  |
| **4MOSC1 vs. 4MOSC1 + Anti PD-1** | -4.863 | -45.83 to 36.11 | No | ns | 0.9900 |  |  |  |
| **4MOSC1 vs. 4MOSC1 Cas9KO CD47KO** | 9.975 | -31.00 to 50.95 | No | ns | 0.9229 |  |  |  |
| **4MOSC1 vs. Group D** | 6.138 | -34.83 to 47.11 | No | ns | 0.9803 |  |  |  |
| **4MOSC1 + Anti PD-1 vs. 4MOSC1 Cas9KO CD47KO** | 14.84 | -26.13 to 55.81 | No | ns | 0.7862 |  |  |  |
| **4MOSC1 + Anti PD-1 vs. Group D** | 11.00 | -29.97 to 51.97 | No | ns | 0.8997 |  |  |  |
| **4MOSC1 Cas9KO CD47KO vs. Group D** | -3.838 | -44.81 to 37.13 | No | ns | 0.9950 |  |  |  |
|  |  |  |  |  |  |  |  |  |
| **Row 4** |  |  |  |  |  |  |  |  |
| **4MOSC1 vs. 4MOSC1 + Anti PD-1** | 5.338 | -35.63 to 46.31 | No | ns | 0.9869 |  |  |  |
| **4MOSC1 vs. 4MOSC1 Cas9KO CD47KO** | 25.28 | -15.69 to 66.25 | No | ns | 0.3841 |  |  |  |
| **4MOSC1 vs. Group D** | 24.58 | -16.40 to 65.55 | No | ns | 0.4099 |  |  |  |
| **4MOSC1 + Anti PD-1 vs. 4MOSC1 Cas9KO CD47KO** | 19.94 | -21.03 to 60.91 | No | ns | 0.5913 |  |  |  |
| **4MOSC1 + Anti PD-1 vs. Group D** | 19.24 | -21.73 to 60.21 | No | ns | 0.6197 |  |  |  |
| **4MOSC1 Cas9KO CD47KO vs. Group D** | -0.7062 | -41.68 to 40.26 | No | ns | >0.9999 |  |  |  |
|  |  |  |  |  |  |  |  |  |
| **Row 5** |  |  |  |  |  |  |  |  |
| **4MOSC1 vs. 4MOSC1 + Anti PD-1** | 21.11 | -19.86 to 62.08 | No | ns | 0.5446 |  |  |  |
| **4MOSC1 vs. 4MOSC1 Cas9KO CD47KO** | 39.16 | -1.808 to 80.13 | No | ns | 0.0669 |  |  |  |
| **4MOSC1 vs. Group D** | 46.64 | 5.667 to 87.61 | Yes | * | 0.0184 |  |  |  |
| **4MOSC1 + Anti PD-1 vs. 4MOSC1 Cas9KO CD47KO** | 18.06 | -22.91 to 59.03 | No | ns | 0.6666 |  |  |  |
| **4MOSC1 + Anti PD-1 vs. Group D** | 25.53 | -15.44 to 66.50 | No | ns | 0.3751 |  |  |  |
| **4MOSC1 Cas9KO CD47KO vs. Group D** | 7.475 | -33.50 to 48.45 | No | ns | 0.9654 |  |  |  |
|  |  |  |  |  |  |  |  |  |
| **Row 6** |  |  |  |  |  |  |  |  |
| **4MOSC1 vs. 4MOSC1 + Anti PD-1** | 37.96 | -3.008 to 78.93 | No | ns | 0.0805 |  |  |  |
| **4MOSC1 vs. 4MOSC1 Cas9KO CD47KO** | 65.81 | 24.84 to 106.8 | Yes | *** | 0.0002 |  |  |  |
| **4MOSC1 vs. Group D** | 78.44 | 37.47 to 119.4 | Yes | **** | <0.0001 |  |  |  |
| **4MOSC1 + Anti PD-1 vs. 4MOSC1 Cas9KO CD47KO** | 27.85 | -13.12 to 68.82 | No | ns | 0.2972 |  |  |  |
| **4MOSC1 + Anti PD-1 vs. Group D** | 40.48 | -0.4958 to 81.45 | No | ns | 0.0542 |  |  |  |
| **4MOSC1 Cas9KO CD47KO vs. Group D** | 12.63 | -28.35 to 53.60 | No | ns | 0.8565 |  |  |  |
|  |  |  |  |  |  |  |  |  |
| **Row 7** |  |  |  |  |  |  |  |  |
| **4MOSC1 vs. 4MOSC1 + Anti PD-1** | 36.96 | -4.008 to 77.93 | No | ns | 0.0935 |  |  |  |
| **4MOSC1 vs. 4MOSC1 Cas9KO CD47KO** | 72.25 | 31.28 to 113.2 | Yes | **** | <0.0001 |  |  |  |
| **4MOSC1 vs. Group D** | 81.05 | 40.08 to 122.0 | Yes | **** | <0.0001 |  |  |  |
| **4MOSC1 + Anti PD-1 vs. 4MOSC1 Cas9KO CD47KO** | 35.29 | -5.683 to 76.26 | No | ns | 0.1190 |  |  |  |
| **4MOSC1 + Anti PD-1 vs. Group D** | 44.09 | 3.117 to 85.06 | Yes | * | 0.0293 |  |  |  |
| **4MOSC1 Cas9KO CD47KO vs. Group D** | 8.800 | -32.17 to 49.77 | No | ns | 0.9453 |  |  |  |
|  |  |  |  |  |  |  |  |  |
| **Row 8** |  |  |  |  |  |  |  |  |
| **4MOSC1 vs. 4MOSC1 + Anti PD-1** | 48.95 | 7.979 to 89.92 | Yes | * | 0.0118 |  |  |  |
| **4MOSC1 vs. 4MOSC1 Cas9KO CD47KO** | 89.19 | 48.22 to 130.2 | Yes | **** | <0.0001 |  |  |  |
| **4MOSC1 vs. Group D** | 97.98 | 57.00 to 138.9 | Yes | **** | <0.0001 |  |  |  |
| **4MOSC1 + Anti PD-1 vs. 4MOSC1 Cas9KO CD47KO** | 40.24 | -0.7271 to 81.21 | No | ns | 0.0563 |  |  |  |
| **4MOSC1 + Anti PD-1 vs. Group D** | 49.03 | 8.054 to 90.00 | Yes | * | 0.0116 |  |  |  |
| **4MOSC1 Cas9KO CD47KO vs. Group D** | 8.781 | -32.19 to 49.75 | No | ns | 0.9456 |  |  |  |
|  |  |  |  |  |  |  |  |  |
| **Row 9** |  |  |  |  |  |  |  |  |
| **4MOSC1 vs. 4MOSC1 + Anti PD-1** | 43.15 | 2.179 to 84.12 | Yes | * | 0.0346 |  |  |  |
| **4MOSC1 vs. 4MOSC1 Cas9KO CD47KO** | 93.23 | 52.25 to 134.2 | Yes | **** | <0.0001 |  |  |  |
| **4MOSC1 vs. Group D** | 97.32 | 56.35 to 138.3 | Yes | **** | <0.0001 |  |  |  |
| **4MOSC1 + Anti PD-1 vs. 4MOSC1 Cas9KO CD47KO** | 50.08 | 9.104 to 91.05 | Yes | ** | 0.0094 |  |  |  |
| **4MOSC1 + Anti PD-1 vs. Group D** | 54.17 | 13.20 to 95.14 | Yes | ** | 0.0040 |  |  |  |
| **4MOSC1 Cas9KO CD47KO vs. Group D** | 4.094 | -36.88 to 45.06 | No | ns | 0.9940 |  |  |  |
|  |  |  |  |  |  |  |  |  |
| **Row 10** |  |  |  |  |  |  |  |  |
| **4MOSC1 vs. 4MOSC1 + Anti PD-1** | 32.77 | -8.203 to 73.74 | No | ns | 0.1669 |  |  |  |
| **4MOSC1 vs. 4MOSC1 Cas9KO CD47KO** | 108.2 | 67.22 to 149.2 | Yes | **** | <0.0001 |  |  |  |
| **4MOSC1 vs. Group D** | 112.4 | 71.41 to 153.4 | Yes | **** | <0.0001 |  |  |  |
| **4MOSC1 + Anti PD-1 vs. 4MOSC1 Cas9KO CD47KO** | 75.42 | 34.45 to 116.4 | Yes | **** | <0.0001 |  |  |  |
| **4MOSC1 + Anti PD-1 vs. Group D** | 79.61 | 38.64 to 120.6 | Yes | **** | <0.0001 |  |  |  |
| **4MOSC1 Cas9KO CD47KO vs. Group D** | 4.194 | -36.78 to 45.16 | No | ns | 0.9935 |  |  |  |
|  |  |  |  |  |  |  |  |  |
|  |  |  |  |  |  |  |  |  |
| **Test details** | Mean 1 | Mean 2 | Mean Diff. | SE of diff. | N1 | N2 | q | DF |
|  |  |  |  |  |  |  |  |  |
| **Row 1** |  |  |  |  |  |  |  |  |
| **4MOSC1 vs. 4MOSC1 + Anti PD-1** | 0.000 | 0.000 | 0.000 | 15.87 | 10 | 10 | 0.000 | 360.0 |
| **4MOSC1 vs. 4MOSC1 Cas9KO CD47KO** | 0.000 | 0.000 | 0.000 | 15.87 | 10 | 10 | 0.000 | 360.0 |
| **4MOSC1 vs. Group D** | 0.000 | 0.000 | 0.000 | 15.87 | 10 | 10 | 0.000 | 360.0 |
| **4MOSC1 + Anti PD-1 vs. 4MOSC1 Cas9KO CD47KO** | 0.000 | 0.000 | 0.000 | 15.87 | 10 | 10 | 0.000 | 360.0 |
| **4MOSC1 + Anti PD-1 vs. Group D** | 0.000 | 0.000 | 0.000 | 15.87 | 10 | 10 | 0.000 | 360.0 |
| **4MOSC1 Cas9KO CD47KO vs. Group D** | 0.000 | 0.000 | 0.000 | 15.87 | 10 | 10 | 0.000 | 360.0 |
|  |  |  |  |  |  |  |  |  |
| **Row 2** |  |  |  |  |  |  |  |  |
| **4MOSC1 vs. 4MOSC1 + Anti PD-1** | 14.85 | 12.78 | 2.069 | 15.87 | 10 | 10 | 0.1843 | 360.0 |
| **4MOSC1 vs. 4MOSC1 Cas9KO CD47KO** | 14.85 | 10.53 | 4.319 | 15.87 | 10 | 10 | 0.3848 | 360.0 |
| **4MOSC1 vs. Group D** | 14.85 | 10.46 | 4.394 | 15.87 | 10 | 10 | 0.3914 | 360.0 |
| **4MOSC1 + Anti PD-1 vs. 4MOSC1 Cas9KO CD47KO** | 12.78 | 10.53 | 2.250 | 15.87 | 10 | 10 | 0.2005 | 360.0 |
| **4MOSC1 + Anti PD-1 vs. Group D** | 12.78 | 10.46 | 2.325 | 15.87 | 10 | 10 | 0.2071 | 360.0 |
| **4MOSC1 Cas9KO CD47KO vs. Group D** | 10.53 | 10.46 | 0.07500 | 15.87 | 10 | 10 | 0.006682 | 360.0 |
|  |  |  |  |  |  |  |  |  |
| **Row 3** |  |  |  |  |  |  |  |  |
| **4MOSC1 vs. 4MOSC1 + Anti PD-1** | 32.41 | 37.27 | -4.863 | 15.87 | 10 | 10 | 0.4332 | 360.0 |
| **4MOSC1 vs. 4MOSC1 Cas9KO CD47KO** | 32.41 | 22.43 | 9.975 | 15.87 | 10 | 10 | 0.8887 | 360.0 |
| **4MOSC1 vs. Group D** | 32.41 | 26.27 | 6.138 | 15.87 | 10 | 10 | 0.5468 | 360.0 |
| **4MOSC1 + Anti PD-1 vs. 4MOSC1 Cas9KO CD47KO** | 37.27 | 22.43 | 14.84 | 15.87 | 10 | 10 | 1.322 | 360.0 |
| **4MOSC1 + Anti PD-1 vs. Group D** | 37.27 | 26.27 | 11.00 | 15.87 | 10 | 10 | 0.9800 | 360.0 |
| **4MOSC1 Cas9KO CD47KO vs. Group D** | 22.43 | 26.27 | -3.838 | 15.87 | 10 | 10 | 0.3419 | 360.0 |
|  |  |  |  |  |  |  |  |  |
| **Row 4** |  |  |  |  |  |  |  |  |
| **4MOSC1 vs. 4MOSC1 + Anti PD-1** | 57.38 | 52.04 | 5.338 | 15.87 | 10 | 10 | 0.4755 | 360.0 |
| **4MOSC1 vs. 4MOSC1 Cas9KO CD47KO** | 57.38 | 32.10 | 25.28 | 15.87 | 10 | 10 | 2.252 | 360.0 |
| **4MOSC1 vs. Group D** | 57.38 | 32.81 | 24.58 | 15.87 | 10 | 10 | 2.189 | 360.0 |
| **4MOSC1 + Anti PD-1 vs. 4MOSC1 Cas9KO CD47KO** | 52.04 | 32.10 | 19.94 | 15.87 | 10 | 10 | 1.777 | 360.0 |
| **4MOSC1 + Anti PD-1 vs. Group D** | 52.04 | 32.81 | 19.24 | 15.87 | 10 | 10 | 1.714 | 360.0 |
| **4MOSC1 Cas9KO CD47KO vs. Group D** | 32.10 | 32.81 | -0.7062 | 15.87 | 10 | 10 | 0.06292 | 360.0 |
|  |  |  |  |  |  |  |  |  |
| **Row 5** |  |  |  |  |  |  |  |  |
| **4MOSC1 vs. 4MOSC1 + Anti PD-1** | 69.74 | 48.63 | 21.11 | 15.87 | 10 | 10 | 1.880 | 360.0 |
| **4MOSC1 vs. 4MOSC1 Cas9KO CD47KO** | 69.74 | 30.58 | 39.16 | 15.87 | 10 | 10 | 3.489 | 360.0 |
| **4MOSC1 vs. Group D** | 69.74 | 23.10 | 46.64 | 15.87 | 10 | 10 | 4.155 | 360.0 |
| **4MOSC1 + Anti PD-1 vs. 4MOSC1 Cas9KO CD47KO** | 48.63 | 30.58 | 18.06 | 15.87 | 10 | 10 | 1.609 | 360.0 |
| **4MOSC1 + Anti PD-1 vs. Group D** | 48.63 | 23.10 | 25.53 | 15.87 | 10 | 10 | 2.275 | 360.0 |
| **4MOSC1 Cas9KO CD47KO vs. Group D** | 30.58 | 23.10 | 7.475 | 15.87 | 10 | 10 | 0.6660 | 360.0 |
|  |  |  |  |  |  |  |  |  |
| **Row 6** |  |  |  |  |  |  |  |  |
| **4MOSC1 vs. 4MOSC1 + Anti PD-1** | 87.23 | 49.27 | 37.96 | 15.87 | 10 | 10 | 3.382 | 360.0 |
| **4MOSC1 vs. 4MOSC1 Cas9KO CD47KO** | 87.23 | 21.42 | 65.81 | 15.87 | 10 | 10 | 5.863 | 360.0 |
| **4MOSC1 vs. Group D** | 87.23 | 8.794 | 78.44 | 15.87 | 10 | 10 | 6.988 | 360.0 |
| **4MOSC1 + Anti PD-1 vs. 4MOSC1 Cas9KO CD47KO** | 49.27 | 21.42 | 27.85 | 15.87 | 10 | 10 | 2.481 | 360.0 |
| **4MOSC1 + Anti PD-1 vs. Group D** | 49.27 | 8.794 | 40.48 | 15.87 | 10 | 10 | 3.606 | 360.0 |
| **4MOSC1 Cas9KO CD47KO vs. Group D** | 21.42 | 8.794 | 12.63 | 15.87 | 10 | 10 | 1.125 | 360.0 |
|  |  |  |  |  |  |  |  |  |
| **Row 7** |  |  |  |  |  |  |  |  |
| **4MOSC1 vs. 4MOSC1 + Anti PD-1** | 85.90 | 48.94 | 36.96 | 15.87 | 10 | 10 | 3.293 | 360.0 |
| **4MOSC1 vs. 4MOSC1 Cas9KO CD47KO** | 85.90 | 13.65 | 72.25 | 15.87 | 10 | 10 | 6.437 | 360.0 |
| **4MOSC1 vs. Group D** | 85.90 | 4.850 | 81.05 | 15.87 | 10 | 10 | 7.221 | 360.0 |
| **4MOSC1 + Anti PD-1 vs. 4MOSC1 Cas9KO CD47KO** | 48.94 | 13.65 | 35.29 | 15.87 | 10 | 10 | 3.144 | 360.0 |
| **4MOSC1 + Anti PD-1 vs. Group D** | 48.94 | 4.850 | 44.09 | 15.87 | 10 | 10 | 3.928 | 360.0 |
| **4MOSC1 Cas9KO CD47KO vs. Group D** | 13.65 | 4.850 | 8.800 | 15.87 | 10 | 10 | 0.7840 | 360.0 |
|  |  |  |  |  |  |  |  |  |
| **Row 8** |  |  |  |  |  |  |  |  |
| **4MOSC1 vs. 4MOSC1 + Anti PD-1** | 99.33 | 50.38 | 48.95 | 15.87 | 10 | 10 | 4.361 | 360.0 |
| **4MOSC1 vs. 4MOSC1 Cas9KO CD47KO** | 99.33 | 10.13 | 89.19 | 15.87 | 10 | 10 | 7.946 | 360.0 |
| **4MOSC1 vs. Group D** | 99.33 | 1.350 | 97.98 | 15.87 | 10 | 10 | 8.729 | 360.0 |
| **4MOSC1 + Anti PD-1 vs. 4MOSC1 Cas9KO CD47KO** | 50.38 | 10.13 | 40.24 | 15.87 | 10 | 10 | 3.585 | 360.0 |
| **4MOSC1 + Anti PD-1 vs. Group D** | 50.38 | 1.350 | 49.03 | 15.87 | 10 | 10 | 4.368 | 360.0 |
| **4MOSC1 Cas9KO CD47KO vs. Group D** | 10.13 | 1.350 | 8.781 | 15.87 | 10 | 10 | 0.7823 | 360.0 |
|  |  |  |  |  |  |  |  |  |
| **Row 9** |  |  |  |  |  |  |  |  |
| **4MOSC1 vs. 4MOSC1 + Anti PD-1** | 98.67 | 55.52 | 43.15 | 15.87 | 10 | 10 | 3.844 | 360.0 |
| **4MOSC1 vs. 4MOSC1 Cas9KO CD47KO** | 98.67 | 5.444 | 93.23 | 15.87 | 10 | 10 | 8.306 | 360.0 |
| **4MOSC1 vs. Group D** | 98.67 | 1.350 | 97.32 | 15.87 | 10 | 10 | 8.670 | 360.0 |
| **4MOSC1 + Anti PD-1 vs. 4MOSC1 Cas9KO CD47KO** | 55.52 | 5.444 | 50.08 | 15.87 | 10 | 10 | 4.461 | 360.0 |
| **4MOSC1 + Anti PD-1 vs. Group D** | 55.52 | 1.350 | 54.17 | 15.87 | 10 | 10 | 4.826 | 360.0 |
| **4MOSC1 Cas9KO CD47KO vs. Group D** | 5.444 | 1.350 | 4.094 | 15.87 | 10 | 10 | 0.3647 | 360.0 |
|  |  |  |  |  |  |  |  |  |
| **Row 10** |  |  |  |  |  |  |  |  |
| **4MOSC1 vs. 4MOSC1 + Anti PD-1** | 113.3 | 80.55 | 32.77 | 15.87 | 10 | 10 | 2.919 | 360.0 |
| **4MOSC1 vs. 4MOSC1 Cas9KO CD47KO** | 113.3 | 5.131 | 108.2 | 15.87 | 10 | 10 | 9.639 | 360.0 |
| **4MOSC1 vs. Group D** | 113.3 | 0.9375 | 112.4 | 15.87 | 10 | 10 | 10.01 | 360.0 |
| **4MOSC1 + Anti PD-1 vs. 4MOSC1 Cas9KO CD47KO** | 80.55 | 5.131 | 75.42 | 15.87 | 10 | 10 | 6.719 | 360.0 |
| **4MOSC1 + Anti PD-1 vs. Group D** | 80.55 | 0.9375 | 79.61 | 15.87 | 10 | 10 | 7.093 | 360.0 |
| **4MOSC1 Cas9KO CD47KO vs. Group D** | 5.131 | 0.9375 | 4.194 | 15.87 | 10 | 10 | 0.3736 | 360.0 |

**Figure 2A**

| **Days Post Transplant** | **Control** | **Control** | **Control** | **Control** | **Control** | **anti-PD1** | **anti-PD1** | **anti-PD1** | **anti-PD1** | **anti-PD1** | **anti-CD47** | **anti-CD47** | **anti-CD47** | **anti-CD47** | **anti-CD47** | **anti-PD1 + anti-CD47** | **anti-PD1 + anti-CD47** | **anti-PD1 + anti-CD47** | **anti-PD1 + anti-CD47** | **anti-PD1 + anti-CD47** |
| --- | --- | --- | --- | --- | --- | --- | --- | --- | --- | --- | --- | --- | --- | --- | --- | --- | --- | --- | --- | --- |
| **0** | 0.000 | 0.000 | 0.0000 | 0.0000 | 0.0000 | 0.0000 | 0.000 | 0.0000 | 0.0000 | 0.000 | 0.0000 | 0 | 0 | 0 | 0 | 0.0000 | 0.00 | 0.000 | 0.0000 | 0.000 |
| **4** | 9.800 | 11.250 | 9.1125 | 7.8125 | 5.2900 | 9.1125 | 11.700 | 7.8125 | 6.8750 | 13.500 | 11.2500 | 11 | 14 | 4 | 7 | 14.4150 | 15.36 | 12.150 | 7.8125 | 11.250 |
| **6** | 78.400 | 38.720 | 46.5520 | 37.9045 | 32.7795 | 35.2800 | 48.384 | 43.7760 | 55.2960 | 33.620 | 78.6500 | 59 | 29 | 34 | 27 | 37.9045 | 61.25 | 35.280 | 52.9920 | 68.992 |
| **9** | 109.770 | 75.625 | 70.3040 | 85.7820 | 98.6065 | 37.9045 | 117.242 | 32.0000 | 54.0225 | 100.800 | 95.8740 | 51 | 72 | 63 | 44 | 85.7820 | 92.51 | 68.952 | 86.0985 | 75.625 |
| **12** | 105.710 | 90.000 | 99.0000 | 106.2000 | 92.5100 | 28.0000 | 97.200 | 35.2800 | 35.4375 | 143.344 | 82.8495 | 15 | 51 | 48 | 45 | 46.0800 | 44.18 | 24.800 | 28.0000 | 30.400 |
| **16** | 154.733 | 164.150 | 149.4500 | 134.0960 | 99.0000 | 20.2125 | 81.675 | 30.4000 | 11.2500 | 210.938 | 34.4605 | 12 | 37 | 33 | 90 | 0.5000 | 19.60 | 13.500 | 2.0000 | 4.000 |
| **19** | 155.520 | 155.520 | 168.7500 | 182.5200 | 126.7500 | 0.0000 | 46.250 | 13.5000 | 0.0000* | 270.750 | 4.0000 | 0 | 14 | 8 | 28 | 0.0000 | 11.25 | 13.500 | 0.0000 | 0.000 |

| **Within each row, compare columns (simple effects within rows)** |  |  |  |  |  |  |  |  |
| --- | --- | --- | --- | --- | --- | --- | --- | --- |
|  |  |  |  |  |  |  |  |  |
| **Number of families** | 7 |  |  |  |  |  |  |  |
| **Number of comparisons per family** | 6 |  |  |  |  |  |  |  |
| **Alpha** | 0.05 |  |  |  |  |  |  |  |
|  |  |  |  |  |  |  |  |  |
| **Tukey's multiple comparisons test** | Predicted (LS) mean diff. | 95.00% CI of diff. | Below threshold? | Summary | Adjusted P Value |  |  |  |
|  |  |  |  |  |  |  |  |  |
| **Row 1** |  |  |  |  |  |  |  |  |
| **Control vs. anti-PD1** | -7.105E-15 | -52.23 to 52.23 | No | ns | >0.9999 |  |  |  |
| **Control vs. anti-CD47** | 2.842E-14 | -52.23 to 52.23 | No | ns | >0.9999 |  |  |  |
| **Control vs. anti-PD1 + anti-CD47** | -4.263E-14 | -52.23 to 52.23 | No | ns | >0.9999 |  |  |  |
| **anti-PD1 vs. anti-CD47** | 3.553E-14 | -52.23 to 52.23 | No | ns | >0.9999 |  |  |  |
| **anti-PD1 vs. anti-PD1 + anti-CD47** | -3.553E-14 | -52.23 to 52.23 | No | ns | >0.9999 |  |  |  |
| **anti-CD47 vs. anti-PD1 + anti-CD47** | -7.105E-14 | -52.23 to 52.23 | No | ns | >0.9999 |  |  |  |
|  |  |  |  |  |  |  |  |  |
| **Row 2** |  |  |  |  |  |  |  |  |
| **Control vs. anti-PD1** | -1.147 | -53.37 to 51.08 | No | ns | >0.9999 |  |  |  |
| **Control vs. anti-CD47** | -0.7220 | -52.95 to 51.50 | No | ns | >0.9999 |  |  |  |
| **Control vs. anti-PD1 + anti-CD47** | -3.544 | -55.77 to 48.68 | No | ns | 0.9980 |  |  |  |
| **anti-PD1 vs. anti-CD47** | 0.4250 | -51.80 to 52.65 | No | ns | >0.9999 |  |  |  |
| **anti-PD1 vs. anti-PD1 + anti-CD47** | -2.398 | -54.62 to 49.83 | No | ns | 0.9994 |  |  |  |
| **anti-CD47 vs. anti-PD1 + anti-CD47** | -2.823 | -55.05 to 49.40 | No | ns | 0.9990 |  |  |  |
|  |  |  |  |  |  |  |  |  |
| **Row 3** |  |  |  |  |  |  |  |  |
| **Control vs. anti-PD1** | 3.600 | -48.63 to 55.83 | No | ns | 0.9979 |  |  |  |
| **Control vs. anti-CD47** | 1.297 | -50.93 to 53.52 | No | ns | >0.9999 |  |  |  |
| **Control vs. anti-PD1 + anti-CD47** | -4.413 | -56.64 to 47.81 | No | ns | 0.9962 |  |  |  |
| **anti-PD1 vs. anti-CD47** | -2.303 | -54.53 to 49.92 | No | ns | 0.9995 |  |  |  |
| **anti-PD1 vs. anti-PD1 + anti-CD47** | -8.013 | -60.24 to 44.21 | No | ns | 0.9782 |  |  |  |
| **anti-CD47 vs. anti-PD1 + anti-CD47** | -5.709 | -57.93 to 46.52 | No | ns | 0.9919 |  |  |  |
|  |  |  |  |  |  |  |  |  |
| **Row 4** |  |  |  |  |  |  |  |  |
| **Control vs. anti-PD1** | 19.62 | -32.60 to 71.85 | No | ns | 0.7611 |  |  |  |
| **Control vs. anti-CD47** | 22.99 | -29.23 to 75.22 | No | ns | 0.6605 |  |  |  |
| **Control vs. anti-PD1 + anti-CD47** | 6.224 | -46.00 to 58.45 | No | ns | 0.9895 |  |  |  |
| **anti-PD1 vs. anti-CD47** | 3.368 | -48.86 to 55.59 | No | ns | 0.9983 |  |  |  |
| **anti-PD1 vs. anti-PD1 + anti-CD47** | -13.40 | -65.62 to 38.83 | No | ns | 0.9085 |  |  |  |
| **anti-CD47 vs. anti-PD1 + anti-CD47** | -16.77 | -68.99 to 35.46 | No | ns | 0.8365 |  |  |  |
|  |  |  |  |  |  |  |  |  |
| **Row 5** |  |  |  |  |  |  |  |  |
| **Control vs. anti-PD1** | 30.83 | -21.39 to 83.06 | No | ns | 0.4174 |  |  |  |
| **Control vs. anti-CD47** | 50.22 | -2.001 to 102.4 | No | ns | 0.0641 |  |  |  |
| **Control vs. anti-PD1 + anti-CD47** | 63.99 | 11.77 to 116.2 | Yes | ** | 0.0097 |  |  |  |
| **anti-PD1 vs. anti-CD47** | 19.39 | -32.83 to 71.62 | No | ns | 0.7676 |  |  |  |
| **anti-PD1 vs. anti-PD1 + anti-CD47** | 33.16 | -19.06 to 85.39 | No | ns | 0.3518 |  |  |  |
| **anti-CD47 vs. anti-PD1 + anti-CD47** | 13.77 | -38.46 to 65.99 | No | ns | 0.9016 |  |  |  |
|  |  |  |  |  |  |  |  |  |
| **Row 6** |  |  |  |  |  |  |  |  |
| **Control vs. anti-PD1** | 69.39 | 17.17 to 121.6 | Yes | ** | 0.0041 |  |  |  |
| **Control vs. anti-CD47** | 99.13 | 46.90 to 151.4 | Yes | **** | <0.0001 |  |  |  |
| **Control vs. anti-PD1 + anti-CD47** | 132.4 | 80.14 to 184.6 | Yes | **** | <0.0001 |  |  |  |
| **anti-PD1 vs. anti-CD47** | 29.74 | -22.49 to 81.96 | No | ns | 0.4498 |  |  |  |
| **anti-PD1 vs. anti-PD1 + anti-CD47** | 62.98 | 10.75 to 115.2 | Yes | * | 0.0113 |  |  |  |
| **anti-CD47 vs. anti-PD1 + anti-CD47** | 33.24 | -18.99 to 85.46 | No | ns | 0.3498 |  |  |  |
|  |  |  |  |  |  |  |  |  |
| **Row 7** |  |  |  |  |  |  |  |  |
| **Control vs. anti-PD1** | 75.19 | 19.79 to 130.6 | Yes | ** | 0.0032 |  |  |  |
| **Control vs. anti-CD47** | 147.1 | 94.92 to 199.4 | Yes | **** | <0.0001 |  |  |  |
| **Control vs. anti-PD1 + anti-CD47** | 152.9 | 100.6 to 205.1 | Yes | **** | <0.0001 |  |  |  |
| **anti-PD1 vs. anti-CD47** | 71.96 | 16.57 to 127.4 | Yes | ** | 0.0053 |  |  |  |
| **anti-PD1 vs. anti-PD1 + anti-CD47** | 77.68 | 22.28 to 133.1 | Yes | ** | 0.0022 |  |  |  |
| **anti-CD47 vs. anti-PD1 + anti-CD47** | 5.713 | -46.51 to 57.94 | No | ns | 0.9919 |  |  |  |
|  |  |  |  |  |  |  |  |  |
|  |  |  |  |  |  |  |  |  |
| **Test details** | Predicted (LS) mean 1 | Predicted (LS) mean 2 | Predicted (LS) mean diff. | SE of diff. | N1 | N2 | q | DF |
|  |  |  |  |  |  |  |  |  |
| **Row 1** |  |  |  |  |  |  |  |  |
| **Control vs. anti-PD1** | 2.842E-14 | 3.553E-14 | -7.105E-15 | 20.02 | 5 | 5 | 5.019E-16 | 111.0 |
| **Control vs. anti-CD47** | 2.842E-14 | 0.000 | 2.842E-14 | 20.02 | 5 | 5 | 2.008E-15 | 111.0 |
| **Control vs. anti-PD1 + anti-CD47** | 2.842E-14 | 7.105E-14 | -4.263E-14 | 20.02 | 5 | 5 | 3.011E-15 | 111.0 |
| **anti-PD1 vs. anti-CD47** | 3.553E-14 | 0.000 | 3.553E-14 | 20.02 | 5 | 5 | 2.509E-15 | 111.0 |
| **anti-PD1 vs. anti-PD1 + anti-CD47** | 3.553E-14 | 7.105E-14 | -3.553E-14 | 20.02 | 5 | 5 | 2.509E-15 | 111.0 |
| **anti-CD47 vs. anti-PD1 + anti-CD47** | 0.000 | 7.105E-14 | -7.105E-14 | 20.02 | 5 | 5 | 5.019E-15 | 111.0 |
|  |  |  |  |  |  |  |  |  |
| **Row 2** |  |  |  |  |  |  |  |  |
| **Control vs. anti-PD1** | 8.653 | 9.800 | -1.147 | 20.02 | 5 | 5 | 0.08102 | 111.0 |
| **Control vs. anti-CD47** | 8.653 | 9.375 | -0.7220 | 20.02 | 5 | 5 | 0.05100 | 111.0 |
| **Control vs. anti-PD1 + anti-CD47** | 8.653 | 12.20 | -3.544 | 20.02 | 5 | 5 | 0.2504 | 111.0 |
| **anti-PD1 vs. anti-CD47** | 9.800 | 9.375 | 0.4250 | 20.02 | 5 | 5 | 0.03002 | 111.0 |
| **anti-PD1 vs. anti-PD1 + anti-CD47** | 9.800 | 12.20 | -2.398 | 20.02 | 5 | 5 | 0.1693 | 111.0 |
| **anti-CD47 vs. anti-PD1 + anti-CD47** | 9.375 | 12.20 | -2.823 | 20.02 | 5 | 5 | 0.1994 | 111.0 |
|  |  |  |  |  |  |  |  |  |
| **Row 3** |  |  |  |  |  |  |  |  |
| **Control vs. anti-PD1** | 46.87 | 43.27 | 3.600 | 20.02 | 5 | 5 | 0.2543 | 111.0 |
| **Control vs. anti-CD47** | 46.87 | 45.57 | 1.297 | 20.02 | 5 | 5 | 0.09159 | 111.0 |
| **Control vs. anti-PD1 + anti-CD47** | 46.87 | 51.28 | -4.413 | 20.02 | 5 | 5 | 0.3117 | 111.0 |
| **anti-PD1 vs. anti-CD47** | 43.27 | 45.57 | -2.303 | 20.02 | 5 | 5 | 0.1627 | 111.0 |
| **anti-PD1 vs. anti-PD1 + anti-CD47** | 43.27 | 51.28 | -8.013 | 20.02 | 5 | 5 | 0.5659 | 111.0 |
| **anti-CD47 vs. anti-PD1 + anti-CD47** | 45.57 | 51.28 | -5.709 | 20.02 | 5 | 5 | 0.4033 | 111.0 |
|  |  |  |  |  |  |  |  |  |
| **Row 4** |  |  |  |  |  |  |  |  |
| **Control vs. anti-PD1** | 88.02 | 68.39 | 19.62 | 20.02 | 5 | 5 | 1.386 | 111.0 |
| **Control vs. anti-CD47** | 88.02 | 65.03 | 22.99 | 20.02 | 5 | 5 | 1.624 | 111.0 |
| **Control vs. anti-PD1 + anti-CD47** | 88.02 | 81.79 | 6.224 | 20.02 | 5 | 5 | 0.4396 | 111.0 |
| **anti-PD1 vs. anti-CD47** | 68.39 | 65.03 | 3.368 | 20.02 | 5 | 5 | 0.2379 | 111.0 |
| **anti-PD1 vs. anti-PD1 + anti-CD47** | 68.39 | 81.79 | -13.40 | 20.02 | 5 | 5 | 0.9465 | 111.0 |
| **anti-CD47 vs. anti-PD1 + anti-CD47** | 65.03 | 81.79 | -16.77 | 20.02 | 5 | 5 | 1.184 | 111.0 |
|  |  |  |  |  |  |  |  |  |
| **Row 5** |  |  |  |  |  |  |  |  |
| **Control vs. anti-PD1** | 98.68 | 67.85 | 30.83 | 20.02 | 5 | 5 | 2.178 | 111.0 |
| **Control vs. anti-CD47** | 98.68 | 48.46 | 50.22 | 20.02 | 5 | 5 | 3.547 | 111.0 |
| **Control vs. anti-PD1 + anti-CD47** | 98.68 | 34.69 | 63.99 | 20.02 | 5 | 5 | 4.520 | 111.0 |
| **anti-PD1 vs. anti-CD47** | 67.85 | 48.46 | 19.39 | 20.02 | 5 | 5 | 1.370 | 111.0 |
| **anti-PD1 vs. anti-PD1 + anti-CD47** | 67.85 | 34.69 | 33.16 | 20.02 | 5 | 5 | 2.342 | 111.0 |
| **anti-CD47 vs. anti-PD1 + anti-CD47** | 48.46 | 34.69 | 13.77 | 20.02 | 5 | 5 | 0.9725 | 111.0 |
|  |  |  |  |  |  |  |  |  |
| **Row 6** |  |  |  |  |  |  |  |  |
| **Control vs. anti-PD1** | 140.3 | 70.90 | 69.39 | 20.02 | 5 | 5 | 4.901 | 111.0 |
| **Control vs. anti-CD47** | 140.3 | 41.16 | 99.13 | 20.02 | 5 | 5 | 7.002 | 111.0 |
| **Control vs. anti-PD1 + anti-CD47** | 140.3 | 7.920 | 132.4 | 20.02 | 5 | 5 | 9.349 | 111.0 |
| **anti-PD1 vs. anti-CD47** | 70.90 | 41.16 | 29.74 | 20.02 | 5 | 5 | 2.101 | 111.0 |
| **anti-PD1 vs. anti-PD1 + anti-CD47** | 70.90 | 7.920 | 62.98 | 20.02 | 5 | 5 | 4.448 | 111.0 |
| **anti-CD47 vs. anti-PD1 + anti-CD47** | 41.16 | 7.920 | 33.24 | 20.02 | 5 | 5 | 2.348 | 111.0 |
|  |  |  |  |  |  |  |  |  |
| **Row 7** |  |  |  |  |  |  |  |  |
| **Control vs. anti-PD1** | 157.8 | 82.63 | 75.19 | 21.24 | 5 | 4 | 5.007 | 111.0 |
| **Control vs. anti-CD47** | 157.8 | 10.66 | 147.1 | 20.02 | 5 | 5 | 10.39 | 111.0 |
| **Control vs. anti-PD1 + anti-CD47** | 157.8 | 4.950 | 152.9 | 20.02 | 5 | 5 | 10.80 | 111.0 |
| **anti-PD1 vs. anti-CD47** | 82.63 | 10.66 | 71.96 | 21.24 | 4 | 5 | 4.792 | 111.0 |
| **anti-PD1 vs. anti-PD1 + anti-CD47** | 82.63 | 4.950 | 77.68 | 21.24 | 4 | 5 | 5.173 | 111.0 |
| **anti-CD47 vs. anti-PD1 + anti-CD47** | 10.66 | 4.950 | 5.713 | 20.02 | 5 | 5 | 0.4035 | 111.0 |

**Figure 2B**

| **Days Post Transplant** | **Control** | **Control** | **Control** | **Control** | **Control** | **Control** | | **anti-PD1** | **anti-PD1** | **anti-PD1** | **anti-PD1** | | **anti-PD1** | **anti-PD1** | **anti-CD47** | **anti-CD47** | **anti-CD47** | | **anti-CD47** | **anti-CD47** | | **anti-CD47** | **anti-PD1 + anti-CD47** | **anti-PD1 + anti-CD47** | | **anti-PD1 + anti-CD47** | | **anti-PD1 + anti-CD47** | **anti-PD1 + anti-CD47** | | **anti-PD1 + anti-CD47** |
| --- | --- | --- | --- | --- | --- | --- | --- | --- | --- | --- | --- | --- | --- | --- | --- | --- | --- | --- | --- | --- | --- | --- | --- | --- | --- | --- | --- | --- | --- | --- | --- |
| **0** | 0.0000 | 0.0000 | 0.0000 | 0.0000 | 0.000 |  | | 0.0000 | 0.0000 | 0.000 | 0.0000 | | 0.00 |  | 0.000 | 0.0000 | 0.0000 | | 0.00 | 0.0000 | | 0.000 | 0.000 | 0.0000 | | 0.0000 | | 0.0000 | 0.0000 | | 0.0000 |
| **4** | 7.8125 | 7.8125 | 7.8125 | 4.0000 | 4.000 |  | | 13.5000 | 7.8125 | 11.250 | 7.8125 | | 4.00 |  | 4.000 | 7.8125 | 7.8125 | | 11.25 | 11.2500 | | 6.250 | 11.250 | 13.5000 | | 6.2500 | | 13.5000 | 7.8125 | | 11.2500 |
| **6** | 15.8720 | 18.4960 | 18.9875 | 10.5125 | 13.500 |  | | 17.4240 | 13.5000 | 12.800 | 27.3780 | | 13.50 |  | 11.250 | 16.3840 | 19.6000 | | 15.36 | 26.6175 | | 17.340 | 20.736 | 17.9200 | | 19.6000 | | 26.6175 | 18.3750 | | 21.4375 |
| **8** | 38.0250 | 28.8800 | 30.3240 | 32.0000 | 23.040 |  | | 40.0000 | 38.4000 | 16.384 | 40.0000 | | 46.08 |  | 31.941 | 32.4900 | 29.6000 | | 36.00 | 36.8000 | | 30.420 | 33.600 | 37.8225 | | 34.2225 | | 50.6250 | 27.8640 | | 39.2000 |
| **10** | 121.8350 | 73.7500 | 83.7500 | 75.0000 | 87.880 |  | | 55.6875 | 41.6000 | 4.000 | 84.5325 | | 84.27 |  | 72.030 | 81.6750 | 75.0000 | | 75.00 | 87.0790 | | 83.824 | 75.000 | 63.7500 | | 81.2500 | | 27.5625 | 66.0275 | | 90.7500 |
| **12** | 90.7500 | 75.0000 | 113.4380 | 87.5000 | 86.250 |  | | 50.6250 | 30.6250 | 0.000 | 126.0000 | | 44.00 |  | 40.000 | 55.6875 | 62.5000 | | 129.60 | 77.5000 | | 81.250 | 38.266 | 15.7500 | | 40.0000 | | 17.9200 | 42.8655 | | 66.2700 |
| **14** | 119.0700 | 81.6750 | 172.4940 | 243.3990 | 138.720 |  | | 45.5625 | 118.7840 | 0.000 | 40.5000 | | 46.08 |  | 32.000 | 108.0000 | 35.2800 | | 78.65 | 42.5250 | | 66.248 | 67.600 | 13.5000 | | 32.0000 | | 15.3600 | 58.5225 | | 45.5625 |
| **19** | 0.0000* | 171.5000 | 237.2760 | 197.7300 | 246.038 |  | | 32.0000 | 62.5000 | 0.000 | 0.0000* | | 13.50 |  | 0.000* | 5.8190 | 57.6000 | | 4.00 | 14.4150 | | 24.000 | 27.436 | 0.5000 | | 4.0000 | | 3.1250 | 38.8290 | | 62.5000 |
| **Within each row, compare columns (simple effects within rows)** | | | | | | |  | | | | |  | | |  | | |  | | |  | | | |  | |  | | |  | |
|  | | | | | | |  | | | | |  | | |  | | |  | | |  | | | |  | |  | | |  | |
| **Number of families** | | | | | | | 8 | | | | |  | | |  | | |  | | |  | | | |  | |  | | |  | |
| **Number of comparisons per family** | | | | | | | 6 | | | | |  | | |  | | |  | | |  | | | |  | |  | | |  | |
| **Alpha** | | | | | | | 0.05 | | | | |  | | |  | | |  | | |  | | | |  | |  | | |  | |
|  | | | | | | |  | | | | |  | | |  | | |  | | |  | | | |  | |  | | |  | |
| **Tukey's multiple comparisons test** | | | | | | | Predicted (LS) mean diff. | | | | | 95.00% CI of diff. | | | Below threshold? | | | Summary | | | Adjusted P Value | | | |  | |  | | |  | |
|  | | | | | | |  | | | | |  | | |  | | |  | | |  | | | |  | |  | | |  | |
| **Row 1** | | | | | | |  | | | | |  | | |  | | |  | | |  | | | |  | |  | | |  | |
| **Control vs. anti-PD1** | | | | | | | -7.105E-15 | | | | | -35.62 to 35.62 | | | No | | | ns | | | >0.9999 | | | |  | |  | | |  | |
| **Control vs. anti-CD47** | | | | | | | -2.842E-14 | | | | | -34.10 to 34.10 | | | No | | | ns | | | >0.9999 | | | |  | |  | | |  | |
| **Control vs. anti-PD1 + anti-CD47** | | | | | | | -2.842E-14 | | | | | -34.10 to 34.10 | | | No | | | ns | | | >0.9999 | | | |  | |  | | |  | |
| **anti-PD1 vs. anti-CD47** | | | | | | | -2.132E-14 | | | | | -34.10 to 34.10 | | | No | | | ns | | | >0.9999 | | | |  | |  | | |  | |
| **anti-PD1 vs. anti-PD1 + anti-CD47** | | | | | | | -2.132E-14 | | | | | -34.10 to 34.10 | | | No | | | ns | | | >0.9999 | | | |  | |  | | |  | |
| **anti-CD47 vs. anti-PD1 + anti-CD47** | | | | | | | 0.000 | | | | | -32.52 to 32.52 | | | No | | | ns | | | >0.9999 | | | |  | |  | | |  | |
|  | | | | | | |  | | | | |  | | |  | | |  | | |  | | | |  | |  | | |  | |
| **Row 2** | | | | | | |  | | | | |  | | |  | | |  | | |  | | | |  | |  | | |  | |
| **Control vs. anti-PD1** | | | | | | | -2.587 | | | | | -38.21 to 33.03 | | | No | | | ns | | | 0.9976 | | | |  | |  | | |  | |
| **Control vs. anti-CD47** | | | | | | | -1.775 | | | | | -35.88 to 32.33 | | | No | | | ns | | | 0.9991 | | | |  | |  | | |  | |
| **Control vs. anti-PD1 + anti-CD47** | | | | | | | -4.306 | | | | | -38.41 to 29.80 | | | No | | | ns | | | 0.9877 | | | |  | |  | | |  | |
| **anti-PD1 vs. anti-CD47** | | | | | | | 0.8125 | | | | | -33.29 to 34.92 | | | No | | | ns | | | >0.9999 | | | |  | |  | | |  | |
| **anti-PD1 vs. anti-PD1 + anti-CD47** | | | | | | | -1.719 | | | | | -35.82 to 32.38 | | | No | | | ns | | | 0.9992 | | | |  | |  | | |  | |
| **anti-CD47 vs. anti-PD1 + anti-CD47** | | | | | | | -2.531 | | | | | -35.05 to 29.98 | | | No | | | ns | | | 0.9971 | | | |  | |  | | |  | |
|  | | | | | | |  | | | | |  | | |  | | |  | | |  | | | |  | |  | | |  | |
| **Row 3** | | | | | | |  | | | | |  | | |  | | |  | | |  | | | |  | |  | | |  | |
| **Control vs. anti-PD1** | | | | | | | -1.447 | | | | | -37.07 to 34.17 | | | No | | | ns | | | 0.9996 | | | |  | |  | | |  | |
| **Control vs. anti-CD47** | | | | | | | -2.285 | | | | | -36.39 to 31.82 | | | No | | | ns | | | 0.9981 | | | |  | |  | | |  | |
| **Control vs. anti-PD1 + anti-CD47** | | | | | | | -5.307 | | | | | -39.41 to 28.80 | | | No | | | ns | | | 0.9775 | | | |  | |  | | |  | |
| **anti-PD1 vs. anti-CD47** | | | | | | | -0.8382 | | | | | -34.94 to 33.26 | | | No | | | ns | | | >0.9999 | | | |  | |  | | |  | |
| **anti-PD1 vs. anti-PD1 + anti-CD47** | | | | | | | -3.861 | | | | | -37.96 to 30.24 | | | No | | | ns | | | 0.9911 | | | |  | |  | | |  | |
| **anti-CD47 vs. anti-PD1 + anti-CD47** | | | | | | | -3.022 | | | | | -35.54 to 29.49 | | | No | | | ns | | | 0.9950 | | | |  | |  | | |  | |
|  | | | | | | |  | | | | |  | | |  | | |  | | |  | | | |  | |  | | |  | |
| **Row 4** | | | | | | |  | | | | |  | | |  | | |  | | |  | | | |  | |  | | |  | |
| **Control vs. anti-PD1** | | | | | | | -5.719 | | | | | -41.34 to 29.90 | | | No | | | ns | | | 0.9754 | | | |  | |  | | |  | |
| **Control vs. anti-CD47** | | | | | | | -2.421 | | | | | -36.52 to 31.68 | | | No | | | ns | | | 0.9978 | | | |  | |  | | |  | |
| **Control vs. anti-PD1 + anti-CD47** | | | | | | | -6.769 | | | | | -40.87 to 27.33 | | | No | | | ns | | | 0.9551 | | | |  | |  | | |  | |
| **anti-PD1 vs. anti-CD47** | | | | | | | 3.298 | | | | | -30.81 to 37.40 | | | No | | | ns | | | 0.9944 | | | |  | |  | | |  | |
| **anti-PD1 vs. anti-PD1 + anti-CD47** | | | | | | | -1.050 | | | | | -35.15 to 33.05 | | | No | | | ns | | | 0.9998 | | | |  | |  | | |  | |
| **anti-CD47 vs. anti-PD1 + anti-CD47** | | | | | | | -4.347 | | | | | -36.86 to 28.17 | | | No | | | ns | | | 0.9855 | | | |  | |  | | |  | |
|  | | | | | | |  | | | | |  | | |  | | |  | | |  | | | |  | |  | | |  | |
| **Row 5** | | | | | | |  | | | | |  | | |  | | |  | | |  | | | |  | |  | | |  | |
| **Control vs. anti-PD1** | | | | | | | 34.43 | | | | | -1.195 to 70.04 | | | No | | | ns | | | 0.0623 | | | |  | |  | | |  | |
| **Control vs. anti-CD47** | | | | | | | 9.342 | | | | | -24.76 to 43.44 | | | No | | | ns | | | 0.8921 | | | |  | |  | | |  | |
| **Control vs. anti-PD1 + anti-CD47** | | | | | | | 21.05 | | | | | -13.05 to 55.16 | | | No | | | ns | | | 0.3791 | | | |  | |  | | |  | |
| **anti-PD1 vs. anti-CD47** | | | | | | | -25.08 | | | | | -59.19 to 9.020 | | | No | | | ns | | | 0.2276 | | | |  | |  | | |  | |
| **anti-PD1 vs. anti-PD1 + anti-CD47** | | | | | | | -13.37 | | | | | -47.48 to 20.73 | | | No | | | ns | | | 0.7383 | | | |  | |  | | |  | |
| **anti-CD47 vs. anti-PD1 + anti-CD47** | | | | | | | 11.71 | | | | | -20.80 to 44.23 | | | No | | | ns | | | 0.7853 | | | |  | |  | | |  | |
|  | | | | | | |  | | | | |  | | |  | | |  | | |  | | | |  | |  | | |  | |
| **Row 6** | | | | | | |  | | | | |  | | |  | | |  | | |  | | | |  | |  | | |  | |
| **Control vs. anti-PD1** | | | | | | | 40.34 | | | | | 4.718 to 75.96 | | | Yes | | | * | | | 0.0196 | | | |  | |  | | |  | |
| **Control vs. anti-CD47** | | | | | | | 16.16 | | | | | -17.94 to 50.27 | | | No | | | ns | | | 0.6074 | | | |  | |  | | |  | |
| **Control vs. anti-PD1 + anti-CD47** | | | | | | | 53.74 | | | | | 19.64 to 87.85 | | | Yes | | | *** | | | 0.0004 | | | |  | |  | | |  | |
| **anti-PD1 vs. anti-CD47** | | | | | | | -24.17 | | | | | -58.28 to 9.930 | | | No | | | ns | | | 0.2578 | | | |  | |  | | |  | |
| **anti-PD1 vs. anti-PD1 + anti-CD47** | | | | | | | 13.40 | | | | | -20.70 to 47.51 | | | No | | | ns | | | 0.7369 | | | |  | |  | | |  | |
| **anti-CD47 vs. anti-PD1 + anti-CD47** | | | | | | | 37.58 | | | | | 5.062 to 70.09 | | | Yes | | | * | | | 0.0164 | | | |  | |  | | |  | |
|  | | | | | | |  | | | | |  | | |  | | |  | | |  | | | |  | |  | | |  | |
| **Row 7** | | | | | | |  | | | | |  | | |  | | |  | | |  | | | |  | |  | | |  | |
| **Control vs. anti-PD1** | | | | | | | 100.9 | | | | | 65.27 to 136.5 | | | Yes | | | **** | | | <0.0001 | | | |  | |  | | |  | |
| **Control vs. anti-CD47** | | | | | | | 90.62 | | | | | 56.52 to 124.7 | | | Yes | | | **** | | | <0.0001 | | | |  | |  | | |  | |
| **Control vs. anti-PD1 + anti-CD47** | | | | | | | 112.3 | | | | | 78.21 to 146.4 | | | Yes | | | **** | | | <0.0001 | | | |  | |  | | |  | |
| **anti-PD1 vs. anti-CD47** | | | | | | | -10.27 | | | | | -44.37 to 23.84 | | | No | | | ns | | | 0.8622 | | | |  | |  | | |  | |
| **anti-PD1 vs. anti-PD1 + anti-CD47** | | | | | | | 11.43 | | | | | -22.68 to 45.53 | | | No | | | ns | | | 0.8197 | | | |  | |  | | |  | |
| **anti-CD47 vs. anti-PD1 + anti-CD47** | | | | | | | 21.69 | | | | | -10.82 to 54.21 | | | No | | | ns | | | 0.3098 | | | |  | |  | | |  | |
|  | | | | | | |  | | | | |  | | |  | | |  | | |  | | | |  | |  | | |  | |
| **Row 8** | | | | | | |  | | | | |  | | |  | | |  | | |  | | | |  | |  | | |  | |
| **Control vs. anti-PD1** | | | | | | | 186.1 | | | | | 146.3 to 226.0 | | | Yes | | | **** | | | <0.0001 | | | |  | |  | | |  | |
| **Control vs. anti-CD47** | | | | | | | 192.0 | | | | | 154.2 to 229.7 | | | Yes | | | **** | | | <0.0001 | | | |  | |  | | |  | |
| **Control vs. anti-PD1 + anti-CD47** | | | | | | | 190.4 | | | | | 154.1 to 226.8 | | | Yes | | | **** | | | <0.0001 | | | |  | |  | | |  | |
| **anti-PD1 vs. anti-CD47** | | | | | | | 5.833 | | | | | -31.95 to 43.61 | | | No | | | ns | | | 0.9780 | | | |  | |  | | |  | |
| **anti-PD1 vs. anti-PD1 + anti-CD47** | | | | | | | 4.268 | | | | | -32.09 to 40.62 | | | No | | | ns | | | 0.9901 | | | |  | |  | | |  | |
| **anti-CD47 vs. anti-PD1 + anti-CD47** | | | | | | | -1.565 | | | | | -35.67 to 32.54 | | | No | | | ns | | | 0.9994 | | | |  | |  | | |  | |
|  | | | | | | |  | | | | |  | | |  | | |  | | |  | | | |  | |  | | |  | |
|  | | | | | | |  | | | | |  | | |  | | |  | | |  | | | |  | |  | | |  | |
| **Test details** | | | | | | | Predicted (LS) mean 1 | | | | | Predicted (LS) mean 2 | | | Predicted (LS) mean diff. | | | SE of diff. | | | N1 | | | | N2 | | q | | | DF | |
|  | | | | | | |  | | | | |  | | |  | | |  | | |  | | | |  | |  | | |  | |
| **Row 1** | | | | | | |  | | | | |  | | |  | | |  | | |  | | | |  | |  | | |  | |
| **Control vs. anti-PD1** | | | | | | | -2.842E-14 | | | | | -2.132E-14 | | | -7.105E-15 | | | 13.70 | | | 5 | | | | 5 | | 7.335E-16 | | | 141.0 | |
| **Control vs. anti-CD47** | | | | | | | -2.842E-14 | | | | | 0.000 | | | -2.842E-14 | | | 13.12 | | | 5 | | | | 6 | | 3.064E-15 | | | 141.0 | |
| **Control vs. anti-PD1 + anti-CD47** | | | | | | | -2.842E-14 | | | | | 0.000 | | | -2.842E-14 | | | 13.12 | | | 5 | | | | 6 | | 3.064E-15 | | | 141.0 | |
| **anti-PD1 vs. anti-CD47** | | | | | | | -2.132E-14 | | | | | 0.000 | | | -2.132E-14 | | | 13.12 | | | 5 | | | | 6 | | 2.298E-15 | | | 141.0 | |
| **anti-PD1 vs. anti-PD1 + anti-CD47** | | | | | | | -2.132E-14 | | | | | 0.000 | | | -2.132E-14 | | | 13.12 | | | 5 | | | | 6 | | 2.298E-15 | | | 141.0 | |
| **anti-CD47 vs. anti-PD1 + anti-CD47** | | | | | | | 0.000 | | | | | 0.000 | | | 0.000 | | | 12.51 | | | 6 | | | | 6 | | 0.000 | | | 141.0 | |
|  | | | | | | |  | | | | |  | | |  | | |  | | |  | | | |  | |  | | |  | |
| **Row 2** | | | | | | |  | | | | |  | | |  | | |  | | |  | | | |  | |  | | |  | |
| **Control vs. anti-PD1** | | | | | | | 6.287 | | | | | 8.875 | | | -2.587 | | | 13.70 | | | 5 | | | | 5 | | 0.2671 | | | 141.0 | |
| **Control vs. anti-CD47** | | | | | | | 6.287 | | | | | 8.063 | | | -1.775 | | | 13.12 | | | 5 | | | | 6 | | 0.1914 | | | 141.0 | |
| **Control vs. anti-PD1 + anti-CD47** | | | | | | | 6.287 | | | | | 10.59 | | | -4.306 | | | 13.12 | | | 5 | | | | 6 | | 0.4643 | | | 141.0 | |
| **anti-PD1 vs. anti-CD47** | | | | | | | 8.875 | | | | | 8.063 | | | 0.8125 | | | 13.12 | | | 5 | | | | 6 | | 0.08760 | | | 141.0 | |
| **anti-PD1 vs. anti-PD1 + anti-CD47** | | | | | | | 8.875 | | | | | 10.59 | | | -1.719 | | | 13.12 | | | 5 | | | | 6 | | 0.1853 | | | 141.0 | |
| **anti-CD47 vs. anti-PD1 + anti-CD47** | | | | | | | 8.063 | | | | | 10.59 | | | -2.531 | | | 12.51 | | | 6 | | | | 6 | | 0.2862 | | | 141.0 | |
|  | | | | | | |  | | | | |  | | |  | | |  | | |  | | | |  | |  | | |  | |
| **Row 3** | | | | | | |  | | | | |  | | |  | | |  | | |  | | | |  | |  | | |  | |
| **Control vs. anti-PD1** | | | | | | | 15.47 | | | | | 16.92 | | | -1.447 | | | 13.70 | | | 5 | | | | 5 | | 0.1493 | | | 141.0 | |
| **Control vs. anti-CD47** | | | | | | | 15.47 | | | | | 17.76 | | | -2.285 | | | 13.12 | | | 5 | | | | 6 | | 0.2464 | | | 141.0 | |
| **Control vs. anti-PD1 + anti-CD47** | | | | | | | 15.47 | | | | | 20.78 | | | -5.307 | | | 13.12 | | | 5 | | | | 6 | | 0.5722 | | | 141.0 | |
| **anti-PD1 vs. anti-CD47** | | | | | | | 16.92 | | | | | 17.76 | | | -0.8382 | | | 13.12 | | | 5 | | | | 6 | | 0.09037 | | | 141.0 | |
| **anti-PD1 vs. anti-PD1 + anti-CD47** | | | | | | | 16.92 | | | | | 20.78 | | | -3.861 | | | 13.12 | | | 5 | | | | 6 | | 0.4162 | | | 141.0 | |
| **anti-CD47 vs. anti-PD1 + anti-CD47** | | | | | | | 17.76 | | | | | 20.78 | | | -3.022 | | | 12.51 | | | 6 | | | | 6 | | 0.3418 | | | 141.0 | |
|  | | | | | | |  | | | | |  | | |  | | |  | | |  | | | |  | |  | | |  | |
| **Row 4** | | | | | | |  | | | | |  | | |  | | |  | | |  | | | |  | |  | | |  | |
| **Control vs. anti-PD1** | | | | | | | 30.45 | | | | | 36.17 | | | -5.719 | | | 13.70 | | | 5 | | | | 5 | | 0.5903 | | | 141.0 | |
| **Control vs. anti-CD47** | | | | | | | 30.45 | | | | | 32.88 | | | -2.421 | | | 13.12 | | | 5 | | | | 6 | | 0.2611 | | | 141.0 | |
| **Control vs. anti-PD1 + anti-CD47** | | | | | | | 30.45 | | | | | 37.22 | | | -6.769 | | | 13.12 | | | 5 | | | | 6 | | 0.7298 | | | 141.0 | |
| **anti-PD1 vs. anti-CD47** | | | | | | | 36.17 | | | | | 32.88 | | | 3.298 | | | 13.12 | | | 5 | | | | 6 | | 0.3555 | | | 141.0 | |
| **anti-PD1 vs. anti-PD1 + anti-CD47** | | | | | | | 36.17 | | | | | 37.22 | | | -1.050 | | | 13.12 | | | 5 | | | | 6 | | 0.1132 | | | 141.0 | |
| **anti-CD47 vs. anti-PD1 + anti-CD47** | | | | | | | 32.88 | | | | | 37.22 | | | -4.347 | | | 12.51 | | | 6 | | | | 6 | | 0.4916 | | | 141.0 | |
|  | | | | | | |  | | | | |  | | |  | | |  | | |  | | | |  | |  | | |  | |
| **Row 5** | | | | | | |  | | | | |  | | |  | | |  | | |  | | | |  | |  | | |  | |
| **Control vs. anti-PD1** | | | | | | | 88.44 | | | | | 54.02 | | | 34.43 | | | 13.70 | | | 5 | | | | 5 | | 3.554 | | | 141.0 | |
| **Control vs. anti-CD47** | | | | | | | 88.44 | | | | | 79.10 | | | 9.342 | | | 13.12 | | | 5 | | | | 6 | | 1.007 | | | 141.0 | |
| **Control vs. anti-PD1 + anti-CD47** | | | | | | | 88.44 | | | | | 67.39 | | | 21.05 | | | 13.12 | | | 5 | | | | 6 | | 2.270 | | | 141.0 | |
| **anti-PD1 vs. anti-CD47** | | | | | | | 54.02 | | | | | 79.10 | | | -25.08 | | | 13.12 | | | 5 | | | | 6 | | 2.704 | | | 141.0 | |
| **anti-PD1 vs. anti-PD1 + anti-CD47** | | | | | | | 54.02 | | | | | 67.39 | | | -13.37 | | | 13.12 | | | 5 | | | | 6 | | 1.442 | | | 141.0 | |
| **anti-CD47 vs. anti-PD1 + anti-CD47** | | | | | | | 79.10 | | | | | 67.39 | | | 11.71 | | | 12.51 | | | 6 | | | | 6 | | 1.324 | | | 141.0 | |
|  | | | | | | |  | | | | |  | | |  | | |  | | |  | | | |  | |  | | |  | |
| **Row 6** | | | | | | |  | | | | |  | | |  | | |  | | |  | | | |  | |  | | |  | |
| **Control vs. anti-PD1** | | | | | | | 90.59 | | | | | 50.25 | | | 40.34 | | | 13.70 | | | 5 | | | | 5 | | 4.164 | | | 141.0 | |
| **Control vs. anti-CD47** | | | | | | | 90.59 | | | | | 74.42 | | | 16.16 | | | 13.12 | | | 5 | | | | 6 | | 1.743 | | | 141.0 | |
| **Control vs. anti-PD1 + anti-CD47** | | | | | | | 90.59 | | | | | 36.85 | | | 53.74 | | | 13.12 | | | 5 | | | | 6 | | 5.794 | | | 141.0 | |
| **anti-PD1 vs. anti-CD47** | | | | | | | 50.25 | | | | | 74.42 | | | -24.17 | | | 13.12 | | | 5 | | | | 6 | | 2.606 | | | 141.0 | |
| **anti-PD1 vs. anti-PD1 + anti-CD47** | | | | | | | 50.25 | | | | | 36.85 | | | 13.40 | | | 13.12 | | | 5 | | | | 6 | | 1.445 | | | 141.0 | |
| **anti-CD47 vs. anti-PD1 + anti-CD47** | | | | | | | 74.42 | | | | | 36.85 | | | 37.58 | | | 12.51 | | | 6 | | | | 6 | | 4.249 | | | 141.0 | |
|  | | | | | | |  | | | | |  | | |  | | |  | | |  | | | |  | |  | | |  | |
| **Row 7** | | | | | | |  | | | | |  | | |  | | |  | | |  | | | |  | |  | | |  | |
| **Control vs. anti-PD1** | | | | | | | 151.1 | | | | | 50.19 | | | 100.9 | | | 13.70 | | | 5 | | | | 5 | | 10.41 | | | 141.0 | |
| **Control vs. anti-CD47** | | | | | | | 151.1 | | | | | 60.45 | | | 90.62 | | | 13.12 | | | 5 | | | | 6 | | 9.770 | | | 141.0 | |
| **Control vs. anti-PD1 + anti-CD47** | | | | | | | 151.1 | | | | | 38.76 | | | 112.3 | | | 13.12 | | | 5 | | | | 6 | | 12.11 | | | 141.0 | |
| **anti-PD1 vs. anti-CD47** | | | | | | | 50.19 | | | | | 60.45 | | | -10.27 | | | 13.12 | | | 5 | | | | 6 | | 1.107 | | | 141.0 | |
| **anti-PD1 vs. anti-PD1 + anti-CD47** | | | | | | | 50.19 | | | | | 38.76 | | | 11.43 | | | 13.12 | | | 5 | | | | 6 | | 1.232 | | | 141.0 | |
| **anti-CD47 vs. anti-PD1 + anti-CD47** | | | | | | | 60.45 | | | | | 38.76 | | | 21.69 | | | 12.51 | | | 6 | | | | 6 | | 2.453 | | | 141.0 | |
|  | | | | | | |  | | | | |  | | |  | | |  | | |  | | | |  | |  | | |  | |
| **Row 8** | | | | | | |  | | | | |  | | |  | | |  | | |  | | | |  | |  | | |  | |
| **Control vs. anti-PD1** | | | | | | | 213.1 | | | | | 27.00 | | | 186.1 | | | 15.32 | | | 4 | | | | 4 | | 17.19 | | | 141.0 | |
| **Control vs. anti-CD47** | | | | | | | 213.1 | | | | | 21.17 | | | 192.0 | | | 14.53 | | | 4 | | | | 5 | | 18.68 | | | 141.0 | |
| **Control vs. anti-PD1 + anti-CD47** | | | | | | | 213.1 | | | | | 22.73 | | | 190.4 | | | 13.98 | | | 4 | | | | 6 | | 19.26 | | | 141.0 | |
| **anti-PD1 vs. anti-CD47** | | | | | | | 27.00 | | | | | 21.17 | | | 5.833 | | | 14.53 | | | 4 | | | | 5 | | 0.5677 | | | 141.0 | |
| **anti-PD1 vs. anti-PD1 + anti-CD47** | | | | | | | 27.00 | | | | | 22.73 | | | 4.268 | | | 13.98 | | | 4 | | | | 6 | | 0.4317 | | | 141.0 | |
| **anti-CD47 vs. anti-PD1 + anti-CD47** | | | | | | | 21.17 | | | | | 22.73 | | | -1.565 | | | 13.12 | | | 5 | | | | 6 | | 0.1687 | | | 141.0 | |

**Figure 2C**

|  | **Control** | **Control** | **Control** | **Control** | **Control** | **Control** | **Control** | **Control** | **Control** | **Control** | **Anti PD-1 + tdRT** | **Anti PD-1 + tdRT** | **Anti PD-1 + tdRT** | **Anti PD-1 + tdRT** | **Anti PD-1 + tdRT** | **Anti PD-1 + tdRT** | **Anti PD-1 + tdRT** | **Anti PD-1 + tdRT** | **Anti PD-1 + tdRT** | **Anti PD-1 + tdRT** | **Anti PD-1 + Anti CD47** | **Anti PD-1 + Anti CD47** | **Anti PD-1 + Anti CD47** | **Anti PD-1 + Anti CD47** | **Anti PD-1 + Anti CD47** | **Anti PD-1 + Anti CD47** | **Anti PD-1 + Anti CD47** | **Anti PD-1 + Anti CD47** | **Anti PD-1 + Anti CD47** | **Anti PD-1 + Anti CD47** | **Anti PD-1 + Anti CD47 + tdRT** | **Anti PD-1 + Anti CD47 + tdRT** | **Anti PD-1 + Anti CD47 + tdRT** | **Anti PD-1 + Anti CD47 + tdRT** | **Anti PD-1 + Anti CD47 + tdRT** | **Anti PD-1 + Anti CD47 + tdRT** | **Anti PD-1 + Anti CD47 + tdRT** | **Anti PD-1 + Anti CD47 + tdRT** | **Anti PD-1 + Anti CD47 + tdRT** | **Anti PD-1 + Anti CD47 + tdRT** |
| --- | --- | --- | --- | --- | --- | --- | --- | --- | --- | --- | --- | --- | --- | --- | --- | --- | --- | --- | --- | --- | --- | --- | --- | --- | --- | --- | --- | --- | --- | --- | --- | --- | --- | --- | --- | --- | --- | --- | --- | --- |
| **0** | 0.00 | 0 | 0.000 | 0.00 | 0.0000 | 0.0000 | 0.00 | 0.00 | 0.0000 | 0.000 | 0.00 | 0.0000 | 0.000 | 0.0000 | 0.000 | 0.0000 | 0.000 | 0.000 | 0.000 | 0.000 | 0.000 | 0.000 | 0.000 | 0.000 | 0.000 | 0.000 | 0.000 | 0.000 | 0.000 | 0.000 | 0.000 | 0.000 | 0.000 | 0.000 | 0.000 | 0.000 | 0.000 | 0.000 | 0.000 | 0.000 |
| **4** | 0.00 | 24 | 30.375 | 28.00 | 21.4375 | 15.7500 | 28.00 | 32.00 | 24.0000 | 32.000 | 15.75 | 21.4375 | 18.375 | 30.3750 | 24.500 | 35.4375 | 13.500 | 11.250 | 13.500 | 18.375 | 32.000 | 21.438 | 13.500 | 15.750 | 15.750 | 24.500 | 15.750 | 32.000 | 28.000 | 7.813 | 21.438 | 21.438 | 28.000 | 21.438 | 36.000 | 15.750 | 13.500 | 21.438 | 15.750 | 15.750 |
| **6** | 43.75 | 90 | 122.500 | 90.00 | 81.0000 | 56.2500 | 62.50 | 90.00 | 95.0625 | 56.250 | 90.00 | 90.0000 | 44.000 | 72.0000 | 90.000 | 90.0000 | 56.250 | 30.375 | 40.000 | 83.188 | 50.000 | 60.500 | 28.000 | 43.750 | 40.000 | 60.500 | 40.500 | 56.250 | 50.000 | 18.375 | 68.750 | 62.500 | 62.500 | 56.250 | 62.500 | 122.500 | 32.000 | 50.000 | 36.000 | 68.750 |
| **8** | 60.50 | 147 |  | 168.75 | 122.5000 | 154.6875 | 126.75 | 183.75 | 134.7500 | 147.875 | 117.00 | 108.0000 | 87.500 | 126.0000 | 126.750 | 208.0000 | 81.250 | 90.000 | 56.250 | 99.000 | 108.000 | 117.000 | 134.750 | 75.625 | 81.250 | 75.000 | 81.250 | 90.750 | 134.750 | 62.500 | 108.000 | 116.188 | 83.188 | 126.750 | 108.000 | 198.688 | 44.000 | 108.000 | 44.000 | 99.000 |
| **10** | 0.00* | 0* | 0.000* | 0.00* | 147.0000 | 192.0000 | 126.75 | 0.00* | 0.0000* | 0.000* | 0.00* | 0.0000* | 0.000* | 99.0000 | 62.500 | 122.5000 | 40.000 | 0.000* | 68.750 | 68.750 | 0.000* | 83.188 | 90.000 | 75.625 | 0.000* | 68.750 | 68.750 | 83.188 | 90.000 | 56.250 | 50.625 | 55.688 | 0.000* | 75.625 | 83.188 | 234.813 | 0.000* | 116.188 | 32.000 | 90.750 |
| **12** | 0.00* | 0* | 0.000* | 0.00* | 0.0000* | 0.0000* | 0.00* | 0.00* | 0.0000* | 0.000* | 0.00* | 0.0000* | 0.000* | 0.0000* | 0.000* | 81.0000 | 13.500 | 0.000* | 24.500 | 0.000* | 0.000* | 0.000* | 35.438 | 0.000* | 0.000* | 0.000* | 0.000* | 32.000 | 0.000* | 95.063 | 0.000* | 0.000* | 0.000* | 40.500 | 0.000* | 0.000* | 0.000* | 0.000* | 13.500 | 0.000* |
| **14** | 0.00* | 0* | 0.000* | 0.00* | 0.0000* | 0.0000* | 0.00* | 0.00* | 0.0000* | 0.000* | 0.00* | 0.0000* | 0.000* | 0.0000* | 0.000* | 50.0000 | 13.500 | 0.000* | 15.750 | 0.000* | 0.000* | 0.000* | 28.000 | 0.000* | 0.000* | 0.000* | 0.000* | 36.000 | 0.000* | 72.000 | 0.000* | 0.000* | 0.000* | 13.500 | 0.000* | 0.000* | 0.000* | 0.000* | 9.375 | 0.000* |
| **16** | 0.00* | 0* | 0.000* | 0.00* | 0.0000* | 0.0000* | 0.00* | 0.00* | 0.0000* | 0.000* | 0.00* | 0.0000* | 0.000* | 0.0000* | 0.000* | 50.0000 | 11.250 | 0.000* | 9.375 | 0.000* | 0.000* | 0.000* | 15.750 | 0.000* | 0.000* | 0.000* | 0.000* | 28.000 | 0.000* | 50.000 | 0.000* | 0.000* | 0.000* | 11.250 | 0.000* | 0.000* | 0.000* | 0.000* | 7.813 | 0.000* |
| **18** | 0.00* | 0* | 0.000* | 0.00* | 0.0000* | 0.0000* | 0.00* | 0.00* | 0.0000* | 0.000* | 0.00* | 0.0000* | 0.000* | 0.0000* | 0.000* | 24.0000 | 4.000 | 0.000* | 7.813 | 0.000* | 0.000* | 0.000* | 9.375 | 0.000* | 0.000* | 0.000* | 0.000* | 13.500 | 0.000* | 56.250 | 0.000* | 0.000* | 0.000* | 2.250 | 0.000* | 0.000* | 0.000* | 0.000* | 4.000 | 0.000* |
| **20** |  |  |  |  |  |  |  |  |  |  |  |  |  |  |  |  |  |  |  |  |  |  |  |  |  |  |  |  |  |  |  |  |  |  |  |  |  |  |  |  |

| **Compare column means (main column effect)** |  |  |  |  |  |  |  |  |
| --- | --- | --- | --- | --- | --- | --- | --- | --- |
|  |  |  |  |  |  |  |  |  |
| **Number of families** | 1 |  |  |  |  |  |  |  |
| **Number of comparisons per family** | 6 |  |  |  |  |  |  |  |
| **Alpha** | 0.05 |  |  |  |  |  |  |  |
|  |  |  |  |  |  |  |  |  |
| **Tukey's multiple comparisons test** | Mean Diff. | 95.00% CI of diff. | Below threshold? | Summary | Adjusted P Value |  |  |  |
|  |  |  |  |  |  |  |  |  |
| **Control vs. Anti PD-1 + tdRT** | 17.05 | 3.164 to 30.93 | Yes | ** | 0.0091 |  |  |  |
| **Control vs. Anti PD-1 + Anti CD47** | 20.95 | 7.142 to 34.75 | Yes | *** | 0.0007 |  |  |  |
| **Control vs. Anti PD-1 + Anti CD47 + tdRT** | 19.01 | 5.129 to 32.88 | Yes | ** | 0.0027 |  |  |  |
| **Anti PD-1 + tdRT vs. Anti PD-1 + Anti CD47** | 3.901 | -8.448 to 16.25 | No | ns | 0.8458 |  |  |  |
| **Anti PD-1 + tdRT vs. Anti PD-1 + Anti CD47 + tdRT** | 1.960 | -10.63 to 14.55 | No | ns | 0.9778 |  |  |  |
| **Anti PD-1 + Anti CD47 vs. Anti PD-1 + Anti CD47 + tdRT** | -1.942 | -14.42 to 10.53 | No | ns | 0.9778 |  |  |  |
|  |  |  |  |  |  |  |  |  |
|  |  |  |  |  |  |  |  |  |
| **Test details** | Mean 1 | Mean 2 | Mean Diff. | SE of diff. | N1 | N2 | q | DF |
|  |  |  |  |  |  |  |  |  |
| **Control vs. Anti PD-1 + tdRT** | 60.84 | 43.80 | 17.05 | 5.359 | 42 | 58 | 4.498 | 204.0 |
| **Control vs. Anti PD-1 + Anti CD47** | 60.84 | 39.90 | 20.95 | 5.330 | 42 | 60 | 5.558 | 204.0 |
| **Control vs. Anti PD-1 + Anti CD47 + tdRT** | 60.84 | 41.84 | 19.01 | 5.357 | 42 | 56 | 5.017 | 204.0 |
| **Anti PD-1 + tdRT vs. Anti PD-1 + Anti CD47** | 43.80 | 39.90 | 3.901 | 4.767 | 58 | 60 | 1.157 | 204.0 |
| **Anti PD-1 + tdRT vs. Anti PD-1 + Anti CD47 + tdRT** | 43.80 | 41.84 | 1.960 | 4.861 | 58 | 56 | 0.5701 | 204.0 |
| **Anti PD-1 + Anti CD47 vs. Anti PD-1 + Anti CD47 + tdRT** | 39.90 | 41.84 | -1.942 | 4.816 | 60 | 56 | 0.5701 | 204.0 |

**Figure 3B**

| **Days** | **Control** | **Control** | **Control** | **Control** | **Control** | **anti-CD47** | **anti-CD47** | **anti-CD47** | **anti-CD47** | **anti-CD47** |
| --- | --- | --- | --- | --- | --- | --- | --- | --- | --- | --- |
| **0** | 0.00 | 0.0000 | 0.0000 | 0.0000 | 0.0000 | 0.0000 | 0.00 | 0.0000 | 0.0000 | 0.0000 |
| **5** | 24.50 | 27.5625 | 21.4375 | 32.0000 | 24.5000 | 21.4375 | 28.00 | 27.5625 | 24.5000 | 24.5000 |
| **8** | 40.00 | 44.0000 | 36.0000 | 40.0000 | 36.0000 | 45.5625 | 62.50 | 40.0000 | 68.7500 | 55.6875 |
| **10** | 60.75 | 48.0000 | 40.0000 | 65.8125 | 44.0000 | 45.5625 | 68.75 | 27.5625 | 50.6250 | 45.5625 |
| **12** | 81.25 | 60.7500 | 50.6250 | 121.0000 | 60.7500 | 45.5625 | 56.25 | 18.0000 | 60.7500 | 60.7500 |
| **14** | 87.50 | 55.6875 | 60.7500 | 162.0000 | 98.3125 | 40.0000 | 62.50 | 13.5000 | 55.6875 | 55.6875 |

| **Table Analyzed** | **4MOSC1 Flow/TCR Seq** |  |  |  |  |
| --- | --- | --- | --- | --- | --- |
|  |  |  |  |  |  |
| **Two-way RM ANOVA** | Matching: Stacked |  |  |  |  |
| **Assume sphericity?** | No |  |  |  |  |
| **Alpha** | 0.05 |  |  |  |  |
|  |  |  |  |  |  |
| **Source of Variation** | % of total variation | P value | P value summary | Significant? | Geisser-Greenhouse's epsilon |
| **Time x Column Factor** | 11.36 | 0.0003 | *** | Yes |  |
| **Time** | 58.12 | <0.0001 | **** | Yes | 0.2715 |
| **Column Factor** | 3.026 | 0.2020 | ns | No |  |
| **Subject** | 12.53 | 0.0010 | ** | Yes |  |
|  |  |  |  |  |  |
| **ANOVA table** | SS | DF | MS | F (DFn, DFd) | P value |
| **Time x Column Factor** | 6326 | 5 | 1265 | F (5, 40) = 6.074 | P=0.0003 |
| **Time** | 32358 | 5 | 6472 | F (1.357, 10.86) = 31.07 | P<0.0001 |
| **Column Factor** | 1685 | 1 | 1685 | F (1, 8) = 1.932 | P=0.2020 |
| **Subject** | 6977 | 8 | 872.2 | F (8, 40) = 4.187 | P=0.0010 |
| **Residual** | 8332 | 40 | 208.3 |  |  |
|  |  |  |  |  |  |
| **Difference between column means** |  |  |  |  |  |
| **Mean of Control** | 47.44 |  |  |  |  |
| **Mean of anti-CD47** | 36.84 |  |  |  |  |
| **Difference between means** | 10.60 |  |  |  |  |
| **SE of difference** | 7.625 |  |  |  |  |
| **95% CI of difference** | -6.986 to 28.18 |  |  |  |  |
|  |  |  |  |  |  |
| **Data summary** |  |  |  |  |  |
| **Number of columns (Column Factor)** | 2 |  |  |  |  |
| **Number of rows (Time)** | 6 |  |  |  |  |
| **Number of subjects (Subject)** | 10 |  |  |  |  |
| **Number of missing values** | 0 |  |  |  |  |

**Figure 3C**

Tumor: MHC II MFI (Median) in CD11c+ MHC II+ DC

| **Control** | **αCD47** |
| --- | --- |
| 363318.00 | 406000.00 |
| 243024.00 | 871000.00 |
| 329704.00 | 394849.00 |
| 363318.00 | 458000.00 |

SLN: MHC II MFI (Median) in CD11c+ MHC II+ DC

| **Control** | **αCD47** |
| --- | --- |
| 43628.00 | 81985.00 |
| 126738.00 | 34069.00 |
| 200857.00 | 83413.00 |
| 20538.00 | 56287.00 |

CLN: MHC II MFI (Median) in CD11c+ MHC II+ DC

| **Control** | **αCD47** |
| --- | --- |
| 190028.00 | 192013.00 |
| 177926.00 | 242183.00 |
| 164304.00 | 181034.00 |
| 109981.00 | 255106.00 |

| **Table Analyzed** | **Tumor: MHC II MFI (Median) in CD11c+ MHC II+ DC** |
| --- | --- |
|  |  |
| **Column B** | αCD47 |
| **vs.** | vs. |
| **Column A** | Control |
|  |  |
| **Unpaired t test** |  |
| **P value** | 0.1268 |
| **P value summary** | ns |
| **Significantly different (P < 0.05)?** | No |
| **One- or two-tailed P value?** | Two-tailed |
| **t, df** | t=1.772, df=6 |
|  |  |
| **How big is the difference?** |  |
| **Mean of column A** | 324841 |
| **Mean of column B** | 532462 |
| **Difference between means (B - A) ± SEM** | 207621 ± 117175 |
| **95% confidence interval** | -79097 to 494339 |
| **R squared (eta squared)** | 0.3435 |
|  |  |
| **F test to compare variances** |  |
| **F, DFn, Dfd** | 16.02, 3, 3 |
| **P value** | 0.0475 |
| **P value summary** | * |
| **Significantly different (P < 0.05)?** | Yes |
|  |  |
| **Data analyzed** |  |
| **Sample size, column A** | 4 |
| **Sample size, column B** | 4 |

| **Table Analyzed** | **sLN: MHC II MFI (Median) in CD11c+ MHC II+ DC** |
| --- | --- |
|  |  |
| **Column B** | αCD47 |
| **vs.** | vs. |
| **Column A** | Control |
|  |  |
| **Unpaired t test** |  |
| **P value** | 0.4576 |
| **P value summary** | ns |
| **Significantly different (P < 0.05)?** | No |
| **One- or two-tailed P value?** | Two-tailed |
| **t, df** | t=0.7938, df=6 |
|  |  |
| **How big is the difference?** |  |
| **Mean of column A** | 97940 |
| **Mean of column B** | 63939 |
| **Difference between means (B - A) ± SEM** | -34002 ± 42835 |
| **95% confidence interval** | -138815 to 70811 |
| **R squared (eta squared)** | 0.09504 |
|  |  |
| **F test to compare variances** |  |
| **F, DFn, Dfd** | 12.30, 3, 3 |
| **P value** | 0.0684 |
| **P value summary** | ns |
| **Significantly different (P < 0.05)?** | No |
|  |  |
| **Data analyzed** |  |
| **Sample size, column A** | 4 |
| **Sample size, column B** | 4 |

| **Table Analyzed** | **cLN: MHC II MFI (Median) in CD11c+ MHC II+ DC** |
| --- | --- |
|  |  |
| **Column B** | αCD47 |
| **vs.** | vs. |
| **Column A** | Control |
|  |  |
| **Unpaired t test** |  |
| **P value** | 0.0659 |
| **P value summary** | ns |
| **Significantly different (P < 0.05)?** | No |
| **One- or two-tailed P value?** | Two-tailed |
| **t, df** | t=2.245, df=6 |
|  |  |
| **How big is the difference?** |  |
| **Mean of column A** | 160560 |
| **Mean of column B** | 217584 |
| **Difference between means (B - A) ± SEM** | 57024 ± 25405 |
| **95% confidence interval** | -5140 to 119188 |
| **R squared (eta squared)** | 0.4564 |
|  |  |
| **F test to compare variances** |  |
| **F, DFn, Dfd** | 1.070, 3, 3 |
| **P value** | 0.9572 |
| **P value summary** | ns |
| **Significantly different (P < 0.05)?** | No |
|  |  |
| **Data analyzed** |  |
| **Sample size, column A** | 4 |
| **Sample size, column B** | 4 |

**Figure 3D**

Tumor: CD86 MFI (Median) in CD11c+ MHC II+ DC

| **Control** | **αCD47** |
| --- | --- |
| 33269.00 | 44082.00 |
| 32994.00 | 59725.00 |
| 33828.00 | 44082.00 |
| 39910.00 | 52998.00 |

SLN: CD86 MFI (Median) in CD11c+ MHC II+ DC

| **Control** | **αCD47** |
| --- | --- |
| 26659.00 | 66194.00 |
| 14488.00 | 44334.00 |
| 30627.00 | 46679.00 |
| 43706.00 | 72337.00 |

CLN: CD86 MFI (Median) in CD11c+ MHC II+ DC

| **Control** | **αCD47** |
| --- | --- |
| 15599.00 | 54086.00 |
| 16895.00 | 60075.00 |
| 21612.00 | 45751.00 |
| 20675.00 | 41056.00 |

| **Table Analyzed** | **Tumor: CD86 MFI (Median) in CD11c+ MHC II+ DC** |
| --- | --- |
|  |  |
| **Column B** | αCD47 |
| **vs.** | vs. |
| **Column A** | Control |
|  |  |
| **Unpaired t test** |  |
| **P value** | 0.0104 |
| **P value summary** | * |
| **Significantly different (P < 0.05)?** | Yes |
| **One- or two-tailed P value?** | Two-tailed |
| **t, df** | t=3.675, df=6 |
|  |  |
| **How big is the difference?** |  |
| **Mean of column A** | 35000 |
| **Mean of column B** | 50222 |
| **Difference between means (B - A) ± SEM** | 15222 ± 4142 |
| **95% confidence interval** | 5085 to 25358 |
| **R squared (eta squared)** | 0.6923 |
|  |  |
| **F test to compare variances** |  |
| **F, DFn, Dfd** | 5.335, 3, 3 |
| **P value** | 0.2025 |
| **P value summary** | ns |
| **Significantly different (P < 0.05)?** | No |
|  |  |
| **Data analyzed** |  |
| **Sample size, column A** | 4 |
| **Sample size, column B** | 4 |

| **Table Analyzed** | **sLN: CD86 MFI (Median) in CD11c+ MHC II+ DC** |
| --- | --- |
|  |  |
| **Column B** | αCD47 |
| **vs.** | vs. |
| **Column A** | Control |
|  |  |
| **Unpaired t test** |  |
| **P value** | 0.0213 |
| **P value summary** | * |
| **Significantly different (P < 0.05)?** | Yes |
| **One- or two-tailed P value?** | Two-tailed |
| **t, df** | t=3.091, df=6 |
|  |  |
| **How big is the difference?** |  |
| **Mean of column A** | 28870 |
| **Mean of column B** | 57386 |
| **Difference between means (B - A) ± SEM** | 28516 ± 9224 |
| **95% confidence interval** | 5945 to 51087 |
| **R squared (eta squared)** | 0.6143 |
|  |  |
| **F test to compare variances** |  |
| **F, DFn, Dfd** | 1.348, 3, 3 |
| **P value** | 0.8122 |
| **P value summary** | ns |
| **Significantly different (P < 0.05)?** | No |
|  |  |
| **Data analyzed** |  |
| **Sample size, column A** | 4 |
| **Sample size, column B** | 4 |

| **Table Analyzed** | **cLN: CD86 MFI (Median) in CD11c+ MHC II+ DC** |
| --- | --- |
|  |  |
| **Column B** | αCD47 |
| **vs.** | vs. |
| **Column A** | Control |
|  |  |
| **Unpaired t test** |  |
| **P value** | 0.0004 |
| **P value summary** | *** |
| **Significantly different (P < 0.05)?** | Yes |
| **One- or two-tailed P value?** | Two-tailed |
| **t, df** | t=7.035, df=6 |
|  |  |
| **How big is the difference?** |  |
| **Mean of column A** | 18695 |
| **Mean of column B** | 50242 |
| **Difference between means (B - A) ± SEM** | 31547 ± 4484 |
| **95% confidence interval** | 20575 to 42519 |
| **R squared (eta squared)** | 0.8919 |
|  |  |
| **F test to compare variances** |  |
| **F, DFn, Dfd** | 8.554, 3, 3 |
| **P value** | 0.1113 |
| **P value summary** | ns |
| **Significantly different (P < 0.05)?** | No |
|  |  |
| **Data analyzed** |  |
| **Sample size, column A** | 4 |
| **Sample size, column B** | 4 |

**Figure 4**

Tumor: CD69 MFI (Median) in CD8+

| **Control** | **ALX301** | **anti-PD1** | **anti-PD1 + ALX301** | **tdRT + anti-PD1** | **tdRT + anti-PD1 + ALX301** |
| --- | --- | --- | --- | --- | --- |
| 1974.00 | 2868.00 | 4681.00 | 3988.00 | 6218.00 | 3667.00 |
| 1854.00 | 2847.00 | 4693.00 | 3193.00 | 5575.00 | 3471.00 |
| 1874.00 | 2487.00 | 4434.00 | 4000.00 | 5294.00 | 4551.00 |
| 2003.00 | 2548.00 | 3385.00 | 4670.00 | 7618.00 | 5045.00 |
| 1765.00 | 2630.00 | 6428.00 | 4825.00 | 4777.00 | 4813.00 |
| 4481.00 | 3077.00 | 2920.00 | 3733.00 | 4318.00 | 5243.00 |
| 2579.00 | 3151.00 | 3471.00 | 3910.00 | 4056.00 | 5395.00 |

| **Number of families** | **1** |  |  |  |  |  |  |  |
| --- | --- | --- | --- | --- | --- | --- | --- | --- |
| **Number of comparisons per family** | 15 |  |  |  |  |  |  |  |
| **Alpha** | 0.05 |  |  |  |  |  |  |  |
|  |  |  |  |  |  |  |  |  |
| **Tukey's multiple comparisons test** | Mean Diff. | 95.00% CI of diff. | Below threshold? | Summary | Adjusted P Value |  |  |  |
| **Control vs. αCD47** | -3.086 | -12.94 to 6.765 | No | ns | 0.9326 | A-B |  |  |
| **Control vs. αPD-1** | -17.33 | -27.18 to -7.478 | Yes | **** | <0.0001 | A-C |  |  |
| **Control vs. αPD-1 + αCD47** | -16.09 | -25.94 to -6.235 | Yes | *** | 0.0003 | A-D |  |  |
| **Control vs. tdRT + αPD-1** | -24.60 | -34.45 to -14.75 | Yes | **** | <0.0001 | A-E |  |  |
| **Control vs. tdRT + αPD-1 + αCD47** | -19.36 | -29.21 to -9.506 | Yes | **** | <0.0001 | A-F |  |  |
| **αCD47 vs. αPD-1** | -14.24 | -24.09 to -4.392 | Yes | ** | 0.0014 | B-C |  |  |
| **αCD47 vs. αPD-1 + αCD47** | -13.00 | -22.85 to -3.149 | Yes | ** | 0.0041 | B-D |  |  |
| **αCD47 vs. tdRT + αPD-1** | -21.51 | -31.36 to -11.66 | Yes | **** | <0.0001 | B-E |  |  |
| **αCD47 vs. tdRT + αPD-1 + αCD47** | -16.27 | -26.12 to -6.421 | Yes | *** | 0.0002 | B-F |  |  |
| **αPD-1 vs. αPD-1 + αCD47** | 1.243 | -8.608 to 11.09 | No | ns | 0.9989 | C-D |  |  |
| **αPD-1 vs. tdRT + αPD-1** | -7.271 | -17.12 to 2.579 | No | ns | 0.2535 | C-E |  |  |
| **αPD-1 vs. tdRT + αPD-1 + αCD47** | -2.029 | -11.88 to 7.822 | No | ns | 0.9889 | C-F |  |  |
| **αPD-1 + αCD47 vs. tdRT + αPD-1** | -8.514 | -18.36 to 1.336 | No | ns | 0.1233 | D-E |  |  |
| **αPD-1 + αCD47 vs. tdRT + αPD-1 + αCD47** | -3.271 | -13.12 to 6.579 | No | ns | 0.9152 | D-F |  |  |
| **tdRT + αPD-1 vs. tdRT + αPD-1 + αCD47** | 5.243 | -4.608 to 15.09 | No | ns | 0.6031 | E-F |  |  |
|  |  |  |  |  |  |  |  |  |
| **Test details** | Mean 1 | Mean 2 | Mean Diff. | SE of diff. | n1 | n2 | q | DF |
| **Control vs. αCD47** | 33.09 | 36.17 | -3.086 | 3.274 | 7 | 7 | 1.333 | 36 |
| **Control vs. αPD-1** | 33.09 | 50.41 | -17.33 | 3.274 | 7 | 7 | 7.485 | 36 |
| **Control vs. αPD-1 + αCD47** | 33.09 | 49.17 | -16.09 | 3.274 | 7 | 7 | 6.948 | 36 |
| **Control vs. tdRT + αPD-1** | 33.09 | 57.69 | -24.60 | 3.274 | 7 | 7 | 10.63 | 36 |
| **Control vs. tdRT + αPD-1 + αCD47** | 33.09 | 52.44 | -19.36 | 3.274 | 7 | 7 | 8.361 | 36 |
| **αCD47 vs. αPD-1** | 36.17 | 50.41 | -14.24 | 3.274 | 7 | 7 | 6.152 | 36 |
| **αCD47 vs. αPD-1 + αCD47** | 36.17 | 49.17 | -13.00 | 3.274 | 7 | 7 | 5.615 | 36 |
| **αCD47 vs. tdRT + αPD-1** | 36.17 | 57.69 | -21.51 | 3.274 | 7 | 7 | 9.293 | 36 |
| **αCD47 vs. tdRT + αPD-1 + αCD47** | 36.17 | 52.44 | -16.27 | 3.274 | 7 | 7 | 7.028 | 36 |
| **αPD-1 vs. αPD-1 + αCD47** | 50.41 | 49.17 | 1.243 | 3.274 | 7 | 7 | 0.5368 | 36 |
| **αPD-1 vs. tdRT + αPD-1** | 50.41 | 57.69 | -7.271 | 3.274 | 7 | 7 | 3.141 | 36 |
| **αPD-1 vs. tdRT + αPD-1 + αCD47** | 50.41 | 52.44 | -2.029 | 3.274 | 7 | 7 | 0.8762 | 36 |
| **αPD-1 + αCD47 vs. tdRT + αPD-1** | 49.17 | 57.69 | -8.514 | 3.274 | 7 | 7 | 3.678 | 36 |
| **αPD-1 + αCD47 vs. tdRT + αPD-1 + αCD47** | 49.17 | 52.44 | -3.271 | 3.274 | 7 | 7 | 1.413 | 36 |
| **tdRT + αPD-1 vs. tdRT + αPD-1 + αCD47** | 57.69 | 52.44 | 5.243 | 3.274 | 7 | 7 | 2.265 | 36 |
|  |  |  |  |  |  |  |  |  |
| **Compact letter display** |  |  |  |  |  |  |  |  |
| **tdRT + αPD-1** | A |  |  |  |  |  |  |  |
| **tdRT + αPD-1 + αCD47** | A |  |  |  |  |  |  |  |
| **αPD-1** | A |  |  |  |  |  |  |  |
| **αPD-1 + αCD47** | A |  |  |  |  |  |  |  |
| **αCD47** | B |  |  |  |  |  |  |  |
| **Control** | B |  |  |  |  |  |  |  |

**Supplemental Figure 1C**

|  | **Control** | **Control** | **Control** | **Control** | **Control** | **Control** | **Control** | **Control** | **Control** | **Control** | **Anti PD-1 + tdRT** | **Anti PD-1 + tdRT** | **Anti PD-1 + tdRT** | **Anti PD-1 + tdRT** | **Anti PD-1 + tdRT** | **Anti PD-1 + tdRT** | **Anti PD-1 + tdRT** | **Anti PD-1 + tdRT** | **Anti PD-1 + tdRT** | **Anti PD-1 + tdRT** | **Anti PD-1 + Anti CD47** | **Anti PD-1 + Anti CD47** | **Anti PD-1 + Anti CD47** | **Anti PD-1 + Anti CD47** | **Anti PD-1 + Anti CD47** | **Anti PD-1 + Anti CD47** | **Anti PD-1 + Anti CD47** | **Anti PD-1 + Anti CD47** | **Anti PD-1 + Anti CD47** | **Anti PD-1 + Anti CD47** | **Anti PD-1 + Anti CD47 + tdRT** | **Anti PD-1 + Anti CD47 + tdRT** | **Anti PD-1 + Anti CD47 + tdRT** | **Anti PD-1 + Anti CD47 + tdRT** | **Anti PD-1 + Anti CD47 + tdRT** | **Anti PD-1 + Anti CD47 + tdRT** | **Anti PD-1 + Anti CD47 + tdRT** | **Anti PD-1 + Anti CD47 + tdRT** | **Anti PD-1 + Anti CD47 + tdRT** | **Anti PD-1 + Anti CD47 + tdRT** |
| --- | --- | --- | --- | --- | --- | --- | --- | --- | --- | --- | --- | --- | --- | --- | --- | --- | --- | --- | --- | --- | --- | --- | --- | --- | --- | --- | --- | --- | --- | --- | --- | --- | --- | --- | --- | --- | --- | --- | --- | --- |
| **0** | 0.00 | 0 | 0.000 | 0.00 | 0.0000 | 0.0000 | 0.00 | 0.00 | 0.0000 | 0.000 | 0.00 | 0.0000 | 0.000 | 0.0000 | 0.000 | 0.0000 | 0.000 | 0.000 | 0.000 | 0.000 | 0.000 | 0.000 | 0.000 | 0.000 | 0.000 | 0.000 | 0.000 | 0.000 | 0.000 | 0.000 | 0.000 | 0.000 | 0.000 | 0.000 | 0.000 | 0.000 | 0.000 | 0.000 | 0.000 | 0.000 |
| **4** | 0.00 | 24 | 30.375 | 28.00 | 21.4375 | 15.7500 | 28.00 | 32.00 | 24.0000 | 32.000 | 15.75 | 21.4375 | 18.375 | 30.3750 | 24.500 | 35.4375 | 13.500 | 11.250 | 13.500 | 18.375 | 32.000 | 21.438 | 13.500 | 15.750 | 15.750 | 24.500 | 15.750 | 32.000 | 28.000 | 7.813 | 21.438 | 21.438 | 28.000 | 21.438 | 36.000 | 15.750 | 13.500 | 21.438 | 15.750 | 15.750 |
| **6** | 43.75 | 90 | 122.500 | 90.00 | 81.0000 | 56.2500 | 62.50 | 90.00 | 95.0625 | 56.250 | 90.00 | 90.0000 | 44.000 | 72.0000 | 90.000 | 90.0000 | 56.250 | 30.375 | 40.000 | 83.188 | 50.000 | 60.500 | 28.000 | 43.750 | 40.000 | 60.500 | 40.500 | 56.250 | 50.000 | 18.375 | 68.750 | 62.500 | 62.500 | 56.250 | 62.500 | 122.500 | 32.000 | 50.000 | 36.000 | 68.750 |
| **8** | 60.50 | 147 |  | 168.75 | 122.5000 | 154.6875 | 126.75 | 183.75 | 134.7500 | 147.875 | 117.00 | 108.0000 | 87.500 | 126.0000 | 126.750 | 208.0000 | 81.250 | 90.000 | 56.250 | 99.000 | 108.000 | 117.000 | 134.750 | 75.625 | 81.250 | 75.000 | 81.250 | 90.750 | 134.750 | 62.500 | 108.000 | 116.188 | 83.188 | 126.750 | 108.000 | 198.688 | 44.000 | 108.000 | 44.000 | 99.000 |
| **10** | 0.00* | 0* | 0.000* | 0.00* | 147.0000 | 192.0000 | 126.75 | 0.00* | 0.0000* | 0.000* | 0.00* | 0.0000* | 0.000* | 99.0000 | 62.500 | 122.5000 | 40.000 | 0.000* | 68.750 | 68.750 | 0.000* | 83.188 | 90.000 | 75.625 | 0.000* | 68.750 | 68.750 | 83.188 | 90.000 | 56.250 | 50.625 | 55.688 | 0.000* | 75.625 | 83.188 | 234.813 | 0.000* | 116.188 | 32.000 | 90.750 |
| **12** | 0.00* | 0* | 0.000* | 0.00* | 0.0000* | 0.0000* | 0.00* | 0.00* | 0.0000* | 0.000* | 0.00* | 0.0000* | 0.000* | 0.0000* | 0.000* | 81.0000 | 13.500 | 0.000* | 24.500 | 0.000* | 0.000* | 0.000* | 35.438 | 0.000* | 0.000* | 0.000* | 0.000* | 32.000 | 0.000* | 95.063 | 0.000* | 0.000* | 0.000* | 40.500 | 0.000* | 0.000* | 0.000* | 0.000* | 13.500 | 0.000* |
| **14** | 0.00* | 0* | 0.000* | 0.00* | 0.0000* | 0.0000* | 0.00* | 0.00* | 0.0000* | 0.000* | 0.00* | 0.0000* | 0.000* | 0.0000* | 0.000* | 50.0000 | 13.500 | 0.000* | 15.750 | 0.000* | 0.000* | 0.000* | 28.000 | 0.000* | 0.000* | 0.000* | 0.000* | 36.000 | 0.000* | 72.000 | 0.000* | 0.000* | 0.000* | 13.500 | 0.000* | 0.000* | 0.000* | 0.000* | 9.375 | 0.000* |
| **16** | 0.00* | 0* | 0.000* | 0.00* | 0.0000* | 0.0000* | 0.00* | 0.00* | 0.0000* | 0.000* | 0.00* | 0.0000* | 0.000* | 0.0000* | 0.000* | 50.0000 | 11.250 | 0.000* | 9.375 | 0.000* | 0.000* | 0.000* | 15.750 | 0.000* | 0.000* | 0.000* | 0.000* | 28.000 | 0.000* | 50.000 | 0.000* | 0.000* | 0.000* | 11.250 | 0.000* | 0.000* | 0.000* | 0.000* | 7.813 | 0.000* |
| **18** | 0.00* | 0* | 0.000* | 0.00* | 0.0000* | 0.0000* | 0.00* | 0.00* | 0.0000* | 0.000* | 0.00* | 0.0000* | 0.000* | 0.0000* | 0.000* | 24.0000 | 4.000 | 0.000* | 7.813 | 0.000* | 0.000* | 0.000* | 9.375 | 0.000* | 0.000* | 0.000* | 0.000* | 13.500 | 0.000* | 56.250 | 0.000* | 0.000* | 0.000* | 2.250 | 0.000* | 0.000* | 0.000* | 0.000* | 4.000 | 0.000* |

|  | **Days** | **Control** | **Anti PD-1 + tdRT** | **Anti PD-1 + Anti CD47** | **Anti PD-1 + Anti CD47 + tdRT** |
| --- | --- | --- | --- | --- | --- |
| **Cntrl_1** | 10 | 1 |  |  |  |
| **Cntrl_2** | 10 | 1 |  |  |  |
| **Cntrl_3** | 8 | 1 |  |  |  |
| **Cntrl_4** | 10 | 1 |  |  |  |
| **Cntrl_5** | 12 | 1 |  |  |  |
| **Cntrl_6** | 12 | 1 |  |  |  |
| **Cntrl_7** | 12 | 1 |  |  |  |
| **Cntrl_8** | 10 | 1 |  |  |  |
| **Cntrl_9** | 10 | 1 |  |  |  |
| **Cntrl_10** | 10 | 1 |  |  |  |
| **PT_1** | 10 |  | 1 |  |  |
| **PT_2** | 10 |  | 1 |  |  |
| **PT_3** | 10 |  | 1 |  |  |
| **PT_4** | 12 |  | 1 |  |  |
| **PT_5** | 12 |  | 1 |  |  |
| **PT_6** | 18 |  | 0 |  |  |
| **PT_7** | 18 |  | 0 |  |  |
| **PT_8** | 10 |  | 1 |  |  |
| **PT_9** | 18 |  | 0 |  |  |
| **PT_10** | 12 |  | 1 |  |  |
| **PC_1** | 10 |  |  | 1 |  |
| **PC_2** | 12 |  |  | 1 |  |
| **PC_3** | 18 |  |  | 0 |  |
| **PC_4** | 12 |  |  | 1 |  |
| **PC_5** | 10 |  |  | 1 |  |
| **PC_6** | 12 |  |  | 1 |  |
| **PC_7** | 12 |  |  | 1 |  |
| **PC_8** | 18 |  |  | 0 |  |
| **PC_9** | 12 |  |  | 1 |  |
| **PC_10** | 18 |  |  | 0 |  |
| **PCT_1** | 12 |  |  |  | 1 |
| **PCT_2** | 12 |  |  |  | 1 |
| **PCT_3** | 10 |  |  |  | 1 |
| **PCT_4** | 18 |  |  |  | 0 |
| **PCT_5** | 12 |  |  |  | 1 |
| **PCT_6** | 12 |  |  |  | 1 |
| **PCT_7** | 10 |  |  |  | 1 |
| **PCT_8** | 12 |  |  |  | 1 |
| **PCT_9** | 18 |  |  |  | 0 |
| **PCT_10** | 12 |  |  |  | 1 |

| **Comparison of Survival Curves** |  |
| --- | --- |
|  |  |
| **Log-rank (Mantel-Cox) test (recommended)** |  |
| **Chi square** | 8.494 |
| **df** | 3 |
| **P value** | 0.0368 |
| **P value summary** | * |
| **Are the survival curves sig different?** | Yes |
|  |  |
| **Logrank test for trend (recommended)** |  |
| **Chi square** | 4.755 |
| **df** | 1 |
| **P value** | 0.0292 |
| **P value summary** | * |
| **Sig. trend?** | Yes |
|  |  |
| **Gehan-Breslow-Wilcoxon test** |  |
| **Chi square** | 8.673 |
| **df** | 3 |
| **P value** | 0.0340 |
| **P value summary** | * |
| **Are the survival curves sig different?** | Yes |

|  | **Control** | **Anti PD-1 + tdRT** | **Anti PD-1 + Anti CD47** | **Anti PD-1 + Anti CD47 + tdRT** |
| --- | --- | --- | --- | --- |
| **Number of rows** | 40 | 40 | 40 | 40 |
| **# of blank rows** | 30 | 30 | 30 | 30 |
| **# rows with impossible data** | 0 | 0 | 0 | 0 |
| **# censored subjects** | 0 | 3 | 3 | 2 |
| **# deaths/events** | 10 | 7 | 7 | 8 |
|  |  |  |  |  |
| **Median survival** | 10 | 12 | 12 | 12 |

| **Days** | **Control** | **Anti PD-1 + tdRT** | **Anti PD-1 + Anti CD47** | **Anti PD-1 + Anti CD47 + tdRT** |
| --- | --- | --- | --- | --- |
| **0.000** | 10 | 10 | 10 | 10 |
| **8.000** | 10 |  |  |  |
| **10.000** | 9 | 10 | 10 | 10 |
| **12.000** | 3 | 6 | 8 | 8 |
| **18.000** |  | 3 | 3 | 2 |

**Supplemental Figure 1D**

| **Days Post Transplant** | **Control** | **Control** | **Control** | **anti-CD47** | **anti-CD47** | **anti-CD47** |
| --- | --- | --- | --- | --- | --- | --- |
| **0** | 0.0000000 | 0.000000000 | 8 | 0.00000000 | 0.000000000 | 7 |
| **4** | 23.1015625 | 3.452240015 | 8 | 12.64285714 | 3.839528943 | 7 |
| **6** | 66.3593750 | 9.467078738 | 8 | 50.58214286 | 10.695622690 | 7 |
| **8** | 50.5546875 | 6.197823592 | 8 | 43.66964286 | 6.886941326 | 7 |
| **10** | 49.0982500 | 5.630138000 | 8 | 45.00892857 | 7.129188000 | 7 |
| **12** | 48.9895000 | 8.033183000 | 8 | 48.26785714 | 9.594004000 | 7 |
| **14** | 49.7265625 | 9.194534519 | 8 | 41.89642857 | 6.973193348 | 7 |

| **Table Analyzed** | **Data 1** |
| --- | --- |
|  |  |
| **Column B** | anti-CD47 |
| **vs.** | vs. |
| **Column A** | Control |
|  |  |
| **Unpaired t test** |  |
| **P value** | 0.5716 |
| **P value summary** | ns |
| **Significantly different (P < 0.05)?** | No |
| **One- or two-tailed P value?** | Two-tailed |
| **t, df** | t=0.5816, df=12 |
|  |  |
| **How big is the difference?** |  |
| **Mean of column A** | 41.12 |
| **Mean of column B** | 34.58 |
| **Difference between means (B - A) ± SEM** | -6.537 ± 11.24 |
| **95% confidence interval** | -31.03 to 17.95 |
| **R squared (eta squared)** | 0.02742 |
|  |  |
| **F test to compare variances** |  |
| **F, DFn, Dfd** | 1.243, 6, 6 |
| **P value** | 0.7981 |
| **P value summary** | ns |
| **Significantly different (P < 0.05)?** | No |
|  |  |
| **Data analyzed** |  |
| **Sample size, column A** | 7 |
| **Sample size, column B** | 7 |

**Supplemental Figure 1E**

| **Days** | **Control** | **Control** | **Control** | **Control** | **Control** | **Control** | **Control** | **Control** | **Control** | **Control** | **PD1+tdRT** | **PD1+tdRT** | **PD1+tdRT** | **PD1+tdRT** | **PD1+tdRT** | **PD1+tdRT** | **PD1+tdRT** | **PD1+tdRT** | **PD1+tdRT** | **PD1+tdRT** | **PD1+CD47** | **PD1+CD47** | **PD1+CD47** | **PD1+CD47** | **PD1+CD47** | **PD1+CD47** | **PD1+CD47** | **PD1+CD47** | **PD1+CD47** | **PD1+CD47** | **PD1+CD47+tdRT** | **PD1+CD47+tdRT** | **PD1+CD47+tdRT** | **PD1+CD47+tdRT** | **PD1+CD47+tdRT** | **PD1+CD47+tdRT** | **PD1+CD47+tdRT** | **PD1+CD47+tdRT** | **PD1+CD47+tdRT** | **PD1+CD47+tdRT** |
| --- | --- | --- | --- | --- | --- | --- | --- | --- | --- | --- | --- | --- | --- | --- | --- | --- | --- | --- | --- | --- | --- | --- | --- | --- | --- | --- | --- | --- | --- | --- | --- | --- | --- | --- | --- | --- | --- | --- | --- | --- |
| **0** | 0.0000 | 0.00000 | 0.000 | 0.00000000 | 0.0000 | 0.00 | 0.000 | 0.00 | 0.0000 | 0.0000 | 0.00 | 0.00 | 0.0000 | 0.00 | 0.0000 | 0.0 | 0.0000 | 0.0000 | 0.00 | 0.00 | 0.0 | 0.0 | 0.000 | 0.0000 | 0.0000 | 0.0000 | 0.0000 | 0.000 | 0.0000 | 0.0000 | 0.0000 | 0.0000 | 0.0000 | 0.0000 | 0.000 | 0.0000 | 0 | 0.00 | 0.0000 | 0.0000 |
| **4** | 13.5000 | 13.50000 | 15.750 | 15.75000000 | 21.4375 | 15.75 | 18.000 | 18.00 | 18.3750 | 15.7500 | 15.75 | 13.50 | 24.5000 | 13.50 | 21.4375 | 13.5 | 18.0000 | 18.0000 | 15.75 | 15.75 | 13.5 | 13.5 | 13.500 | 21.4375 | 32.0000 | 21.4375 | 28.0000 | 15.750 | 15.7500 | 13.5000 | 21.4375 | 21.4375 | 21.4375 | 15.7500 | 15.750 | 15.7500 | 18 | 24.50 | 15.7500 | 18.0000 |
| **6** | 24.5000 | 62.50000 | 24.500 | 36.00000000 | 24.5000 | 20.25 | 40.000 | 60.75 | 33.6875 | 45.5625 | 36.00 | 24.50 | 45.5625 | 28.00 | 40.0000 | 40.0 | 36.0000 | 45.5625 | 36.00 | 32.00 | 32.0 | 32.0 | 24.500 | 36.0000 | 55.6875 | 45.5625 | 36.0000 | 50.625 | 27.5625 | 45.5625 | 36.0000 | 36.0000 | 36.0000 | 24.5000 | 50.625 | 27.5625 | 36 | 32.00 | 21.4375 | 27.5625 |
| **8** | 83.1875 |  | 68.750 | 68.75000000 | 105.8750 | 87.50 | 105.875 | 90.75 | 113.4375 | 99.0000 | 60.75 | 81.25 | 105.8750 | 68.75 | 55.6875 | 48.0 | 98.3125 | 117.0000 | 68.75 | 75.00 | 62.5 | 62.5 | 50.625 | 98.3125 | 117.0000 | 98.3125 | 98.3125 | 90.750 | 81.2500 | 68.7500 | 81.2500 | 81.2500 | 105.8750 | 98.3125 | 81.250 | 55.6875 | 75 | 68.75 | 55.6875 | 81.2500 |

| **Within each row, compare columns (simple effects within rows)** |  |  |  |  |  |  |  |  |
| --- | --- | --- | --- | --- | --- | --- | --- | --- |
|  |  |  |  |  |  |  |  |  |
| **Number of families** | 4 |  |  |  |  |  |  |  |
| **Number of comparisons per family** | 6 |  |  |  |  |  |  |  |
| **Alpha** | 0.05 |  |  |  |  |  |  |  |
|  |  |  |  |  |  |  |  |  |
| **Tukey's multiple comparisons test** | Predicted (LS) mean diff. | 95.00% CI of diff. | Below threshold? | Summary | Adjusted P Value |  |  |  |
|  |  |  |  |  |  |  |  |  |
| **Row 1** |  |  |  |  |  |  |  |  |
| **Control vs. PD1+tdRT** | 0.000 | -12.94 to 12.94 | No | ns | >0.9999 |  |  |  |
| **Control vs. PD1+CD47** | 0.000 | -12.94 to 12.94 | No | ns | >0.9999 |  |  |  |
| **Control vs. PD1+CD47+tdRT** | 0.000 | -12.94 to 12.94 | No | ns | >0.9999 |  |  |  |
| **PD1+tdRT vs. PD1+CD47** | 0.000 | -12.94 to 12.94 | No | ns | >0.9999 |  |  |  |
| **PD1+tdRT vs. PD1+CD47+tdRT** | 0.000 | -12.94 to 12.94 | No | ns | >0.9999 |  |  |  |
| **PD1+CD47 vs. PD1+CD47+tdRT** | 0.000 | -12.94 to 12.94 | No | ns | >0.9999 |  |  |  |
|  |  |  |  |  |  |  |  |  |
| **Row 2** |  |  |  |  |  |  |  |  |
| **Control vs. PD1+tdRT** | -0.3875 | -13.33 to 12.55 | No | ns | 0.9998 |  |  |  |
| **Control vs. PD1+CD47** | -2.256 | -15.20 to 10.68 | No | ns | 0.9689 |  |  |  |
| **Control vs. PD1+CD47+tdRT** | -2.200 | -15.14 to 10.74 | No | ns | 0.9711 |  |  |  |
| **PD1+tdRT vs. PD1+CD47** | -1.869 | -14.81 to 11.07 | No | ns | 0.9819 |  |  |  |
| **PD1+tdRT vs. PD1+CD47+tdRT** | -1.813 | -14.75 to 11.13 | No | ns | 0.9834 |  |  |  |
| **PD1+CD47 vs. PD1+CD47+tdRT** | 0.05625 | -12.88 to 13.00 | No | ns | >0.9999 |  |  |  |
|  |  |  |  |  |  |  |  |  |
| **Row 3** |  |  |  |  |  |  |  |  |
| **Control vs. PD1+tdRT** | 0.8625 | -12.08 to 13.80 | No | ns | 0.9981 |  |  |  |
| **Control vs. PD1+CD47** | -1.325 | -14.27 to 11.62 | No | ns | 0.9934 |  |  |  |
| **Control vs. PD1+CD47+tdRT** | 4.456 | -8.485 to 17.40 | No | ns | 0.8074 |  |  |  |
| **PD1+tdRT vs. PD1+CD47** | -2.187 | -15.13 to 10.75 | No | ns | 0.9715 |  |  |  |
| **PD1+tdRT vs. PD1+CD47+tdRT** | 3.594 | -9.347 to 16.53 | No | ns | 0.8882 |  |  |  |
| **PD1+CD47 vs. PD1+CD47+tdRT** | 5.781 | -7.160 to 18.72 | No | ns | 0.6522 |  |  |  |
|  |  |  |  |  |  |  |  |  |
| **Row 4** |  |  |  |  |  |  |  |  |
| **Control vs. PD1+tdRT** | 13.52 | 0.2251 to 26.82 | Yes | * | 0.0446 |  |  |  |
| **Control vs. PD1+CD47** | 8.627 | -4.669 to 21.92 | No | ns | 0.3345 |  |  |  |
| **Control vs. PD1+CD47+tdRT** | 13.03 | -0.2686 to 26.32 | No | ns | 0.0571 |  |  |  |
| **PD1+tdRT vs. PD1+CD47** | -4.894 | -17.83 to 8.047 | No | ns | 0.7593 |  |  |  |
| **PD1+tdRT vs. PD1+CD47+tdRT** | -0.4938 | -13.43 to 12.45 | No | ns | 0.9996 |  |  |  |
| **PD1+CD47 vs. PD1+CD47+tdRT** | 4.400 | -8.541 to 17.34 | No | ns | 0.8133 |  |  |  |
|  |  |  |  |  |  |  |  |  |
|  |  |  |  |  |  |  |  |  |
| **Test details** | Predicted (LS) mean 1 | Predicted (LS) mean 2 | Predicted (LS) mean diff. | SE of diff. | N1 | N2 | q | DF |
|  |  |  |  |  |  |  |  |  |
| **Row 1** |  |  |  |  |  |  |  |  |
| **Control vs. PD1+tdRT** | 0.000 | 0.000 | 0.000 | 4.978 | 10 | 10 | 0.000 | 143.0 |
| **Control vs. PD1+CD47** | 0.000 | 0.000 | 0.000 | 4.978 | 10 | 10 | 0.000 | 143.0 |
| **Control vs. PD1+CD47+tdRT** | 0.000 | 0.000 | 0.000 | 4.978 | 10 | 10 | 0.000 | 143.0 |
| **PD1+tdRT vs. PD1+CD47** | 0.000 | 0.000 | 0.000 | 4.978 | 10 | 10 | 0.000 | 143.0 |
| **PD1+tdRT vs. PD1+CD47+tdRT** | 0.000 | 0.000 | 0.000 | 4.978 | 10 | 10 | 0.000 | 143.0 |
| **PD1+CD47 vs. PD1+CD47+tdRT** | 0.000 | 0.000 | 0.000 | 4.978 | 10 | 10 | 0.000 | 143.0 |
|  |  |  |  |  |  |  |  |  |
| **Row 2** |  |  |  |  |  |  |  |  |
| **Control vs. PD1+tdRT** | 16.58 | 16.97 | -0.3875 | 4.978 | 10 | 10 | 0.1101 | 143.0 |
| **Control vs. PD1+CD47** | 16.58 | 18.84 | -2.256 | 4.978 | 10 | 10 | 0.6409 | 143.0 |
| **Control vs. PD1+CD47+tdRT** | 16.58 | 18.78 | -2.200 | 4.978 | 10 | 10 | 0.6250 | 143.0 |
| **PD1+tdRT vs. PD1+CD47** | 16.97 | 18.84 | -1.869 | 4.978 | 10 | 10 | 0.5309 | 143.0 |
| **PD1+tdRT vs. PD1+CD47+tdRT** | 16.97 | 18.78 | -1.813 | 4.978 | 10 | 10 | 0.5149 | 143.0 |
| **PD1+CD47 vs. PD1+CD47+tdRT** | 18.84 | 18.78 | 0.05625 | 4.978 | 10 | 10 | 0.01598 | 143.0 |
|  |  |  |  |  |  |  |  |  |
| **Row 3** |  |  |  |  |  |  |  |  |
| **Control vs. PD1+tdRT** | 37.23 | 36.36 | 0.8625 | 4.978 | 10 | 10 | 0.2450 | 143.0 |
| **Control vs. PD1+CD47** | 37.23 | 38.55 | -1.325 | 4.978 | 10 | 10 | 0.3764 | 143.0 |
| **Control vs. PD1+CD47+tdRT** | 37.23 | 32.77 | 4.456 | 4.978 | 10 | 10 | 1.266 | 143.0 |
| **PD1+tdRT vs. PD1+CD47** | 36.36 | 38.55 | -2.187 | 4.978 | 10 | 10 | 0.6214 | 143.0 |
| **PD1+tdRT vs. PD1+CD47+tdRT** | 36.36 | 32.77 | 3.594 | 4.978 | 10 | 10 | 1.021 | 143.0 |
| **PD1+CD47 vs. PD1+CD47+tdRT** | 38.55 | 32.77 | 5.781 | 4.978 | 10 | 10 | 1.642 | 143.0 |
|  |  |  |  |  |  |  |  |  |
| **Row 4** |  |  |  |  |  |  |  |  |
| **Control vs. PD1+tdRT** | 91.46 | 77.94 | 13.52 | 5.115 | 9 | 10 | 3.738 | 143.0 |
| **Control vs. PD1+CD47** | 91.46 | 82.83 | 8.627 | 5.115 | 9 | 10 | 2.385 | 143.0 |
| **Control vs. PD1+CD47+tdRT** | 91.46 | 78.43 | 13.03 | 5.115 | 9 | 10 | 3.602 | 143.0 |
| **PD1+tdRT vs. PD1+CD47** | 77.94 | 82.83 | -4.894 | 4.978 | 10 | 10 | 1.390 | 143.0 |
| **PD1+tdRT vs. PD1+CD47+tdRT** | 77.94 | 78.43 | -0.4938 | 4.978 | 10 | 10 | 0.1403 | 143.0 |
| **PD1+CD47 vs. PD1+CD47+tdRT** | 82.83 | 78.43 | 4.400 | 4.978 | 10 | 10 | 1.250 | 143.0 |

**Supplemental Figure 1F**

| **Days Post Transplant** | **Control** | **Control** | **Control** | **CD47** | **CD47** | **CD47** | **PD1** | **PD1** | **PD1** | **CD47 + PD1** | **CD47 + PD1** | **CD47 + PD1** | **PD1 + tdRT** | **PD1 + tdRT** | **PD1 + tdRT** | **CD47 + PD1 + tdRT** | **CD47 + PD1 + tdRT** | **CD47 + PD1 + tdRT** |
| --- | --- | --- | --- | --- | --- | --- | --- | --- | --- | --- | --- | --- | --- | --- | --- | --- | --- | --- |
| **0** | 0.00000 | 0.000000000 | 10 | 0.0000000 | 0.000000000 | 8 | 0.0000000 | 0.000000000 | 8 | 0.0000000 | 0.000000000 | 8 | 0.0000000 | 0.000000000 | 8 | 0.0000000 | 0.000000000 | 8 |
| **4** | 23.62500 | 3.158186287 | 10 | 33.5390625 | 5.385991413 | 8 | 23.9843750 | 3.636578189 | 8 | 17.6718750 | 4.641910465 | 8 | 31.7968750 | 5.288638392 | 8 | 23.8203125 | 4.728059390 | 8 |
| **6** | 56.85000 | 9.673494715 | 10 | 112.2031250 | 18.181564990 | 8 | 72.8671875 | 14.878521930 | 8 | 57.7265625 | 8.419308641 | 8 | 95.2578125 | 12.207327810 | 8 | 74.8828125 | 11.781677080 | 8 |
| **8** | 90.75000 | 13.204742870 | 10 | 101.9531250 | 15.164828230 | 8 | 101.2500000 | 11.070177420 | 8 | 91.0781250 | 13.374323460 | 8 | 123.1562500 | 13.713939750 | 8 | 94.5468750 | 13.325000320 | 8 |
| **9** | 89.46875 | 16.675800000 | 10 | 105.8515625 | 11.320560000 | 8 | 119.7031250 | 18.894650000 | 8 | 89.5781250 | 13.302570000 | 8 | 128.1484375 | 15.849300000 | 8 | 107.0859000 | 10.558040000 | 8 |

| **Within each row, compare columns (simple effects within rows)** |  |  |  |  |  |  |  |  |
| --- | --- | --- | --- | --- | --- | --- | --- | --- |
|  |  |  |  |  |  |  |  |  |
| **Number of families** | 5 |  |  |  |  |  |  |  |
| **Number of comparisons per family** | 15 |  |  |  |  |  |  |  |
| **Alpha** | 0.05 |  |  |  |  |  |  |  |
|  |  |  |  |  |  |  |  |  |
| **Tukey's multiple comparisons test** | Predicted (LS) mean diff. | 95.00% CI of diff. | Below threshold? | Summary | Adjusted P Value |  |  |  |
|  |  |  |  |  |  |  |  |  |
| **Row 1** |  |  |  |  |  |  |  |  |
| **Control vs. CD47** | 0.000 | -42.64 to 42.64 | No | ns | >0.9999 |  |  |  |
| **Control vs. PD1** | 0.000 | -42.64 to 42.64 | No | ns | >0.9999 |  |  |  |
| **Control vs. CD47 + PD1** | 0.000 | -42.64 to 42.64 | No | ns | >0.9999 |  |  |  |
| **Control vs. PD1 + tdRT** | 0.000 | -42.64 to 42.64 | No | ns | >0.9999 |  |  |  |
| **Control vs. CD47 + PD1 + tdRT** | 0.000 | -42.64 to 42.64 | No | ns | >0.9999 |  |  |  |
| **CD47 vs. PD1** | 0.000 | -44.94 to 44.94 | No | ns | >0.9999 |  |  |  |
| **CD47 vs. CD47 + PD1** | 0.000 | -44.94 to 44.94 | No | ns | >0.9999 |  |  |  |
| **CD47 vs. PD1 + tdRT** | 0.000 | -44.94 to 44.94 | No | ns | >0.9999 |  |  |  |
| **CD47 vs. CD47 + PD1 + tdRT** | 0.000 | -44.94 to 44.94 | No | ns | >0.9999 |  |  |  |
| **PD1 vs. CD47 + PD1** | 0.000 | -44.94 to 44.94 | No | ns | >0.9999 |  |  |  |
| **PD1 vs. PD1 + tdRT** | 0.000 | -44.94 to 44.94 | No | ns | >0.9999 |  |  |  |
| **PD1 vs. CD47 + PD1 + tdRT** | 0.000 | -44.94 to 44.94 | No | ns | >0.9999 |  |  |  |
| **CD47 + PD1 vs. PD1 + tdRT** | 0.000 | -44.94 to 44.94 | No | ns | >0.9999 |  |  |  |
| **CD47 + PD1 vs. CD47 + PD1 + tdRT** | 0.000 | -44.94 to 44.94 | No | ns | >0.9999 |  |  |  |
| **PD1 + tdRT vs. CD47 + PD1 + tdRT** | 0.000 | -44.94 to 44.94 | No | ns | >0.9999 |  |  |  |
|  |  |  |  |  |  |  |  |  |
| **Row 2** |  |  |  |  |  |  |  |  |
| **Control vs. CD47** | -9.914 | -52.55 to 32.72 | No | ns | 0.9852 |  |  |  |
| **Control vs. PD1** | -0.3594 | -43.00 to 42.28 | No | ns | >0.9999 |  |  |  |
| **Control vs. CD47 + PD1** | 5.953 | -36.68 to 48.59 | No | ns | 0.9986 |  |  |  |
| **Control vs. PD1 + tdRT** | -8.172 | -50.81 to 34.47 | No | ns | 0.9939 |  |  |  |
| **Control vs. CD47 + PD1 + tdRT** | -0.1953 | -42.83 to 42.44 | No | ns | >0.9999 |  |  |  |
| **CD47 vs. PD1** | 9.555 | -35.39 to 54.50 | No | ns | 0.9901 |  |  |  |
| **CD47 vs. CD47 + PD1** | 15.87 | -29.08 to 60.81 | No | ns | 0.9126 |  |  |  |
| **CD47 vs. PD1 + tdRT** | 1.742 | -43.20 to 46.69 | No | ns | >0.9999 |  |  |  |
| **CD47 vs. CD47 + PD1 + tdRT** | 9.719 | -35.22 to 54.66 | No | ns | 0.9893 |  |  |  |
| **PD1 vs. CD47 + PD1** | 6.312 | -38.63 to 51.26 | No | ns | 0.9986 |  |  |  |
| **PD1 vs. PD1 + tdRT** | -7.813 | -52.76 to 37.13 | No | ns | 0.9961 |  |  |  |
| **PD1 vs. CD47 + PD1 + tdRT** | 0.1641 | -44.78 to 45.11 | No | ns | >0.9999 |  |  |  |
| **CD47 + PD1 vs. PD1 + tdRT** | -14.13 | -59.07 to 30.82 | No | ns | 0.9453 |  |  |  |
| **CD47 + PD1 vs. CD47 + PD1 + tdRT** | -6.148 | -51.09 to 38.79 | No | ns | 0.9988 |  |  |  |
| **PD1 + tdRT vs. CD47 + PD1 + tdRT** | 7.977 | -36.97 to 52.92 | No | ns | 0.9957 |  |  |  |
|  |  |  |  |  |  |  |  |  |
| **Row 3** |  |  |  |  |  |  |  |  |
| **Control vs. CD47** | -55.35 | -97.99 to -12.72 | Yes | ** | 0.0033 |  |  |  |
| **Control vs. PD1** | -16.02 | -58.65 to 26.62 | No | ns | 0.8889 |  |  |  |
| **Control vs. CD47 + PD1** | -0.8766 | -43.51 to 41.76 | No | ns | >0.9999 |  |  |  |
| **Control vs. PD1 + tdRT** | -38.41 | -81.04 to 4.229 | No | ns | 0.1041 |  |  |  |
| **Control vs. CD47 + PD1 + tdRT** | -18.03 | -60.67 to 24.60 | No | ns | 0.8287 |  |  |  |
| **CD47 vs. PD1** | 39.34 | -5.607 to 84.28 | No | ns | 0.1238 |  |  |  |
| **CD47 vs. CD47 + PD1** | 54.48 | 9.533 to 99.42 | Yes | ** | 0.0077 |  |  |  |
| **CD47 vs. PD1 + tdRT** | 16.95 | -28.00 to 61.89 | No | ns | 0.8874 |  |  |  |
| **CD47 vs. CD47 + PD1 + tdRT** | 37.32 | -7.623 to 82.26 | No | ns | 0.1652 |  |  |  |
| **PD1 vs. CD47 + PD1** | 15.14 | -29.80 to 60.08 | No | ns | 0.9274 |  |  |  |
| **PD1 vs. PD1 + tdRT** | -22.39 | -67.33 to 22.55 | No | ns | 0.7073 |  |  |  |
| **PD1 vs. CD47 + PD1 + tdRT** | -2.016 | -46.96 to 42.93 | No | ns | >0.9999 |  |  |  |
| **CD47 + PD1 vs. PD1 + tdRT** | -37.53 | -82.47 to 7.412 | No | ns | 0.1604 |  |  |  |
| **CD47 + PD1 vs. CD47 + PD1 + tdRT** | -17.16 | -62.10 to 27.79 | No | ns | 0.8820 |  |  |  |
| **PD1 + tdRT vs. CD47 + PD1 + tdRT** | 20.38 | -24.57 to 65.32 | No | ns | 0.7830 |  |  |  |
|  |  |  |  |  |  |  |  |  |
| **Row 4** |  |  |  |  |  |  |  |  |
| **Control vs. CD47** | -11.20 | -53.84 to 31.43 | No | ns | 0.9745 |  |  |  |
| **Control vs. PD1** | -10.50 | -53.14 to 32.14 | No | ns | 0.9808 |  |  |  |
| **Control vs. CD47 + PD1** | -0.3281 | -42.97 to 42.31 | No | ns | >0.9999 |  |  |  |
| **Control vs. PD1 + tdRT** | -32.41 | -75.04 to 10.23 | No | ns | 0.2489 |  |  |  |
| **Control vs. CD47 + PD1 + tdRT** | -3.797 | -46.43 to 38.84 | No | ns | 0.9998 |  |  |  |
| **CD47 vs. PD1** | 0.7031 | -44.24 to 45.65 | No | ns | >0.9999 |  |  |  |
| **CD47 vs. CD47 + PD1** | 10.88 | -34.07 to 55.82 | No | ns | 0.9823 |  |  |  |
| **CD47 vs. PD1 + tdRT** | -21.20 | -66.15 to 23.74 | No | ns | 0.7529 |  |  |  |
| **CD47 vs. CD47 + PD1 + tdRT** | 7.406 | -37.54 to 52.35 | No | ns | 0.9970 |  |  |  |
| **PD1 vs. CD47 + PD1** | 10.17 | -34.77 to 55.12 | No | ns | 0.9869 |  |  |  |
| **PD1 vs. PD1 + tdRT** | -21.91 | -66.85 to 23.04 | No | ns | 0.7262 |  |  |  |
| **PD1 vs. CD47 + PD1 + tdRT** | 6.703 | -38.24 to 51.65 | No | ns | 0.9981 |  |  |  |
| **CD47 + PD1 vs. PD1 + tdRT** | -32.08 | -77.02 to 12.87 | No | ns | 0.3166 |  |  |  |
| **CD47 + PD1 vs. CD47 + PD1 + tdRT** | -3.469 | -48.41 to 41.47 | No | ns | >0.9999 |  |  |  |
| **PD1 + tdRT vs. CD47 + PD1 + tdRT** | 28.61 | -16.33 to 73.55 | No | ns | 0.4486 |  |  |  |
|  |  |  |  |  |  |  |  |  |
| **Row 5** |  |  |  |  |  |  |  |  |
| **Control vs. CD47** | -16.38 | -59.02 to 26.25 | No | ns | 0.8791 |  |  |  |
| **Control vs. PD1** | -30.23 | -72.87 to 12.40 | No | ns | 0.3239 |  |  |  |
| **Control vs. CD47 + PD1** | -0.1094 | -42.75 to 42.53 | No | ns | >0.9999 |  |  |  |
| **Control vs. PD1 + tdRT** | -38.68 | -81.32 to 3.957 | No | ns | 0.0996 |  |  |  |
| **Control vs. CD47 + PD1 + tdRT** | -17.62 | -60.25 to 25.02 | No | ns | 0.8423 |  |  |  |
| **CD47 vs. PD1** | -13.85 | -58.79 to 31.09 | No | ns | 0.9495 |  |  |  |
| **CD47 vs. CD47 + PD1** | 16.27 | -28.67 to 61.22 | No | ns | 0.9035 |  |  |  |
| **CD47 vs. PD1 + tdRT** | -22.30 | -67.24 to 22.65 | No | ns | 0.7110 |  |  |  |
| **CD47 vs. CD47 + PD1 + tdRT** | -1.234 | -46.18 to 43.71 | No | ns | >0.9999 |  |  |  |
| **PD1 vs. CD47 + PD1** | 30.13 | -14.82 to 75.07 | No | ns | 0.3884 |  |  |  |
| **PD1 vs. PD1 + tdRT** | -8.445 | -53.39 to 36.50 | No | ns | 0.9944 |  |  |  |
| **PD1 vs. CD47 + PD1 + tdRT** | 12.62 | -32.33 to 57.56 | No | ns | 0.9660 |  |  |  |
| **CD47 + PD1 vs. PD1 + tdRT** | -38.57 | -83.51 to 6.373 | No | ns | 0.1385 |  |  |  |
| **CD47 + PD1 vs. CD47 + PD1 + tdRT** | -17.51 | -62.45 to 27.44 | No | ns | 0.8727 |  |  |  |
| **PD1 + tdRT vs. CD47 + PD1 + tdRT** | 21.06 | -23.88 to 66.01 | No | ns | 0.7581 |  |  |  |
|  |  |  |  |  |  |  |  |  |
|  |  |  |  |  |  |  |  |  |
| **Test details** | Predicted (LS) mean 1 | Predicted (LS) mean 2 | Predicted (LS) mean diff. | SE of diff. | N1 | N2 | q | DF |
|  |  |  |  |  |  |  |  |  |
| **Row 1** |  |  |  |  |  |  |  |  |
| **Control vs. CD47** | 0.000 | 0.000 | 0.000 | 14.83 | 10 | 8 | 0.000 | 220.0 |
| **Control vs. PD1** | 0.000 | 0.000 | 0.000 | 14.83 | 10 | 8 | 0.000 | 220.0 |
| **Control vs. CD47 + PD1** | 0.000 | 0.000 | 0.000 | 14.83 | 10 | 8 | 0.000 | 220.0 |
| **Control vs. PD1 + tdRT** | 0.000 | 0.000 | 0.000 | 14.83 | 10 | 8 | 0.000 | 220.0 |
| **Control vs. CD47 + PD1 + tdRT** | 0.000 | 0.000 | 0.000 | 14.83 | 10 | 8 | 0.000 | 220.0 |
| **CD47 vs. PD1** | 0.000 | 0.000 | 0.000 | 15.63 | 8 | 8 | 0.000 | 220.0 |
| **CD47 vs. CD47 + PD1** | 0.000 | 0.000 | 0.000 | 15.63 | 8 | 8 | 0.000 | 220.0 |
| **CD47 vs. PD1 + tdRT** | 0.000 | 0.000 | 0.000 | 15.63 | 8 | 8 | 0.000 | 220.0 |
| **CD47 vs. CD47 + PD1 + tdRT** | 0.000 | 0.000 | 0.000 | 15.63 | 8 | 8 | 0.000 | 220.0 |
| **PD1 vs. CD47 + PD1** | 0.000 | 0.000 | 0.000 | 15.63 | 8 | 8 | 0.000 | 220.0 |
| **PD1 vs. PD1 + tdRT** | 0.000 | 0.000 | 0.000 | 15.63 | 8 | 8 | 0.000 | 220.0 |
| **PD1 vs. CD47 + PD1 + tdRT** | 0.000 | 0.000 | 0.000 | 15.63 | 8 | 8 | 0.000 | 220.0 |
| **CD47 + PD1 vs. PD1 + tdRT** | 0.000 | 0.000 | 0.000 | 15.63 | 8 | 8 | 0.000 | 220.0 |
| **CD47 + PD1 vs. CD47 + PD1 + tdRT** | 0.000 | 0.000 | 0.000 | 15.63 | 8 | 8 | 0.000 | 220.0 |
| **PD1 + tdRT vs. CD47 + PD1 + tdRT** | 0.000 | 0.000 | 0.000 | 15.63 | 8 | 8 | 0.000 | 220.0 |
|  |  |  |  |  |  |  |  |  |
| **Row 2** |  |  |  |  |  |  |  |  |
| **Control vs. CD47** | 23.63 | 33.54 | -9.914 | 14.83 | 10 | 8 | 0.9454 | 220.0 |
| **Control vs. PD1** | 23.63 | 23.98 | -0.3594 | 14.83 | 10 | 8 | 0.03427 | 220.0 |
| **Control vs. CD47 + PD1** | 23.63 | 17.67 | 5.953 | 14.83 | 10 | 8 | 0.5677 | 220.0 |
| **Control vs. PD1 + tdRT** | 23.63 | 31.80 | -8.172 | 14.83 | 10 | 8 | 0.7793 | 220.0 |
| **Control vs. CD47 + PD1 + tdRT** | 23.63 | 23.82 | -0.1953 | 14.83 | 10 | 8 | 0.01863 | 220.0 |
| **CD47 vs. PD1** | 33.54 | 23.98 | 9.555 | 15.63 | 8 | 8 | 0.8644 | 220.0 |
| **CD47 vs. CD47 + PD1** | 33.54 | 17.67 | 15.87 | 15.63 | 8 | 8 | 1.435 | 220.0 |
| **CD47 vs. PD1 + tdRT** | 33.54 | 31.80 | 1.742 | 15.63 | 8 | 8 | 0.1576 | 220.0 |
| **CD47 vs. CD47 + PD1 + tdRT** | 33.54 | 23.82 | 9.719 | 15.63 | 8 | 8 | 0.8792 | 220.0 |
| **PD1 vs. CD47 + PD1** | 23.98 | 17.67 | 6.312 | 15.63 | 8 | 8 | 0.5711 | 220.0 |
| **PD1 vs. PD1 + tdRT** | 23.98 | 31.80 | -7.813 | 15.63 | 8 | 8 | 0.7068 | 220.0 |
| **PD1 vs. CD47 + PD1 + tdRT** | 23.98 | 23.82 | 0.1641 | 15.63 | 8 | 8 | 0.01484 | 220.0 |
| **CD47 + PD1 vs. PD1 + tdRT** | 17.67 | 31.80 | -14.13 | 15.63 | 8 | 8 | 1.278 | 220.0 |
| **CD47 + PD1 vs. CD47 + PD1 + tdRT** | 17.67 | 23.82 | -6.148 | 15.63 | 8 | 8 | 0.5562 | 220.0 |
| **PD1 + tdRT vs. CD47 + PD1 + tdRT** | 31.80 | 23.82 | 7.977 | 15.63 | 8 | 8 | 0.7216 | 220.0 |
|  |  |  |  |  |  |  |  |  |
| **Row 3** |  |  |  |  |  |  |  |  |
| **Control vs. CD47** | 56.85 | 112.2 | -55.35 | 14.83 | 10 | 8 | 5.278 | 220.0 |
| **Control vs. PD1** | 56.85 | 72.87 | -16.02 | 14.83 | 10 | 8 | 1.527 | 220.0 |
| **Control vs. CD47 + PD1** | 56.85 | 57.73 | -0.8766 | 14.83 | 10 | 8 | 0.08359 | 220.0 |
| **Control vs. PD1 + tdRT** | 56.85 | 95.26 | -38.41 | 14.83 | 10 | 8 | 3.663 | 220.0 |
| **Control vs. CD47 + PD1 + tdRT** | 56.85 | 74.88 | -18.03 | 14.83 | 10 | 8 | 1.720 | 220.0 |
| **CD47 vs. PD1** | 112.2 | 72.87 | 39.34 | 15.63 | 8 | 8 | 3.559 | 220.0 |
| **CD47 vs. CD47 + PD1** | 112.2 | 57.73 | 54.48 | 15.63 | 8 | 8 | 4.928 | 220.0 |
| **CD47 vs. PD1 + tdRT** | 112.2 | 95.26 | 16.95 | 15.63 | 8 | 8 | 1.533 | 220.0 |
| **CD47 vs. CD47 + PD1 + tdRT** | 112.2 | 74.88 | 37.32 | 15.63 | 8 | 8 | 3.376 | 220.0 |
| **PD1 vs. CD47 + PD1** | 72.87 | 57.73 | 15.14 | 15.63 | 8 | 8 | 1.370 | 220.0 |
| **PD1 vs. PD1 + tdRT** | 72.87 | 95.26 | -22.39 | 15.63 | 8 | 8 | 2.026 | 220.0 |
| **PD1 vs. CD47 + PD1 + tdRT** | 72.87 | 74.88 | -2.016 | 15.63 | 8 | 8 | 0.1823 | 220.0 |
| **CD47 + PD1 vs. PD1 + tdRT** | 57.73 | 95.26 | -37.53 | 15.63 | 8 | 8 | 3.395 | 220.0 |
| **CD47 + PD1 vs. CD47 + PD1 + tdRT** | 57.73 | 74.88 | -17.16 | 15.63 | 8 | 8 | 1.552 | 220.0 |
| **PD1 + tdRT vs. CD47 + PD1 + tdRT** | 95.26 | 74.88 | 20.38 | 15.63 | 8 | 8 | 1.843 | 220.0 |
|  |  |  |  |  |  |  |  |  |
| **Row 4** |  |  |  |  |  |  |  |  |
| **Control vs. CD47** | 90.75 | 102.0 | -11.20 | 14.83 | 10 | 8 | 1.068 | 220.0 |
| **Control vs. PD1** | 90.75 | 101.3 | -10.50 | 14.83 | 10 | 8 | 1.001 | 220.0 |
| **Control vs. CD47 + PD1** | 90.75 | 91.08 | -0.3281 | 14.83 | 10 | 8 | 0.03129 | 220.0 |
| **Control vs. PD1 + tdRT** | 90.75 | 123.2 | -32.41 | 14.83 | 10 | 8 | 3.090 | 220.0 |
| **Control vs. CD47 + PD1 + tdRT** | 90.75 | 94.55 | -3.797 | 14.83 | 10 | 8 | 0.3621 | 220.0 |
| **CD47 vs. PD1** | 102.0 | 101.3 | 0.7031 | 15.63 | 8 | 8 | 0.06361 | 220.0 |
| **CD47 vs. CD47 + PD1** | 102.0 | 91.08 | 10.88 | 15.63 | 8 | 8 | 0.9838 | 220.0 |
| **CD47 vs. PD1 + tdRT** | 102.0 | 123.2 | -21.20 | 15.63 | 8 | 8 | 1.918 | 220.0 |
| **CD47 vs. CD47 + PD1 + tdRT** | 102.0 | 94.55 | 7.406 | 15.63 | 8 | 8 | 0.6700 | 220.0 |
| **PD1 vs. CD47 + PD1** | 101.3 | 91.08 | 10.17 | 15.63 | 8 | 8 | 0.9202 | 220.0 |
| **PD1 vs. PD1 + tdRT** | 101.3 | 123.2 | -21.91 | 15.63 | 8 | 8 | 1.982 | 220.0 |
| **PD1 vs. CD47 + PD1 + tdRT** | 101.3 | 94.55 | 6.703 | 15.63 | 8 | 8 | 0.6064 | 220.0 |
| **CD47 + PD1 vs. PD1 + tdRT** | 91.08 | 123.2 | -32.08 | 15.63 | 8 | 8 | 2.902 | 220.0 |
| **CD47 + PD1 vs. CD47 + PD1 + tdRT** | 91.08 | 94.55 | -3.469 | 15.63 | 8 | 8 | 0.3138 | 220.0 |
| **PD1 + tdRT vs. CD47 + PD1 + tdRT** | 123.2 | 94.55 | 28.61 | 15.63 | 8 | 8 | 2.588 | 220.0 |
|  |  |  |  |  |  |  |  |  |
| **Row 5** |  |  |  |  |  |  |  |  |
| **Control vs. CD47** | 89.47 | 105.9 | -16.38 | 14.83 | 10 | 8 | 1.562 | 220.0 |
| **Control vs. PD1** | 89.47 | 119.7 | -30.23 | 14.83 | 10 | 8 | 2.883 | 220.0 |
| **Control vs. CD47 + PD1** | 89.47 | 89.58 | -0.1094 | 14.83 | 10 | 8 | 0.01043 | 220.0 |
| **Control vs. PD1 + tdRT** | 89.47 | 128.1 | -38.68 | 14.83 | 10 | 8 | 3.689 | 220.0 |
| **Control vs. CD47 + PD1 + tdRT** | 89.47 | 107.1 | -17.62 | 14.83 | 10 | 8 | 1.680 | 220.0 |
| **CD47 vs. PD1** | 105.9 | 119.7 | -13.85 | 15.63 | 8 | 8 | 1.253 | 220.0 |
| **CD47 vs. CD47 + PD1** | 105.9 | 89.58 | 16.27 | 15.63 | 8 | 8 | 1.472 | 220.0 |
| **CD47 vs. PD1 + tdRT** | 105.9 | 128.1 | -22.30 | 15.63 | 8 | 8 | 2.017 | 220.0 |
| **CD47 vs. CD47 + PD1 + tdRT** | 105.9 | 107.1 | -1.234 | 15.63 | 8 | 8 | 0.1117 | 220.0 |
| **PD1 vs. CD47 + PD1** | 119.7 | 89.58 | 30.13 | 15.63 | 8 | 8 | 2.725 | 220.0 |
| **PD1 vs. PD1 + tdRT** | 119.7 | 128.1 | -8.445 | 15.63 | 8 | 8 | 0.7640 | 220.0 |
| **PD1 vs. CD47 + PD1 + tdRT** | 119.7 | 107.1 | 12.62 | 15.63 | 8 | 8 | 1.141 | 220.0 |
| **CD47 + PD1 vs. PD1 + tdRT** | 89.58 | 128.1 | -38.57 | 15.63 | 8 | 8 | 3.489 | 220.0 |
| **CD47 + PD1 vs. CD47 + PD1 + tdRT** | 89.58 | 107.1 | -17.51 | 15.63 | 8 | 8 | 1.584 | 220.0 |
| **PD1 + tdRT vs. CD47 + PD1 + tdRT** | 128.1 | 107.1 | 21.06 | 15.63 | 8 | 8 | 1.905 | 220.0 |

**Supplemental Figure 1G**

| **Days** | **Control** | **Control** | **Control** | **Control** | **Control** | **Control** | **Control** | **Control** | **anti-CD47** | **anti-CD47** | **anti-CD47** | **anti-CD47** | **anti-CD47** | **anti-CD47** | **anti-CD47** | **anti-CD47** | **tdRT** | **tdRT** | **tdRT** | **tdRT** | **tdRT** | **tdRT** | **tdRT** | **tdRT** | **anti-CD47 + tdRT** | **anti-CD47 + tdRT** | **anti-CD47 + tdRT** | **anti-CD47 + tdRT** | **anti-CD47 + tdRT** | **anti-CD47 + tdRT** | **anti-CD47 + tdRT** | **anti-CD47 + tdRT** |
| --- | --- | --- | --- | --- | --- | --- | --- | --- | --- | --- | --- | --- | --- | --- | --- | --- | --- | --- | --- | --- | --- | --- | --- | --- | --- | --- | --- | --- | --- | --- | --- | --- |
| **0** | 0.0000 | 0.000 | 0.0000 | 0.0000 | 0.000 | 0.0000 | 0.0000 | 0.0000 | 0.0000 | 0.000 | 0.0000 | 0.0000 | 0.0000 | 0.0000 | 0.0000 | 0.0000 | 0.0000 | 0.000 | 0.0000 | 0.0000 | 0.0000 | 0.0000 | 0.0000 |  | 0.0000 | 0.000 | 0.0000 | 0.0000 | 0.0000 | 0.0000 | 0.0000 |  |
| **6** | 21.7800 | 29.602 | 44.1000 | 52.9000 | 21.504 | 36.0000 | 40.0000 | 15.7500 | 51.6375 | 40.000 | 40.0000 | 33.6000 | 18.0000 | 38.4000 | 41.6000 | 54.6750 | 20.4800 | 57.600 | 50.2740 | 19.2200 | 74.3600 | 64.5120 | 27.5625 |  | 51.7720 | 29.400 | 29.6020 | 68.7500 | 13.5000 | 15.7500 | 11.7600 |  |
| **8** | 63.3600 | 118.800 | 108.0000 | 75.0000 | 75.000 | 94.1015 | 126.0000 | 63.3600 | 102.0600 | 117.000 | 67.7120 | 126.0000 | 60.7475 | 40.0000 | 58.0800 | 109.8000 | 32.8000 | 143.360 | 37.6000 | 71.5275 | 147.8750 | 107.6480 | 85.6745 |  | 58.7250 | 77.500 | 75.0000 | 102.0600 | 38.0250 | 39.2000 | 62.5000 |  |
| **10** | 68.7500 | 171.500 | 125.0235 | 107.6480 | 75.000 | 126.0000 | 117.0000 | 76.2500 | 105.8750 | 89.232 | 41.6000 | 143.3600 | 87.8800 | 40.0000 | 50.8475 | 87.5000 | 41.1845 | 126.000 | 37.2645 | 87.7250 | 189.0375 | 66.8160 | 60.7500 |  | 44.1000 | 45.600 | 102.8500 | 75.0000 | 41.1845 | 37.2645 | 50.6250 |  |
| **12** | 56.7000 |  | 97.4700 | 100.9200 | 85.176 |  | 90.7500 | 130.2350 | 75.0000 | 78.030 | 28.8800 | 191.1000 | 68.7500 | 36.1000 | 27.5625 |  | 40.0000 |  | 21.7800 | 71.6560 |  | 20.4800 | 19.2200 |  | 30.4200 | 25.600 | 62.5000 | 27.2250 | 15.7500 | 14.4000 | 11.3680 |  |
| **14** | 55.6875 |  |  | 90.7500 | 87.480 |  | 75.7120 | 154.6380 |  |  | 18.7395 | 196.0000 |  | 36.1000 | 18.7395 |  | 34.2225 |  | 25.9200 |  |  | 18.4500 | 15.2880 |  | 13.1820 | 22.050 | 19.2200 | 30.6250 | 10.5705 | 5.7330 | 4.2000 |  |
| **16** | 65.0000 |  |  | 97.5560 | 108.000 |  | 78.7320 | 196.0000 |  |  | 14.8500 | 198.4500 |  | 42.0250 | 15.8565 |  | 52.9000 |  | 22.3245 |  |  | 14.7175 | 18.0000 |  | 12.1520 | 19.350 | 32.8000 | 28.7875 | 6.6150 | 5.2920 | 4.5125 |  |
| **18** | 33.6000 |  |  | 68.7500 | 52.920 |  | 40.0000 | 149.9875 |  |  | 13.0355 | 184.9600 |  | 37.0440 | 32.0000 |  | 53.0160 |  | 14.8955 |  |  | 13.9500 | 13.0355 |  | 4.6000 | 19.220 | 24.5000 | 24.5000 | 4.6000 | 1.6875 | 0.0000 |  |

| **Within each row, compare columns (simple effects within rows)** |  |  |  |  |  |  |  |  |
| --- | --- | --- | --- | --- | --- | --- | --- | --- |
|  |  |  |  |  |  |  |  |  |
| **Number of families** | 8 |  |  |  |  |  |  |  |
| **Number of comparisons per family** | 6 |  |  |  |  |  |  |  |
| **Alpha** | 0.05 |  |  |  |  |  |  |  |
|  |  |  |  |  |  |  |  |  |
| **Tukey's multiple comparisons test** | Predicted (LS) mean diff. | 95.00% CI of diff. | Below threshold? | Summary | Adjusted P Value |  |  |  |
|  |  |  |  |  |  |  |  |  |
| **Row 1** |  |  |  |  |  |  |  |  |
| **Control vs. anti-CD47** | 0.000 | -43.36 to 43.36 | No | ns | >0.9999 |  |  |  |
| **Control vs. tdRT** | 0.000 | -44.88 to 44.88 | No | ns | >0.9999 |  |  |  |
| **Control vs. anti-CD47 + tdRT** | 0.000 | -44.88 to 44.88 | No | ns | >0.9999 |  |  |  |
| **anti-CD47 vs. tdRT** | 0.000 | -44.88 to 44.88 | No | ns | >0.9999 |  |  |  |
| **anti-CD47 vs. anti-CD47 + tdRT** | 0.000 | -44.88 to 44.88 | No | ns | >0.9999 |  |  |  |
| **tdRT vs. anti-CD47 + tdRT** | 0.000 | -46.35 to 46.35 | No | ns | >0.9999 |  |  |  |
|  |  |  |  |  |  |  |  |  |
| **Row 2** |  |  |  |  |  |  |  |  |
| **Control vs. anti-CD47** | -7.035 | -50.39 to 36.32 | No | ns | 0.9748 |  |  |  |
| **Control vs. tdRT** | -12.15 | -57.03 to 32.72 | No | ns | 0.8960 |  |  |  |
| **Control vs. anti-CD47 + tdRT** | 1.200 | -43.68 to 46.08 | No | ns | 0.9999 |  |  |  |
| **anti-CD47 vs. tdRT** | -5.119 | -50.00 to 39.76 | No | ns | 0.9910 |  |  |  |
| **anti-CD47 vs. anti-CD47 + tdRT** | 8.234 | -36.64 to 53.11 | No | ns | 0.9643 |  |  |  |
| **tdRT vs. anti-CD47 + tdRT** | 13.35 | -33.00 to 59.70 | No | ns | 0.8777 |  |  |  |
|  |  |  |  |  |  |  |  |  |
| **Row 3** |  |  |  |  |  |  |  |  |
| **Control vs. anti-CD47** | 5.278 | -38.08 to 48.63 | No | ns | 0.9891 |  |  |  |
| **Control vs. tdRT** | 0.9548 | -43.92 to 45.83 | No | ns | >0.9999 |  |  |  |
| **Control vs. anti-CD47 + tdRT** | 25.74 | -19.14 to 70.62 | No | ns | 0.4471 |  |  |  |
| **anti-CD47 vs. tdRT** | -4.323 | -49.20 to 40.56 | No | ns | 0.9945 |  |  |  |
| **anti-CD47 vs. anti-CD47 + tdRT** | 20.46 | -24.42 to 65.34 | No | ns | 0.6386 |  |  |  |
| **tdRT vs. anti-CD47 + tdRT** | 24.78 | -21.57 to 71.13 | No | ns | 0.5092 |  |  |  |
|  |  |  |  |  |  |  |  |  |
| **Row 4** |  |  |  |  |  |  |  |  |
| **Control vs. anti-CD47** | 27.61 | -15.75 to 70.97 | No | ns | 0.3525 |  |  |  |
| **Control vs. tdRT** | 21.43 | -23.45 to 66.31 | No | ns | 0.6032 |  |  |  |
| **Control vs. anti-CD47 + tdRT** | 51.74 | 6.857 to 96.61 | Yes | * | 0.0167 |  |  |  |
| **anti-CD47 vs. tdRT** | -6.181 | -51.06 to 38.70 | No | ns | 0.9843 |  |  |  |
| **anti-CD47 vs. anti-CD47 + tdRT** | 24.13 | -20.75 to 69.00 | No | ns | 0.5044 |  |  |  |
| **tdRT vs. anti-CD47 + tdRT** | 30.31 | -16.04 to 76.66 | No | ns | 0.3288 |  |  |  |
|  |  |  |  |  |  |  |  |  |
| **Row 5** |  |  |  |  |  |  |  |  |
| **Control vs. anti-CD47** | 21.34 | -26.90 to 69.58 | No | ns | 0.6607 |  |  |  |
| **Control vs. tdRT** | 58.91 | 6.407 to 111.4 | Yes | * | 0.0211 |  |  |  |
| **Control vs. anti-CD47 + tdRT** | 66.79 | 18.55 to 115.0 | Yes | ** | 0.0024 |  |  |  |
| **anti-CD47 vs. tdRT** | 37.58 | -13.20 to 88.35 | No | ns | 0.2235 |  |  |  |
| **anti-CD47 vs. anti-CD47 + tdRT** | 45.45 | -0.8992 to 91.80 | No | ns | 0.0568 |  |  |  |
| **tdRT vs. anti-CD47 + tdRT** | 7.875 | -42.90 to 58.65 | No | ns | 0.9779 |  |  |  |
|  |  |  |  |  |  |  |  |  |
| **Row 6** |  |  |  |  |  |  |  |  |
| **Control vs. anti-CD47** | 25.46 | -32.71 to 83.63 | No | ns | 0.6682 |  |  |  |
| **Control vs. tdRT** | 69.38 | 11.21 to 127.6 | Yes | * | 0.0122 |  |  |  |
| **Control vs. anti-CD47 + tdRT** | 77.77 | 27.00 to 128.5 | Yes | *** | 0.0006 |  |  |  |
| **anti-CD47 vs. tdRT** | 43.92 | -17.39 to 105.2 | No | ns | 0.2500 |  |  |  |
| **anti-CD47 vs. anti-CD47 + tdRT** | 52.31 | -2.039 to 106.7 | No | ns | 0.0639 |  |  |  |
| **tdRT vs. anti-CD47 + tdRT** | 8.387 | -45.96 to 62.74 | No | ns | 0.9782 |  |  |  |
|  |  |  |  |  |  |  |  |  |
| **Row 7** |  |  |  |  |  |  |  |  |
| **Control vs. anti-CD47** | 41.26 | -16.91 to 99.43 | No | ns | 0.2582 |  |  |  |
| **Control vs. tdRT** | 82.07 | 23.90 to 140.2 | Yes | ** | 0.0019 |  |  |  |
| **Control vs. anti-CD47 + tdRT** | 93.41 | 42.64 to 144.2 | Yes | **** | <0.0001 |  |  |  |
| **anti-CD47 vs. tdRT** | 40.81 | -20.51 to 102.1 | No | ns | 0.3131 |  |  |  |
| **anti-CD47 vs. anti-CD47 + tdRT** | 52.15 | -2.200 to 106.5 | No | ns | 0.0651 |  |  |  |
| **tdRT vs. anti-CD47 + tdRT** | 11.34 | -43.01 to 65.69 | No | ns | 0.9488 |  |  |  |
|  |  |  |  |  |  |  |  |  |
| **Row 8** |  |  |  |  |  |  |  |  |
| **Control vs. anti-CD47** | 2.292 | -55.88 to 60.46 | No | ns | 0.9996 |  |  |  |
| **Control vs. tdRT** | 45.33 | -12.84 to 103.5 | No | ns | 0.1841 |  |  |  |
| **Control vs. anti-CD47 + tdRT** | 57.75 | 6.976 to 108.5 | Yes | * | 0.0188 |  |  |  |
| **anti-CD47 vs. tdRT** | 43.04 | -18.28 to 104.4 | No | ns | 0.2671 |  |  |  |
| **anti-CD47 vs. anti-CD47 + tdRT** | 55.46 | 1.108 to 109.8 | Yes | * | 0.0436 |  |  |  |
| **tdRT vs. anti-CD47 + tdRT** | 12.42 | -41.93 to 66.77 | No | ns | 0.9341 |  |  |  |
|  |  |  |  |  |  |  |  |  |
|  |  |  |  |  |  |  |  |  |
| **Test details** | Predicted (LS) mean 1 | Predicted (LS) mean 2 | Predicted (LS) mean diff. | SE of diff. | N1 | N2 | q | DF |
|  |  |  |  |  |  |  |  |  |
| **Row 1** |  |  |  |  |  |  |  |  |
| **Control vs. anti-CD47** | 0.000 | 0.000 | 0.000 | 16.71 | 8 | 8 | 0.000 | 173.0 |
| **Control vs. tdRT** | 0.000 | 0.000 | 0.000 | 17.30 | 8 | 7 | 0.000 | 173.0 |
| **Control vs. anti-CD47 + tdRT** | 0.000 | 0.000 | 0.000 | 17.30 | 8 | 7 | 0.000 | 173.0 |
| **anti-CD47 vs. tdRT** | 0.000 | 0.000 | 0.000 | 17.30 | 8 | 7 | 0.000 | 173.0 |
| **anti-CD47 vs. anti-CD47 + tdRT** | 0.000 | 0.000 | 0.000 | 17.30 | 8 | 7 | 0.000 | 173.0 |
| **tdRT vs. anti-CD47 + tdRT** | 0.000 | 0.000 | 0.000 | 17.87 | 7 | 7 | 0.000 | 173.0 |
|  |  |  |  |  |  |  |  |  |
| **Row 2** |  |  |  |  |  |  |  |  |
| **Control vs. anti-CD47** | 32.70 | 39.74 | -7.035 | 16.71 | 8 | 8 | 0.5952 | 173.0 |
| **Control vs. tdRT** | 32.70 | 44.86 | -12.15 | 17.30 | 8 | 7 | 0.9935 | 173.0 |
| **Control vs. anti-CD47 + tdRT** | 32.70 | 31.50 | 1.200 | 17.30 | 8 | 7 | 0.09807 | 173.0 |
| **anti-CD47 vs. tdRT** | 39.74 | 44.86 | -5.119 | 17.30 | 8 | 7 | 0.4185 | 173.0 |
| **anti-CD47 vs. anti-CD47 + tdRT** | 39.74 | 31.50 | 8.234 | 17.30 | 8 | 7 | 0.6731 | 173.0 |
| **tdRT vs. anti-CD47 + tdRT** | 44.86 | 31.50 | 13.35 | 17.87 | 7 | 7 | 1.057 | 173.0 |
|  |  |  |  |  |  |  |  |  |
| **Row 3** |  |  |  |  |  |  |  |  |
| **Control vs. anti-CD47** | 90.45 | 85.17 | 5.278 | 16.71 | 8 | 8 | 0.4466 | 173.0 |
| **Control vs. tdRT** | 90.45 | 89.50 | 0.9548 | 17.30 | 8 | 7 | 0.07805 | 173.0 |
| **Control vs. anti-CD47 + tdRT** | 90.45 | 64.72 | 25.74 | 17.30 | 8 | 7 | 2.104 | 173.0 |
| **anti-CD47 vs. tdRT** | 85.17 | 89.50 | -4.323 | 17.30 | 8 | 7 | 0.3534 | 173.0 |
| **anti-CD47 vs. anti-CD47 + tdRT** | 85.17 | 64.72 | 20.46 | 17.30 | 8 | 7 | 1.672 | 173.0 |
| **tdRT vs. anti-CD47 + tdRT** | 89.50 | 64.72 | 24.78 | 17.87 | 7 | 7 | 1.962 | 173.0 |
|  |  |  |  |  |  |  |  |  |
| **Row 4** |  |  |  |  |  |  |  |  |
| **Control vs. anti-CD47** | 108.4 | 80.79 | 27.61 | 16.71 | 8 | 8 | 2.336 | 173.0 |
| **Control vs. tdRT** | 108.4 | 86.97 | 21.43 | 17.30 | 8 | 7 | 1.752 | 173.0 |
| **Control vs. anti-CD47 + tdRT** | 108.4 | 56.66 | 51.74 | 17.30 | 8 | 7 | 4.229 | 173.0 |
| **anti-CD47 vs. tdRT** | 80.79 | 86.97 | -6.181 | 17.30 | 8 | 7 | 0.5053 | 173.0 |
| **anti-CD47 vs. anti-CD47 + tdRT** | 80.79 | 56.66 | 24.13 | 17.30 | 8 | 7 | 1.972 | 173.0 |
| **tdRT vs. anti-CD47 + tdRT** | 86.97 | 56.66 | 30.31 | 17.87 | 7 | 7 | 2.399 | 173.0 |
|  |  |  |  |  |  |  |  |  |
| **Row 5** |  |  |  |  |  |  |  |  |
| **Control vs. anti-CD47** | 93.54 | 72.20 | 21.34 | 18.60 | 6 | 7 | 1.623 | 173.0 |
| **Control vs. tdRT** | 93.54 | 34.63 | 58.91 | 20.24 | 6 | 5 | 4.116 | 173.0 |
| **Control vs. anti-CD47 + tdRT** | 93.54 | 26.75 | 66.79 | 18.60 | 6 | 7 | 5.079 | 173.0 |
| **anti-CD47 vs. tdRT** | 72.20 | 34.63 | 37.58 | 19.57 | 7 | 5 | 2.715 | 173.0 |
| **anti-CD47 vs. anti-CD47 + tdRT** | 72.20 | 26.75 | 45.45 | 17.87 | 7 | 7 | 3.598 | 173.0 |
| **tdRT vs. anti-CD47 + tdRT** | 34.63 | 26.75 | 7.875 | 19.57 | 5 | 7 | 0.5690 | 173.0 |
|  |  |  |  |  |  |  |  |  |
| **Row 6** |  |  |  |  |  |  |  |  |
| **Control vs. anti-CD47** | 92.85 | 67.39 | 25.46 | 22.42 | 5 | 4 | 1.606 | 173.0 |
| **Control vs. tdRT** | 92.85 | 23.47 | 69.38 | 22.42 | 5 | 4 | 4.376 | 173.0 |
| **Control vs. anti-CD47 + tdRT** | 92.85 | 15.08 | 77.77 | 19.57 | 5 | 7 | 5.619 | 173.0 |
| **anti-CD47 vs. tdRT** | 67.39 | 23.47 | 43.92 | 23.64 | 4 | 4 | 2.628 | 173.0 |
| **anti-CD47 vs. anti-CD47 + tdRT** | 67.39 | 15.08 | 52.31 | 20.95 | 4 | 7 | 3.531 | 173.0 |
| **tdRT vs. anti-CD47 + tdRT** | 23.47 | 15.08 | 8.387 | 20.95 | 4 | 7 | 0.5661 | 173.0 |
|  |  |  |  |  |  |  |  |  |
| **Row 7** |  |  |  |  |  |  |  |  |
| **Control vs. anti-CD47** | 109.1 | 67.80 | 41.26 | 22.42 | 5 | 4 | 2.602 | 173.0 |
| **Control vs. tdRT** | 109.1 | 26.99 | 82.07 | 22.42 | 5 | 4 | 5.176 | 173.0 |
| **Control vs. anti-CD47 + tdRT** | 109.1 | 15.64 | 93.41 | 19.57 | 5 | 7 | 6.750 | 173.0 |
| **anti-CD47 vs. tdRT** | 67.80 | 26.99 | 40.81 | 23.64 | 4 | 4 | 2.442 | 173.0 |
| **anti-CD47 vs. anti-CD47 + tdRT** | 67.80 | 15.64 | 52.15 | 20.95 | 4 | 7 | 3.520 | 173.0 |
| **tdRT vs. anti-CD47 + tdRT** | 26.99 | 15.64 | 11.34 | 20.95 | 4 | 7 | 0.7656 | 173.0 |
|  |  |  |  |  |  |  |  |  |
| **Row 8** |  |  |  |  |  |  |  |  |
| **Control vs. anti-CD47** | 69.05 | 66.76 | 2.292 | 22.42 | 5 | 4 | 0.1445 | 173.0 |
| **Control vs. tdRT** | 69.05 | 23.72 | 45.33 | 22.42 | 5 | 4 | 2.859 | 173.0 |
| **Control vs. anti-CD47 + tdRT** | 69.05 | 11.30 | 57.75 | 19.57 | 5 | 7 | 4.173 | 173.0 |
| **anti-CD47 vs. tdRT** | 66.76 | 23.72 | 43.04 | 23.64 | 4 | 4 | 2.575 | 173.0 |
| **anti-CD47 vs. anti-CD47 + tdRT** | 66.76 | 11.30 | 55.46 | 20.95 | 4 | 7 | 3.744 | 173.0 |
| **tdRT vs. anti-CD47 + tdRT** | 23.72 | 11.30 | 12.42 | 20.95 | 4 | 7 | 0.8386 | 173.0 |
