# Supplementary material for: CD47 blockade (ALX301) enhances immunoradiotherapy response in HPV negative head and neck squamous cell carcinoma
Source: PLoS One. 2026 Feb 17;21(2):e0328031. doi: 10.1371/journal.pone.0328031 (PMC12912607; doi:10.1371/journal.pone.0328031)
Supplement: Attachment — Submitted filename: Response to Reviewers.docx [file pone.0328031.s005.docx]

Response to the Reviewers

**Reviewer #2:** Comment: Provide exact sample sizes (number of mice per group) for all experiments in the Methods section. Additionally, specify the statistical tests used for each figure panel (e.g., ANOVA/t-test) in figure legends or methods.

We thank the reviewer for this suggestion. The exact sample sizes for all experiments have been added to the corresponding figure legends, as some experiments had varying group sizes. Additionally, the specific statistical tests used for each experiment have been indicated in the figure legends.

**Reviewer #3:** General Comments:

This is an elegant and scientifically rigorous manuscript that explores the potential of CD47 immune checkpoint blockade in improving immunoradiotherapy responses in HPV-negative head and neck squamous cell carcinoma (HNSCC). The study is well designed, the models are appropriate, and the results are clearly presented.

Strengths:

Appropriate use of syngeneic murine models including both PD-1-sensitive and resistant tumors (4MOSC1 and 4MOSC2).

Integration of anti-CD47 therapy with PD-1 inhibition and tumor-directed radiation is timely and translationally relevant.

Immunophenotyping and TCR sequencing add strong mechanistic support.

Figures are informative and the manuscript is clearly written.

Optional Suggestions to Improve the Final Version:

The authors may consider adding a brief discussion of ongoing clinical trials involving CD47 inhibitors in HNSCC to enhance the translational context.

We thank the reviewer for this suggestion. We discuss current ongoing clinical trials and results that have been published. Page 17, Line 404: “It was found to have a favorable safety profile in clinical studies [20]. Additionally, in preclinical models, evorpacept combined with anti-PD1 led to favorable outcomes such as increased tumor growth inhibition and extended survivability [21]. While phase 2 clinical trials investigating evorpacept, pembrolizumab, and chemotherapy combination therapy for locally advanced HNSCC failed to meet primary endpoints, these findings suggest that additional or alternative therapeutic partners may be necessary for the synergistic potential of CD47 and PD1 inhibition (NCT05787639 and NCT04675333) [33].”

Moving the limitations paragraph earlier in the Discussion section might improve the overall flow of the manuscript.
We thank the reviewer for this suggestion. The discussion, including the limitations, has been edited to improve overall flow of the manuscript.

Overall, the manuscript is solid and presents impactful findings. I support publication without the need for revision.

**Reviewer #4:** Review Comments to the Author
Manuscript Title: CD47 Blockade Enhances Immunradiotherapy Response in Head and Neck Squamous Cell Carcinoma

Manuscript Number: PONE-D-25-34525

Dear Authors,

Your study on combining CD47 blockade with anti-PD1 therapy and radiotherapy in HPV-negative head and neck squamous cell carcinoma (HNSCC) is compelling and offers fresh insights into immunotherapy. Below, I’ve summarized my thoughts on the manuscript’s technical quality, data support, statistical rigor, data availability, and presentation, along with a few suggestions. I’ve kept this concise and focused, aiming for clarity and a natural tone.

1. Is the Manuscript Technically Sound?
Assessment: The study is solid, with a thoughtful design and reliable methods, though some missing details and text gaps need fixing.

You’ve done a great job laying out the goal—to test CD47 blockade (ALX301) with anti-PD1 and radiotherapy in 4MOSC1 and 4MOSC2 mouse models . These models mimic HNSCC well, and testing both responsive and resistant cell lines adds depth.

The CRISPR/Cas9 knockout , flow cytometry , and TCR sequencing are described clearly, with standard protocols that make replication possible. Verifications like Western blots are a nice touch.

The study follows UCSD IACUC guidelines, with clear animal welfare measures.


Text Gaps: Some sections cut off abruptly , making it hard to follow the full story.

We thank the reviewer for this helpful observation. We have added section titles and revised portions of the text to improve flow and ensure continuity throughout the manuscript.

2. Do the Data Support the Conclusions?
Assessment: Your data back the claims well, showing CD47 blockade boosts anti-tumor effects, especially with anti-PD1 and radiotherapy, though a few gaps need addressing.

The TCGA analysis linking high CD47 to worse survival sets a strong foundation.

Complete tumor regression in 4MOSC1 with CD47 knockout or combination therapy and better outcomes in 4MOSC2 with the triple regimen are convincing.

Increased MHC-II/CD86 expression and TCR clonality support enhanced immune activation.
You tie results to clinical needs, noting prior trial challenges.

Some immune markers (e.g., CD8 T-cell counts) weren’t significant (Page 17, Line 208), and TCR sequencing used only one sample per group (Page 29, Line 488).

We thank the reviewer for this observation. We have added statistical significance indicators to clarify which findings were and were not significant, improving transparency. Additionally, we have clarified in the text that the TCR sequencing results are based on limited sampling, providing supportive context to the flow cytometry findings.

Page 18, Line 436: “It is important to note that these observations, while suggestive of our hypothesis that ALX301 increases trafficking and migration of APCs, are based on limited sampling.”

Page 19, Line 457: “Another limitation is the T-cell receptor sequencing analysis, which was performed on a single sample per group. These results provide a snapshot of clonality to provide insight into the flow cytometry results of the tumors and lymph nodes, but not statistical results. Future studies with larger sample sizes are warranted to confirm the observed clonal expansion patterns with statistical comparisons.”


Report p-values for all key findings, including non-significant ones.

We thank the reviewer for this suggestion. All p-values, including non-significant ones, have been written out in each of the figure legends.

Justify or expand the TCR sequencing sample size.

We thank the reviewer for this suggestion. We have expanded and justified the low sampling size and acknowledged it as a limitation of this study.

Page 18, Line 436: “It is important to note that these observations, while suggestive of our hypothesis that ALX301 increases trafficking and migration of APCs, are based on limited sampling.”

Page 19, Line 457: “Another limitation is the T-cell receptor sequencing analysis, which was performed on a single sample per group. These results provide a snapshot of clonality to provide insight into the flow cytometry results of the tumors and lymph nodes, but not statistical results. Future studies with larger sample sizes are warranted to confirm the observed clonal expansion patterns with statistical comparisons.”

Clarify how results might translate to human patients.
We thank the reviewer for this suggestion. We have added to the discussion how our results might translate to human patients.

Page 20, Line 479: “Our results indicate that CD47 blockade, alone or in combination with anti-PD1 and radiotherapy, enhances dendritic cell activation and T-cell expansion in murine HPV-negative HNSCC models. The increased clonality and overlap between tumors and draining lymph nodes suggest that CD47 inhibition may influence antigen-specific immune trafficking between the tumor and lymph node, providing rational to lymphatic preservation. While these effects were observed in mice, they point to mechanisms that could inform the design of future clinical studies evaluating CD47-targeted combinations and treatment sequencing in HNSCC.”

3. Has the Statistical Analysis Been Performed Appropriately and Rigorously?
Assessment: The stats are solid for the most part, using the right tests, but clearer reporting on sample sizes and corrections would help.

T-tests, ANOVA, and log-rank tests fit well for tumor growth, immune data, and survival. Significant p-values support your claims.

You’re upfront about non-significant results, which builds trust.

GraphPad Prism 10 is a reliable choice.

Name the post-hoc test for ANOVA (e.g., Tukey’s).
Confirm data meet test assumptions or consider non-parametric options.

We thank the reviewer for this suggestion. The specific statistical tests used for each experiment have been indicated in the figure legends.

4. Have the Authors Made All Data Underlying the Findings Fully Available?
Assessment: You state all data are in the manuscript or Supporting Information (Page 7, Lines 7–8), which meets PLOS ONE’s rules, but I can’t fully verify without the files.

You say all data are available without restriction, aligning with journal policy.
In-Text Data: Tumor growth, survival, and immune results are summarized well , and TCGA data are public

Consider depositing TCR sequencing data in a repository like SRA.

We thank the reviewer for this suggestion. We have deposited the TCR sequencing data into SRA with BioProject accession number [PRJNA1372401](https://dataview.ncbi.nlm.nih.gov/object/PRJNA1372401).

5. Is the Manuscript Presented in an Intelligible Fashion and Written in Standard English?
The manuscript is clear and well-written, with a professional tone, but typos and missing text need fixing.

We thank the reviewer for this observation. We have gone through the manuscript and have rewritten/reviewed the typos and missing text.

**Reviewer #5:** This manuscript presents important findings on the immunological enhancement provided by CD47 blockade in combination with anti-PD1 and radiotherapy in HNSCC models. It employs robust murine models (4MOSC1 and 4MOSC2), sophisticated CRISPR-based knockout systems, and insightful immune profiling. The translational implications are clear, especially for HPV-negative HNSCC, a subgroup with limited response to checkpoint inhibitors. However, the study would benefit significantly from additional validation in key areas, especially concerning TCR sequencing robustness, toxicity profiling, and therapeutic sequencing.

We thank the reviewer for their suggestion.

Regarding the TCR sequencing robustness, we have expanded and justified the low sampling size and acknowledged it as a limitation of this study. Page 18, Line 436: “It is important to note that these observations, while suggestive of our hypothesis that ALX301 increases trafficking and migration of APCs, are based on limited sampling.” Page 19, Line 457: “Another limitation is the T-cell receptor sequencing analysis, which was performed on a single sample per group. These results provide a snapshot of clonality to provide insight into the flow cytometry results of the tumors and lymph nodes, but not statistical results. Future studies with larger sample sizes are warranted to confirm the observed clonal expansion patterns with statistical comparisons.”

For toxicity profiling of anti-CD47, we acknowledged past literature that have explored the safety profile of CD47 inhibition and discussed toxicity profiling as next steps for future studies. Page 19, Line 447: “Past studies have demonstrated that CD47 blockade expresses minimal drug toxicity due to the lack of pro-phagocytic signals on healthy cells [34]. Throughout our study, no toxicity was observed in mice who received ALX301 therapy. It is important to note that while mice and humans share many aspects of cellular identity, key differences exist that impact the immune response to cancer and CD47 blockade [35]. Such differences include expression and binding of selectins, cytokine production, and Fc receptor expression [35]. Therefore, future studies that investigate the long-term toxicity profile of anti-CD47 are warranted to ensure safety during prolonged treatment in a clinical setting.”

For therapeutic sequencing, we have acknowledged prior studies that provide evidence on dosing and sequencing strategies that enhance therapeutic synergy. Page 9, Line 201: “A one-time dose of 4Gy was chosen for tdRT based on results that provided evidence that this dose of tdRT was not cytotoxic but still enhanced the percentage of CD8+ T cells and dendritic cell trafficking to the 4MOSC tumor [26]. With SBRT, there was an enrichment of tumor-specific T cells and CD8+ cytotoxic T cells, indicating an enhancement of an antitumor response [15]. Furthermore, we chose to deliver the tdRT before ICI administration due to evidence that delivering irradiation treatment directly to the gross tumor and sparing the cervical lymphatics can improve the systemic response that anti-PD1 and other immunotherapies provide [12,16].”

The manuscript shows increased MHC-II and CD86 expression but does not confirm whether DCs undergo full activation or cross-priming. Cytokine profiling or transcriptomic analysis of sorted DCs would strengthen the immunological conclusions.

We thank the reviewer for this insight. We have acknowledged in the text that while MHC-II and CD86 expression is a phenotype associated with DC activation, our studies do not confirm cross priming of tumor directed cytotoxic T cells by DCs.

Page 12, Line 286: “While increased MHC II and CD86 expression is consistent with a more activated dendritic cell phenotype, our study does not directly demonstrate cross-priming of tumor-specific cytotoxic T cells by DCs.”

Given the translational intent of the ALX301 combination regimen, the lack of toxicity profiling (e.g., hematological, hepatic, or general behavior outcomes) is a limitation. Basic safety data would bolster clinical relevance.

We thank the reviewer for this suggestion. We acknowledged past literature that have explored the safety profile of CD47 inhibition and discussed toxicity profiling as next steps for future studies. Page 19, Line 447: “Past studies have demonstrated that CD47 blockade expresses minimal drug toxicity due to the lack of pro-phagocytic signals on healthy cells [34]. Throughout our study, no toxicity was observed in mice who received ALX301 therapy. It is important to note that while mice and humans share many aspects of cellular identity, key differences exist that impact the immune response to cancer and CD47 blockade [35]. Such differences include expression and binding of selectins, cytokine production, and Fc receptor expression [35]. Therefore, future studies that investigate the long-term toxicity profile of anti-CD47 are warranted to ensure safety during prolonged treatment in a clinical setting.”

Only one tumor and SLN per group were sequenced, making the clonality findings anecdotal. Including replicates and statistical tests (e.g., diversity indices, clonotype frequency analysis) would solidify these claims.

We thank the reviewer for this suggestion. We have expanded and justified the low sampling size and acknowledged it as a limitation of this study. Page 18, Line 436: “It is important to note that these observations, while suggestive of our hypothesis that ALX301 increases trafficking and migration of APCs, are based on limited sampling.” Page 19, Line 457: “Another limitation is the T-cell receptor sequencing analysis, which was performed on a single sample per group. These results provide a snapshot of clonality to provide insight into the flow cytometry results of the tumors and lymph nodes, but not statistical results. Future studies with larger sample sizes are warranted to confirm the observed clonal expansion patterns with statistical comparisons.”

The manuscript lacks a clear rationale for using 4 Gy and applying it before ICIs. Comparative timing (pre vs post-ICI) would test hypotheses around immune priming and synergy.

We thank the reviewer for this suggestion. We have acknowledged prior studies that provide evidence on dosing and sequencing strategies that enhance therapeutic synergy. Page 9, Line 201: “A one-time dose of 4Gy was chosen for tdRT based on results that provided evidence that this dose of tdRT was not cytotoxic but still enhanced the percentage of CD8+ T cells and dendritic cell trafficking to the 4MOSC tumor [26]. With SBRT, there was an enrichment of tumor-specific T cells and CD8+ cytotoxic T cells, indicating an enhancement of an antitumor response [15]. Furthermore, we chose to deliver the tdRT before ICI administration due to evidence that delivering irradiation treatment directly to the gross tumor and sparing the cervical lymphatics can improve the systemic response that anti-PD1 and other immunotherapies provide [12,16].”

Consider specifying “HPV-negative” in the title for clarity.

We thank the reviewer for this suggestion. We have specified “HPV-negative” in the title of the manuscript.

**Reviewer #6:** Abstract
• “Using the 4MOSC1 orthotopic, syngeneic murine model of HPV-negative HNSCC, treatment with an engineered CD47-blocking SIRPα fusion protein (ALX301) similarly induced complete tumor regression in combination with anti-PD1, and a partial response as a standalonetherapeutic.” – This sentence is hard to follow and “similarly” lacks a clear reference. Consider rewriting for clarity and brevity. For example: “In the 4MOSC1 syngeneic HPV-negative HNSCC mouse model, ALX301 (an engineered CD47-blocking SIRPα fusion) induced complete tumor regression only when combined with anti–PD-1, whereas it produced only a partial tumor response as a monotherapy.” This explicitly states the outcome and makes it clear that combination therapy was required for complete regression.

We thank the reviewer for this suggestion. We have revised this section for clarity as recommended. Page 2, Line 38: “In the 4MOSC1 syngeneic HPV-negative HNSCC mouse model, ALX301 (an engineered CD47-blocking SIRPα fusion for murine models) induced complete tumor regression when combined with anti–PD-1, and produced a partial tumor response as a monotherapy.”

• Quantify key outcomes: The abstract would be stronger if it quantified or defined terms like “partial response” and “complete tumor regression.” For instance, specify the percentage of mice with complete tumor eradication vs. tumor reduction. If “partial response” means tumor shrinkage without full disappearance, clarify this. Adding a brief quantitative result (e.g., “complete regression in 5/5 mice vs. partial regression in 0/5 with monotherapy”) will help readers grasp the magnitude of the effect.
Introduction

We thank the reviewer for this suggestion. We defined both completed tumor response and partial response in the manuscript.

Page 6, Line 135: “…resulting in complete tumor regression, defined as complete or almost complete tumor eradication…”

Page 9, Line 196: “…with partial tumor regression defined as tumor reduction but not complete tumor eradication…”

We have also included in the figure legends, how many mice had complete tumor regression vs partial tumor regression in each group. For example, page 7 line 166: “In the 4MOSC1 Parent group, 2/10 mice had a partial reduction in tumor size, while 8/10 had continuous tumor growth. In the 4MOSC1 Parent + anti-PD1 group, 3/10 mice had complete tumor eradication, 6/10 mice had tumor reduction, and 1/10 mice had continuous tumor growth. In the 4MOSC1 CD47KO group, 10/10 mice had complete tumor eradication. In the 4MOSC1 CD47KO + anti-PD1 group, 9/10 mice had complete tumor eradication, and 1/10 mice had tumor reduction.”

• “Recently, PD-1 inhibitors demonstrated improved event free survival in a curative setting in locoregionally advanced disease when given as neoadjuvant prior to surgery and with post-operative adjuvant therapy in addition to adjuvant with chemotherapy and radiation [11]. However, a clear improvement in overall survival has not been demonstrated.” – This sentence is long and convoluted, making it difficult to parse the treatment timeline. Consider breaking it up and clarifying the sequence. For example: “Recent trials have shown that PD-1 inhibitors can improve event-free survival in locoregionally advanced HNSCC when used neoadjuvantly (before surgery) and continued post-operatively alongside chemotherapy and radiation [11]. However, this approach did not yield a clear overall survival benefit.” This revision simplifies the structure and makes the outcome clear.

We thank the reviewer for this suggestion. We have revised this section for clarity as recommended. Page 3, Line 68: “Recent trials have shown that PD-1 inhibitors can improve event-free survival in locoregionally advanced HNSCC when used neoadjuvantly (before surgery) and continued post-operatively alongside chemotherapy and radiation”

• “However, and critically, although radiation increases tumor antigenicity and PD-1 blockade inhibits T cell exhaustion, tumors can modulate the surrounding microenvironment to escape therapy by upregulating other immunosuppressive markers [18].” – The phrase “however, and critically,” is stylistically awkward. Instead, directly state the critical issue: “However, a critical issue remains: although radiation increases tumor antigenicity and PD-1 blockade reverses T-cell exhaustion, tumors often escape via upregulation of other immunosuppressive checkpoints [18].” This reads more clearly and sets up the rationale for investigating CD47.

We thank the reviewer for this suggestion. We have revised this section for clarity as recommended. Page 4, Line 88: “However, a critical issue remains: although radiation increases tumor antigenicity and PD-1 blockade reverses T cell exhaustion, tumors can modulate the surrounding microenvironment to escape therapy via upregulation of other immunosuppressive markers [18]”

• “We hypothesized that CD47 blockade would enhance PD-1 inhibitor and radiation tumor response in HNSCC by enhancing phagocytic tumor-specific antigen (TSA) presentation, resulting in improved immune-mediated tumor cell death via (enhanced cytotoxic T cell-mediated cell death) expansion of the TSA T cell repertoire and the immunopeptidome.” – This hypothesis statement is difficult to follow due to the parenthetical and dense phrasing. Simplify and clarify the intended mechanism. For example: “We hypothesized that CD47 blockade would improve the tumor response to PD-1 inhibition and radiotherapy by promoting phagocytosis of tumor antigens and enhancing their presentation to T cells. This, in turn, would expand the tumor-specific T-cell repertoire and strengthen cytotoxic T-cell–mediated tumor cell killing.” This version removes the confusing parentheses and clearly links CD47 blockade to enhanced antigen presentation and T-cell response.

We thank the reviewer for this suggestion. We have revised this section for clarity as recommended. Page 5, Line 108: “We hypothesized that ALX301 would improve the tumor response to PD-1 inhibition and radiotherapy by promoting phagocytosis of tumor antigens and enhancing their presentation to T cells. This, in turn, would expand the tumor-specific T-cell repertoire and strengthen cytotoxic T-cell–mediated tumor cell killing.”

• Focus and length: The Introduction is thorough, but it could be streamlined to highlight the knowledge gap earlier. You provide extensive background on HNSCC treatments, SBRT, and CD47 biology, which is useful, but readers should quickly grasp why this study is needed. Consider shortening or combining some background details. For instance, the clinical SBRT results (lines 793–802) could be summarized more succinctly. Ensure the introduction transitions clearly from what is known (e.g., PD-1 therapy limits and SBRT benefits) to the specific gap: the potential of CD47 blockade in lymphatic-sparing immunoradiotherapy. In particular, the sentence “However, many of these studies do not investigate the lymphatic sparing immunoradiotherapy benefit when combined with anti-CD47 activity.” effectively identifies the gap – you might even move this idea up sooner, so the rationale for your study is evident. Finally, at the end of the Introduction, explicitly state the study’s objectives. For example: “In this study, we evaluate the effects of CD47 blockade (using the engineered SIRPα-Fc fusion ALX301) in combination with anti–PD-1 therapy and tumor-directed radiotherapy in preclinical HNSCC models. We aim to determine whether CD47 inhibition can enhance antigen presentation, T-cell activation, and ultimately improve tumor control, thereby providing a rationale for new immunoradiotherapy combinations in HNSCC.” This gives the reader a clear roadmap of what was done.

We thank the reviewer for their suggestion. We have shortened the introduction by deleting information such as the impact of SBRT in HPV Positive HNSCC since our study focuses on HPV Negative HNSCC and rewording parts of the introduction to be more concise.

We have also added our aims to be more direct and transparent. Page 5, Line 103: “In this study, we evaluate the effects of a mouse-specific engineered CD47-blocking SIRPα fusion protein (ALX301) in combination with anti-PD1 monoclonal antibody and tumor-directed radiation therapy in preclinical HPV negative HNSCC models. We aim to determine whether CD47 inhibition can enhance antigen presentation, T-cell activation, and ultimately improve tumor control, thereby providing rationale for new immunoradiotherapy combinations in HSCC.”

Methods
• Animal experiment details: Ensure that all necessary details for reproducibility are provided. For example, clarify the number of animals used per experimental group and any animal characteristics (strain, sex, age) if not already stated. It’s mentioned that “mice were treated with… tdRT on day 6 post transplant… anti-PD1 on days 6 and 8… anti-CD47 on days 6, 10, 14, 18” – but how many mice were in each treatment group? If not in the main text, report it either here or in figure legends (e.g., “n = 10 mice per group” as appears in Fig. 2 legend). Also, please state whether mice were randomly assigned to treatment groups and whether investigators were blinded during outcome assessment, in accordance with PLOS ONE’s animal research reporting standards. These details lend credibility to the experimental design.

We thank the reviewer for the suggestion. The number of mice in each group, as well as whether mice succumbed to disease are now included in the figure legends.

We have also included information on assignment and blinding during experiments which can be found in the methods. Page 23, Line 559: “Mice were randomly assigned to each treatment group after confirmation of the tumor implantation before treatment. Investigators were not blinded during outcome assessment.”

• Clarity of definitions: Some terms in the Methods should be explicitly defined. For instance, “central lymph nodes (CLN)” are mentioned in the Results, seemingly referring to lymph nodes that are not sentinel (possibly contralateral nodes). However, this term is not standard. In the Lymphatics mapping or Flow Cytometry section, define what you mean by “sentinel lymph node (SLN)” and “central (or contralateral) lymph nodes (CLN)” so that readers understand which nodes were analyzed. Similarly, when describing the 4MOSC1 CD47 KO Cas9 KO cell line, clarify the process in a stepwise manner. The current description is dense (lines 985–993). Breaking it into steps (transduction with Cas9, selection, transduction with sgRNA, excision of Cas9 with Cre, etc.) or a figure in supplementary could help readers replicate this.

We thank the reviewer for the suggestion. We have appropriately defined what SLN and CLN refer to when it is first introduced in the lymphatic mapping section of the manuscript. Page 12, Line 269: “To better understand the mechanism by which anti-CD47 ICI can provide antitumor immunity, we characterized tumor, sentinel lymph nodes (SLN, the first lymph node that receives lymph flow from the primary tumor in a regional lymphatic basin), and contralateral lymph nodes (CLN, a lymph node from the regional lymphatic basin on the contralateral side) on a cellular level.”

For the 4MOSC1 CD47 KO Cas9KO cell line, we have outlined the process in the methods section of the manuscript and included a sentence to help with clarity that it can be found there. Page 6, Line 128: “To define the role of CD47 in this model, we created a CD47 knockout from our 4MOSC1 cell line to investigate the effect on tumor growth and the TME, with the protocol and primer sequences outlined in Methods.”

• Statistical analysis: The Statistics subsection indicates use of t-tests and ANOVAs, but please be more specific to strengthen the rigor. For example, specify which statistical tests correspond to which data (e.g., tumor volume comparisons over time likely used two-way ANOVA with repeated measures, survival used log-rank test, etc.). Also state how multiple comparisons were handled – e.g., “one-way ANOVA with Tukey’s post-hoc test” (or Bonferroni/Sidak as appropriate) – to show that you accounted for false positives. Indicate the threshold for significance (typically p < 0.05) and ensure it’s clear that you report mean ± SEM. (Minor grammar note: “All data are reported as mean ± SEM” since data is plural.) Additionally, in the figure legends you list p-values with asterisks; provide a key for what the asterisks denote (e.g., p<0.05) either in Methods or in a global figure legend note, so readers know how to interpret them.

We thank the reviewer for the suggestion. We have specified the statistical test used for each experiment in the figure caption. Example from figure 1 caption (Page 14, Line 331): “Data are presented as mean ± SEM; p values were calculated using ordinary one-way ANOVA with Tukey’s post-hoc.”

We have specified the threshold for significance and asterisks key in the methods of our manuscript. Page 26, Line 624: “All data are reported as mean ± SEM. All alpha thresholds were 0.05 and all confidence intervals were 95%. For P-values in figures, a P value of less than 0.0001 is denoted as ****, a P value between 0.0001 and 0.001 is denoted as ***, a P value between 0.001 and 0.01 is denoted as **, and a P value between 0.01 and 0.05 is denoted as *. Samples used in experiments were biological replicates.”

We have included all p values in the figure captions, including non-significant p values.

• Reagent and protocol details: Overall, the Methods are detailed, which is great. Just make sure all reagents and protocols are referenced or described briefly. For example, you mention “according to the STAR protocol with some modifications [35]” for immunophenotyping – consider summarizing what was modified (e.g., incubation times, gating strategy) or specifying that details are in a supplemental methods if applicable. Also, provide catalog numbers or sources for key reagents (you did so for some, like the isosulfan blue and antibodies – ensure this is consistent). If any critical experimental detail is in the Supplementary Information (for instance, sometimes detailed primer sequences or plasmid construction steps might be in supporting files), mention that clearly (e.g., “see Supplementary Methods for XYZ”). This transparency aligns with PLOSONE’s standards for reproducibility.

We thank the reviewer for the suggestion. We have clarified that the STAR Protocol’s modifications were addressed in the methods following where it was mentioned, Page 25, Line 600: “Immunophenotyping of tumor-bearing mice was performed according to the STAR protocol with the following modifications [37]. Briefly, tumor tissue, sentinel lymph nodes, and non-sentinel lymph nodes were mapped and harvested using lymphazurin.”

We have gone back and included the catalog numbers for all key reagents to be more consistent.

We ensured to mention what experimental details are included in supplemental information, such as the western blot for Cas9 confirmation in the 4MOSC cell line, Page 23, Line 538: “Cas9 expression by 4MOSC1 was verified via western blot (S1 Fig 1B)”

Results
• Consider using subheadings or clear paragraph breaks for each major experiment or theme. For example, you might insert italicized sub-section titles (if allowed) like “CD47 expression correlates with poor prognosis,” “CD47 knockout enhances anti-PD1 therapy,” “Combination anti-CD47 + anti-PD1 therapy (4MOSC1 vs 4MOSC2),” “Radiotherapy augments combination therapy,” and “Immune microenvironment and TCR clonality.” This will guide the reader and make the dense information more navigable. At minimum, ensure each new figure or experiment starts on a new paragraph with a topic sentence that introduces that result.

We thank the reviewer for this suggestion. We have added subheadings to make clear what was the focus of each experiment and what we found.

• “Fig 2C shows that the introduction of tdRT to the treatment allows further tumor regression in mice with the 4MOSC2 tumor model.” – This phrasing is a bit informal. Replace “introduction of tdRT to the treatment” with a clearer description. For instance: “Figure 2C shows that adding tumor-directed radiotherapy (tdRT) to the treatment regimen led to further tumor regression in the 4MOSC2 model.” This explicitly states what was done (tdRT added) and the outcome. Similarly, later in that paragraph: “Of note, enhanced long-term survivability was associated with anti-CD47 as a part of treatment” can be tightened. E.g., “Notably, only treatment groups that included anti-CD47 showed significantly prolonged survival, underlining the contribution of CD47 blockade to improved outcomes.” Strive for a consistent tone that is factual and precise, avoiding colloquial phrases like “of note” or “with interest” in the formal results narrative.

We thank the reviewer for the suggestions. We have reworded the results to be more formal.

Page 9, Line 208: “Fig 2C shows that adding tumor-directed radiation therapy (tdRT) to the treatment regimen led to further tumor regression in the 4MOSC2 model.”

Page 9, Line 212: “Notably, only treatment groups that included ALX301 showed significantly prolonged survival, underlining the contribution of CD47 blockade to improved outcomes. A comparison between ALX301 and tdRT therapies in the 4MOSC2 model as monotherapies and combinatory therapies was performed showing partial reduction with tdRT as a monotherapy and significant reduction with ALX301 as dual therapy (S1 Fig 1G).”

• Define responses and endpoints: You frequently use terms like “complete tumor regression”, “partial tumor regression,” and “significant tumor regression.” It’s important to define these within the context of your models. For example, clarify what criteria were used to declare a complete regression (tumor not palpable/visible at a certain timepoint, and for how long?). If a partial regression means tumors shrank but did not fully disappear, mention the average reduction or time to regrowth. Providing this context (possibly in a sentence or in figure legends) will prevent misunderstanding. For instance: “…led to complete tumor regression (no detectable tumor in 100% of mice by Day 30, maintained for the duration of observation), whereas in the 4MOSC2 model tumors only partially regressed (shrank by ~50% but never fully disappeared).” Such details, even if approximate, greatly help interpret the efficacy of the treatments.

We thank the reviewer for the suggestion. We have defined what partial regression vs complete tumor regression means in the text and in the figure captions included how many mice had complete tumor eradication, tumor reduction, and continued tumor growth.

Page 6, Line 135: “resulting in complete tumor regression, defined as complete or almost complete tumor eradication”

Page 9, Line 196: “with partial tumor regression defined as tumor reduction but not complete tumor eradication”

Page 7, Line 166: “In the 4MOSC1 Parent group, 2/10 mice had a partial reduction in tumor size, while 8/10 had continuous tumor growth. In the 4MOSC1 Parent + anti-PD1 group, 3/10 mice had complete tumor eradication, 6/10 mice had tumor reduction, and 1/10 mice had continuous tumor growth. In the 4MOSC1 CD47KO group, 10/10 mice had complete tumor eradication. In the 4MOSC1 CD47KO + anti-PD1 group, 9/10 mice had complete tumor eradication, and 1/10 mice had tumor reduction.”

• Ensure data support claims: When stating outcomes in text, make sure to mention significance if applicable. You do report p-values in the figure legends (e.g., “control vs. anti-PD1+anti-CD47 p=0.0114” in Fig. 2 legend) – it’s good to also refer to significance in the text. For example, instead of “demonstrated significant tumor regression” alone, you could write “demonstrated significantly greater tumor regression than controls (Fig. 2B, p<0.05)”. Even a brief mention like “significant” with a figure reference is helpful, but tie it to the data. Also, for multi-group comparisons, clarify what is being compared: readers should know if “significant tumor regression” refers to compared to untreated controls, or compared to single-agent treatments. In Figure 2, for instance, explicitly noting that the triple therapy outperformed both single agents in text would be useful.

We thank the reviewer for the suggestions. We have included in the text p values when appropriate and clarified what is being compared.

For example, Page 6, Line 135: “We observed that knocking CD47 out of 4MOSC1 sensitizes the cell line, resulting in complete tumor regression, defined as complete or almost complete tumor eradication (Fig 1D, parent vs. CD47KO p < 0.0001). Using the CD47 KO Cas9 KO 4MOSC1 cell line, we further observed complete tumor regression in the knockout groups, whereas the parent 4MOSC1 cell line tumors continued to grow (Fig 1E, parent vs. Cas9KO CD47KO p < 0.0001).”

• “We observed with interest that, when tumors were treated with anti-CD47, there was an increase in total T-cell clonal expansion… and in the fraction of clones within their own repertoire as well as an increase in shared clonality between the tumor and SLN… This is consistent with an enhanced clonal T cell response generated in draining SLN with migration of T cells from the draining SLN to TME.” – This is an exciting finding about T-cell clonality, but the wording can be made more concise and objective. Remove “with interest” and simply state the observation: “We observed that anti-CD47 treatment increased overall T-cell clonal expansion and the fraction of dominant T-cell clones, as well as increased shared TCR clonality between tumor and SLN.” Then, ensure the interpretation is clearly linked: “This finding suggests a more robust, shared antigen-specific T-cell response developing in the lymph node and homing to the tumor.” Additionally, acknowledge sample size limitations here: it appears TCR-seq was done on one tumor/LN per group. It’s worth noting in the text or discussion that these clonality observations, while suggestive, are based on limited sampling (if true), to temper the interpretation.

We thank the reviewer for these suggestions. We have reworded this section to be more concise and objective, while including the limitation of the sampling size for transparency. Page 18, Line 432: “we observed that ALX301 treatment increased overall T-cell clonal expansion and the fraction of dominant T-cell clones, as well as increased shared TCR clonality between tumor and SLN. This finding suggests a more robust, shared antigen-specific T-cell response developing in the lymph node and homing to the tumor. It is important to note that these observations, while suggestive of our hypothesis that ALX301 increases trafficking and migration of APCs, are based on limited sampling.”

• Figure references and labels: Double-check that every figure is cited in order and that lettering is consistent. For example, you mention “(Fig 3C)” and “(Fig 3D)” for MHC II and CD86 upregulation – ensure that those sub-panels indeed correspond to the described data (the text and figure legends seem to align well for Fig 3). Also, consider referencing supplemental figures in the Results text where relevant. You mention “S1 Fig 1C” in the legend for Fig 2 (line 1091–1092 in the PDF), implying there are supplementary figures. Make sure to cite them in the main text at appropriate points (e.g., if S1 Fig shows additional controls or data, say so in Results). This helps readers know supplemental data exists without having to find it on their own.

We thank the reviewer for the suggestions. We have gone back to ensure the figure lettering is consistent and adheres to PLOS One format. We have also made sure to reference the supplemental figures when appropriate.

Discussion
• “Given these considerations, we designed a new combinatory therapy for our study.” – Minor wording: “combinatory” is not commonly used; “combinatorial” or “combined” would be clearer. For instance: “…we designed a new combinatorial therapy approach in our study.”

We thank the reviewer for this suggestion. We have reworded this section for clarity. Page 17, Line 395: “…we designed a new combinatorial therapy approach in our study.”

• “These data suggest a need for other types of therapies that are just as or more effective whether alone or in combination with ICIs [29].” – This sentence can be rephrased for clarity and grammar. Consider: “These data suggest that we need additional therapies that can be as effective as, or more effective than, current options – either used alone or in combination with ICIs [29].” This eliminates the colloquial “just as or more” phrasing.

We thank the reviewer for this suggestion. We have reworded the sentence to avoid the colloquial phrasing. Page 16, Line 384: “These data suggest that we need additional therapies that can be as effective as, or more effective than, current options – either used alone or in combination with ICIs”

• “Clinical trials have also demonstrated that sequencing of therapies are important, for example, when anti-PD1 is delivered before chemoradiotherapy (CRT), there is an abrogated immune response… whereas if anti-PD1 is delivered after CRT, there is better control… [29].” – There’s a subject-verb agreement issue (“sequencing … are important”) and the sentence is long. Split it for readability: “Clinical trials also demonstrate the importance of treatment sequencing. For example, delivering anti–PD-1 before chemoradiotherapy (CRT) can abrogate the immune response (due to radiosensitive CD8 T cells), whereas delivering anti–PD-1 after CRT yields better tumor control [29].” This way, the point about sequencing stands out, and the grammar is corrected (“sequencing… is important”).

We thank the reviewer for this suggestion. We have corrected the readability and grammar as recommended. Page 16, Line 386: “Clinical trials also demonstrate the importance of treatment sequencing. For example, delivering anti–PD1 before chemoradiotherapy (CRT) can abrogate the immune response (due to radiosensitive CD8 T cells), whereas delivering anti–PD1 after CRT yields better tumor control [30].”

• “CD47 has long been studied as a potential target… expressed on multiple human tumor types… allows tumors to evade the immune system by binding SIRPα, allowing tumor cells to express the ‘Don’t Eat Me’ signal to evade phagocytosis… [31].” – This background in the Discussion reiterates known information. While it is useful to a point, consider condensing it to avoid redundancy with the Introduction. For instance: “CD47 is an immune checkpoint that delivers a ‘Don’t Eat Me’ signal to phagocytes via SIRPα binding, and it is overexpressed in many tumors to enable immune evasion [19,31].” This single sentence could replace a few lines of text. Keeping the discussion focused on interpreting your findings in light of this mechanism (rather than re-explaining the mechanism at length) will make it more impactful.

We thank the reviewer for this suggestion. We have removed the re-explanation of the mechanism and replaced it with the single sentence as recommended.

• Interpretation of your results: The Discussion does a good job summarizing the experiments (CD47 KO, ALX301 + PD-1 therapy in two models, etc.) and tying them to the big picture. In a few places, the wording could be tightened. For example, “using both anti-PD1 and anti-CD47 showed significant response, and the addition of anti-CD47 enhanced survival” is a bit confusing because “using both” already implies anti-CD47 is there. You likely mean that in the PD-1–resistant model, anti-CD47 plus anti-PD1 yielded a significant anti-tumor response, and adding radiotherapy further improved survival. Make sure each combination’s benefit is clearly distinguished. You might rewrite that part as: “In our PD-1–resistant 4MOSC2 model, the combination of anti-CD47 with anti-PD1 produced a significant tumor response where anti-PD1 alone had minimal effect. Notably, adding tumor-directed radiotherapy to this combination further prolonged survival.” This explicitly states each component’s contribution.

We thank the reviewer for this suggestion. Our interpretation of the data was not that tdRT enhanced survivability as the anti-PD1+tdRT group was not statistically significant when compared to the control. Only groups that were treated with anti-CD47 demonstrated statistically significant increase in survivability. To ensure better clarity of these results, this section was reworded as follows (Page 18, Line 424): “In our anti-PD1 resistant 4MOSC2 model, using dual therapy of anti-PD1 and ALX301 showed significant response compared to the control (Fig 2C, p = 0.0007), and groups with ALX301 as part of their treatment demonstrated enhanced survival.”

• Tone down overgeneralization: While your results are promising, avoid overstating the translational impact. For instance, “the benefits of CD47 inhibition as a combination therapy reveal its promise as a future HNSCC treatment” is optimistic but could be softened to reflect that this is preclinical evidence. You might say: “…reveal its promise as part of future HNSCC treatment strategies, pending clinical validation.” Similarly, when concluding that you “provided a basis for rational combination of these therapies,” ensure you also acknowledge that further studies (especially clinical trials) are needed to confirm safety and efficacy in patients. PLOS ONE typically values conclusions that are supported by the data without speculation beyond what the results show.

We thank the reviewer for their suggestion and have reworded these sections such that they are not overly optimistic but rather are based on conclusions supported by the data.

Page 19, Line 465: “As we have demonstrated in our own study, the benefits of CD47 inhibition as a combination therapy reveal its promise as part of future HNSCC treatment strategies, pending clinical validation.”

Page 19, Line 441: “By demonstrating that anti-PD1 and radiotherapy can be further optimized in combination with anti-CD47, we have provided a basis for rational combination of these therapies in future clinical trials to enhance tumor response.”

• Expand on limitations: You appropriately mention some limitations (e.g., the short duration precluded toxicity assessment, and animal models don’t capture the full clinical complexity). It would strengthen the Discussion to elaborate briefly on these and any other limitations. For example, discuss the limitation of using only one tumor model for each scenario (4MOSC1 for responsive, 4MOSC2 for resistant – how generalizable might this be to other HNSCC tumors?). Also, the TCR sequencing was done on a limited number of samples; acknowledging that as a preliminary observation would be prudent. You could add: “Another limitation is the T-cell receptor sequencing analysis, which was performed on a single sample per group, providing a snapshot of clonality but not statistics. Future studies with larger sample sizes are warranted to confirm the observed clonal expansion patterns.” By openly discussing such issues, you align with PLOS ONE’s standards for transparency and encourage follow-up research.

We thank the reviewer for these suggestions. We have expanded the limitations section to include toxicity, the limitation of animal models, the limitation of using only the 4MOSC cell line, and the limitation of the TCR Seq sample size.

Page 19, Line 447: “Past studies have demonstrated that CD47 blockade expresses minimal drug toxicity due to the lack of pro-phagocytic signals on healthy cells [34]. Throughout our study, no toxicity was observed in mice who received ALX301 therapy. It is important to note that while mice and humans share many aspects of cellular identity, key differences exist that impact the immune response to cancer and CD47 blockade [35]. Such differences include expression and binding of selectins, cytokine production, and Fc receptor expression [35]. Therefore, future studies that investigate the long-term toxicity profile of anti-CD47 are warranted to ensure safety during prolonged treatment in a clinical setting. Additionally, while the 4MOSC model displays the mutational profile, immune landscape, and ICI responsiveness of HPV-negative HNSCC, our findings may not extend to all HPV-negative tumors. Another limitation is the T-cell receptor sequencing analysis, which was performed on a single sample per group. These results provide a snapshot of clonality to provide insight into the flow cytometry results of the tumors and lymph nodes, but not statistical results. Future studies with larger sample sizes are warranted to confirm the observed clonal expansion patterns with statistical comparisons.”

• Language and style: Go through the Discussion for minor grammar issues and repetition. For instance, avoid starting sentences with conjunctions frequently (e.g., many sentences begin with “However,” – combine ideas if possible). Also ensure the past tense is used when referring to your results (“we found,” “we observed”) and present tense for general knowledge (“CD47 is…,” “these data suggest…”), which you generally do. One example: “CD47 inhibition has shown great results synergistically with other therapies as well.” Since this refers to past studies, it could be “CD47 inhibition has also synergized with other therapies in preclinical studies.” Small tweaks like this improve clarity.

We thank the reviewer for this suggestion. We have reviewed the manuscript and have deleted many of the unnecessary “however”, ensured past tense is used when referring to our results and present tense for general knowledge.

Conclusions

**Reviewer #7:** 1. There are multiple typo errors that need to be edited by a native speaker.

We thank the reviewer for this suggestion. The manuscript has been reviewed to fix the typo errors.
2. The section "TCR Sequencing subsection" needs to be expanded.

We thank the reviewer for this suggestion. The TCR Sequencing section has been expanded to include clarification of the sample size and additional information of the results.

Page 14, Line 338: “To match the results of the results of the TCR seq to the results of the flow cytometry, one tumor and SLN were extracted from each group”

Page 15, Line 345: “Notably, in 4MOSC2 models, we saw greater shared clonality between the tumors and SLNs in mice treated with ALX301, and a greater Morisita overlap index between 4MOSC2 tumor and SLN in the anti-PD1 + ALX301 treated arms (Figs 5D, 5E, and 6).”

3. Result should be re-written based on subtitles.

We thank the reviewer for this suggestion. We have added subheadings to make clear what was the focus of each experiment and what we found.

4. The comparison between ALX301 and ALX148 or other agents should be better described.

We thank the reviewer for this suggestion. ALX301 is the murine-specific anti-CD47 and ALX148 is the human-specific anti-CD47. We have edited the manuscript to make that clear, specifically adding the clarification to the introduction and discussion where ALX148 is mentioned.

Page 5, Line 97: “Some researchers have identified routes around this limitation by using a clinical grade engineered CD47 inhibitor with an inactive Fc region, evorpacept (ALX148), to avoid hematological toxicity [20].”

Page 5, Line 103: “In this study, we evaluate the effects of a mouse-specific engineered CD47-blocking SIRPα fusion protein (ALX301)…”

Page 17, Line 402: “Based on these findings, a clinical grade CD47-blocking myeloid checkpoint inhibitor, evorpacept (ALX148), was developed to promote phagocytosis of tumor cells.”

5. A tdRT-only arm and a CD47-only (without anti-PD-1) + tdRT group should be described or justified if omitted in the result section.

We thank the reviewer for this suggestion. We have done an experiment that compares tdRT and CD47 as monotherapies and dual therapies in the 4MOSC2 cell line. These results were included in the Supplemental Figure 1G and were briefly mentioned in the manuscript.

Page 9, Line 214: “A comparison between ALX301 and tdRT therapies in the 4MOSC2 model as monotherapies and combinatory therapies was performed showing partial reduction with tdRT as a monotherapy and significant reduction with ALX301 as dual therapy (S1 Fig 1G).”

6. “anti-CD47,” “CD47 blockade,” and “ALX301” are used interchangeably.

We thank the reviewer for this observation. ALX301 is the name of the mouse specific anti-CD47 ICI used in our study. We have revised the manuscript and figure labels to specify ALX301 when referring to our experimental agent, and “anti-CD47” and “CD47 Blockade” when discussing the broader literature.

7. Do statistical comparisons in TCR sequencing analysis.

We thank the reviewer for this suggestion. Unfortunately, because the TCR Seq was done with one sample from each group, we don’t have enough replicates to perform a reliable statistical comparison. This limitation is addressed in our manuscript.

Page 14, Line 338: “To match the results of the results of the TCR seq to the results of the flow cytometry, one tumor and SLN were extracted from each group.”

Page 18, Line 432: “After performing TCR sequencing on these models, we observed that ALX301 treatment increased overall T-cell clonal expansion and the fraction of dominant T-cell clones, as well as increased shared TCR clonality between tumor and SLN. This finding suggests a more robust, shared antigen-specific T-cell response developing in the lymph node and homing to the tumor. It is important to note that these observations, while suggestive of our hypothesis that ALX301 increases trafficking and migration of APCs, are based on limited sampling.”

Page 19, Line 457: “Another limitation is the T-cell receptor sequencing analysis, which was performed on a single sample per group. These results provide a snapshot of clonality to provide insight into the flow cytometry results of the tumors and lymph nodes, but not statistical results. Future studies with larger sample sizes are warranted to confirm the observed clonal expansion patterns with statistical comparisons.”

8. Toxicity concerns of systemic CD47 inhibition in humans, and the immunological differences between mice and humans should be more discussed in the discussion.

We thank the reviewer for this suggestion. We have expanded the limitations section in our discussion to better address toxicity concerns of CD47 inhibition in humans and the immunological differences between mice and humans.

Page 19, Line 447: “Past studies have demonstrated that CD47 blockade expresses minimal drug toxicity due to the lack of pro-phagocytic signals on healthy cells [34]. Throughout our study, no toxicity was observed in mice who received ALX301 therapy. It is important to note that while mice and humans share many aspects of cellular identity, key differences exist that impact the immune response to cancer and CD47 blockade [35]. Such differences include expression and binding of selectins, cytokine production, and Fc receptor expression [35]. Therefore, future studies that investigate the long-term toxicity profile of anti-CD47 are warranted to ensure safety during prolonged treatment in a clinical setting.”
